# Pectiniferosides A–J: Diversified Glycosides of Polyhydroxy Steroids Isolated from the Sea Star *Patiria (=Asterina) pectinifera*

**DOI:** 10.3390/md22120545

**Published:** 2024-12-03

**Authors:** Ranran Zhang, Zhen Lu, Derui Wang, Zhi Yan, Xueting Sun, Xiaodong Li, Xiuli Yin, Song Wang, Ke Li

**Affiliations:** 1 Yantai Institute of Coastal Zone Research, Chinese Academy of Sciences, Yantai 264003, China; rrzhang@yic.ac.cn (R.Z.); zlu@yic.ac.cn (Z.L.); zyan@yic.ac.cn (Z.Y.); sunxueting233@163.com (X.S.); xiaodongli@yic.ac.cn (X.L.); xlyin@yic.ac.cn (X.Y.); 2 College of Resources and Environment, University of Chinese Academy of Sciences, Beijing 100049, China; 3 College of Marine Science, Beibu Gulf University, Qinzhou 535011, China; wdrui@bbgu.edu.cn; 4 Co-Innovation Center of Jiangsu Marine Bio-industry Technology, Jiangsu Ocean University, Lianyungang 222005, China; wangsong75@163.com; 5 Center for Ocean Mega-Science, Chinese Academy of Sciences, Qingdao 266071, China

**Keywords:** *Patiria (=Asterina) pectinifera*, steroidal glycosides, embryotoxicity, cytotoxicity

## Abstract

To optimize the utilization of the sea star *Patiria (=Asterina) pectinifera*, which has demonstrated potential pharmaceutical properties in Chinese folk medicine, ten glycosides of polyhydroxy steroids, pectiniferosides A–J (**1**–**10**), were isolated and characterized. These compounds possess 3β, 6α, 8, 15α (or β), 16β-pentahydroxycholestane aglycones with sulfated and (or) methylated monosaccharides. The chemical structures of **1**–**10** were determined using NMR spectroscopy and HR-ESI-MS. The discovery of saponins with multiple substitution patterns in sea stars exemplified the molecular diversity of secondary metabolites in marine echinoderms. These compounds exhibited no embryotoxicity or teratogenicity at a concentration of 100 μM in a bioassay with marine medaka (*Oryzias melastigma*) embryos, implying that these compounds may not be ecologically toxic to marine fish embryos. In addition, none of the compounds exhibited significant cytotoxicity against five human cancer cell lines at 40 μM or anti-inflammatory activities at 50 μM, suggesting their potential for further structural optimization to enhance bioactivity. The research on the constituents of *P. pectinifera* provides a potential foundation for drug development and marine ecotoxicology.

## 1. Introduction

Sea stars, carnivorous benthic invertebrates belonging to the class Asteroidea in the phylum Echinodermata, possess a diverse diet, preying on species from the phyla Mollusca, Echinodermata, Arthropoda, Cnidaria, and Chordata, and consuming organic detritus. Based on their outstanding vitality, regenerative capability, and relative lack of natural enemies, sea star outbreaks can cause deteriorating changes to the structure of benthic marine communities as well as substantial economic losses [1]. Sea star outbreaks, such as those caused by *P. pectinifera*, pose a serious ecological threat by destabilizing benthic marine ecosystems, often resulting in the loss of biodiversity and the collapse of critical habitats like coral reefs. Economically, these outbreaks can devastate fisheries and aquaculture industries, significantly reducing the production of valuable species like sea cucumbers and scallops, highlighting the importance of studying *P. pectinifera* metabolites for potential management or mitigation strategies. Sea star extracts have showed significant predator-repellent activities [2], while their tissue fluids have also influenced the activity of their prey [3,4]. Therefore, the metabolites of sea stars appear to fulfill both defensive and offensive functions.

Chinese folk medicine has demonstrated sea stars’ various pharmaceutical properties. In previous studies, diverse secondary metabolites, including steroids, steroidal glycosides, alkaloids, phospholipids, peptides, and fatty acids [5,6], have been discovered and have shown a wide range of biological functions, including hemolytic effects, cytotoxicity [7,8], anti-inflammatory properties [9], and antifungal and antimicrobial activities [10]. Recently, disulfated ophiuroid-type steroids isolated from the sea star *Pteraster marsippus* demonstrated significant cytotoxic activity against human breast cancer cells [11,12]. Notably, the different components of sea stars were found in typical organs [13]. Sea stars’ first and most abundant secondary metabolites are steroidal glycosides, often referred to as saponins. Saponins are a class of naturally occurring amphiphilic glycosides with a wide range of bioactivities, including antifungal, cytotoxic, cardioprotective, and anti-inflammatory activities [14,15]. While most saponins are derived from plants, their production within the animal kingdom is restricted to three taxonomic classes: Holothuroidea and Asteroidea of the phylum Echinodermata, and Demospongiae sponges of the phylum Porifera [16]. Sea stars, carnivorous benthic invertebrates, belong to the class Asteroidea in the phylum Echinodermata. Given their structural diversity, the saponins of the sea star *P. pectinifera* warrant continued investigation. To date, research on compounds isolated from *P. pectinifera* has primarily focused on their potential pharmacological activities, including cytotoxic, antimicrobial, and anti-inflammatory effects [17], with relatively few studies exploring their ecological functions.

Saponin structures are classified into glycosides of polyhydroxy steroids, asterosaponins, and cyclic steroidal glycosides. The aglycones of polyhydroxy steroid glycoside are usually characterized as 3β, 6α (or β), 8, 15α (or β), or 16β-pentahydroxycholestane in sulfated or free hydroxy forms [5]. In our previous research, polyhydroxy steroids were found from *P. pectinifera* [18], which is subject to outbreaks in the Yellow Sea and the Bohai Sea of China. The structural diversity of secondary metabolites may offer potential for drug development and serve as a novel strategy for the high-value utilization of emerging species.

**Figure 1:**
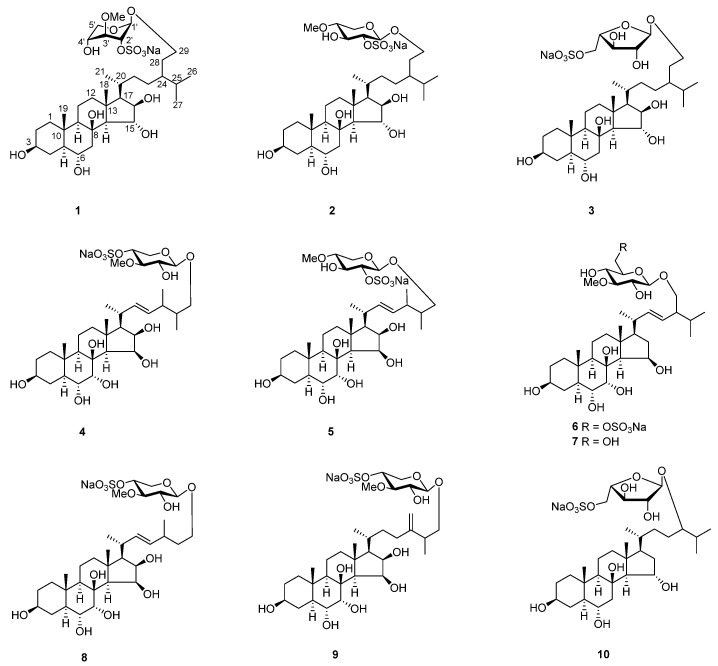
Chemical structures of compounds **1**–**10**.

In this continuation of our investigation into sea stars (*P. pectinifera*), we characterized ten new glycosides of polyhydroxy steroids, pectiniferosides A–J (Figure 1). Their embryotoxicity and teratogenicity were evaluated using embryos of marine medaka (*Oryzias melastigma*), a fish model known for its significant role in marine ecotoxicological studies [19] with demonstrated advantages in bioassays [20]. Additionally, their cytotoxicity against five human cancer cell lines and their anti-inflammatory activities in RAW264.7 cells stimulated by lipopolysaccharide (LPS) were also evaluated.

## 2. Results and Discussion

**Figure 2:**
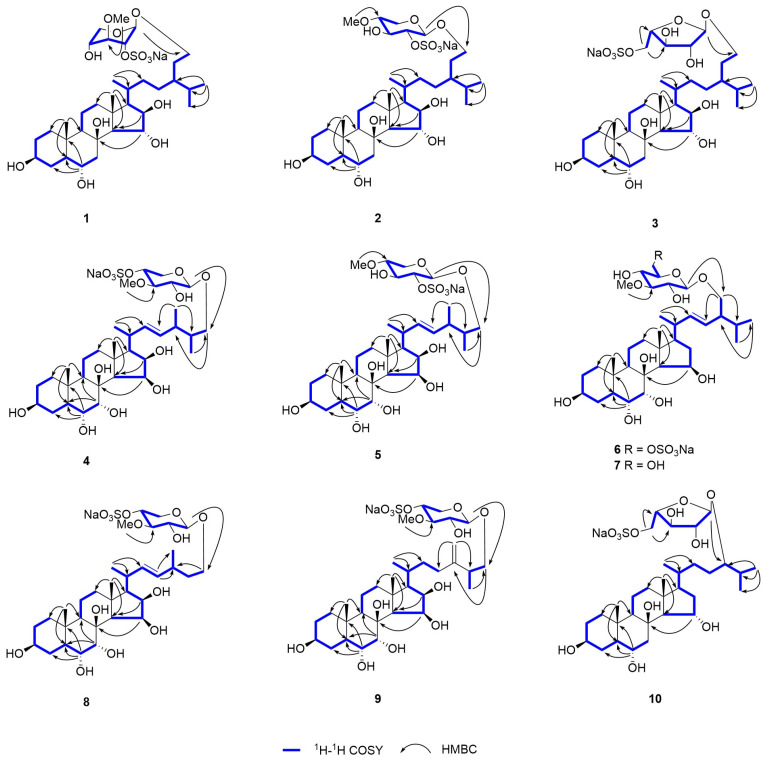
The key ^1^H-^1^H COSY and HMBC correlations for compounds **1**–**10**.

Pectiniferoside A (**1**) was isolated as a colorless gum. The molecular formula of **1** was determined as C_35_H_61_O_13_SNa from the sodium adduct ion peak at *m*/*z* 767.3646 [M + Na]^+^ (calcd for C_35_H_61_O_13_SNa_2_^+^, 767.3623) in the positive mode and from the ion peak at *m*/*z* 721.3866 [M − Na]^−^ (calcd for C_35_H_61_O_13_S^−^, 721.3838) in the negative mode HR-ESI-MS spectra. A fragment ion peak at *m*/*z* 96.9 indicated a sulfate ester group in **1**. The fragment ion peak at m/z 495 [M − Na − 226]^−^ in the tandem mass spectrum (Figure S1) was reconciled by the loss of the monosaccharide moiety. The one-dimensional (1D) NMR (Table 1) and HSQC spectra revealed two angular methyls for CH_3_-18 (*δ*_H_ 1.12, s; *δ*_C_ 17.0) and CH_3_-19 (*δ*_H_ 1.02, s; *δ*_C_ 14.2), and three doublet methyls for CH_3_-21 (*δ*_H_ 0.93, d, *J* = 7.0 Hz; *δ*_C_ 18.5), CH_3_-26 (*δ*_H_ 0.87, d, *J* = 7.0 Hz; *δ*_C_ 19.9), and CH_3_-27 (*δ*_H_ 0.85, d, *J* = 6.5 Hz; *δ*_C_ 19.1). The ^13^C NMR and DEPT spectra showed four oxygenated methine carbon resonances at *δ*_C_ 72.2 (C-3), 67.7 (C-6), 80.7 (C-15), and 82.9 (C-16), one oxygenated tertiary carbon signal at *δ*_C_ 75.8 (C-8), and one oxygenated methylene signal at *δ*_C_ 68.9 (C-29). The chemical shifts from C-1 to C-29 in ^13^C NMR of **1** were in good agreement with those of oreasteroside F isolated from the sea star *Oreaster reticulatus* [21], and the Δ*δ*_C_ between 1 and oreasteroside F were in −2~2 ppm. The correlations between protons at C-1 and C-7, C-9 and C-12, C-14 and C-21,and C-21 and C-29 through C-24 in the ^1^H-^1^H COSY spectrum (Figure 2), along with correlations from H_3_-26 to C-24/C-25/C-27, H_3_-21 to C-20/C-22, H_3_-19 to C-1/C-5/C-9, H_3_-18 to C-12/C-14/C-17, H-15 to C-8, and H-6 to C-4/C-5/C-10 in the HMBC spectrum, further confirmed the aglycone of 24-ethyl-5α-cholestane-3β,6α,8,15α,16β,29-hexaol. Characteristic signals for pentose, including one anomeric carbon, three oxymethine carbons, and one methylene closing out the spin system, were observed. Moreover, the correlations of protons at C-1′ to C-5′ in the ^1^H-^1^H COSY spectrum, as well as the correlation between H_3_-OMe and C-3′ in the HMBC spectrum, indicated the presence of a 3-*O*-methylpentose moiety. When comparing the chemical shifts and coupling constants of pentopyranosides between **1** and oreasteroside K [21] in ^1^H NMR, compound **1** should contain a 3-*O*-methyl-2-*O*-sulfated-β-xylopyranosyl moiety. The connection of the sugar moiety to the steroidal aglycone at C-29 was established by analyzing the HMBC correlation from H-1′ to C-29.

**Figure 3:**
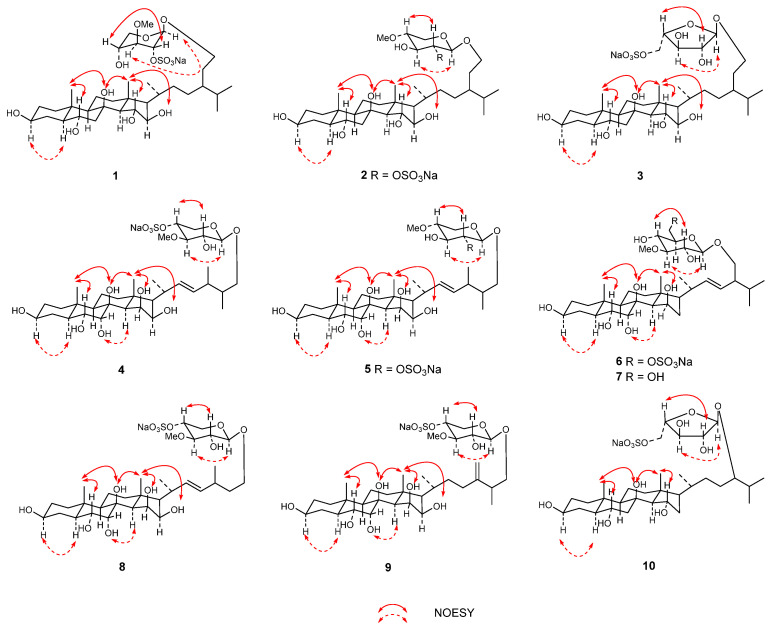
The key NOESY correlations for compounds **1**–**10**.

The relative configuration of **1** was determined using NOESY (Figure 3) and coupling constant analysis. The results showed correlations between H-6 and H_3_-19, between H-3 and H-5, and between H-15 and H_3_-18. Therefore, the β-configurations of H-6 and H-15 were established, and the α-configuration of H-3 was determined. Nevertheless, the relative configurations of C-8 and C-16 could not be confirmed by NOESY correlations. DMSO-*d*_6_ was chosen as the solvent to observe the active hydrogen signals of hydroxy groups, as its low chemical exchange rate prevents the replacement of active hydrogens in the sample. This enabled the confirmation of the relative configurations of carbons connected to hydroxy groups through NOE correlations. Compound **1** exhibited exchangeable protons in DMSO-*d*_6_ (Table 2). The results revealed that the correlations from OH-8 to H_3_-18 and H_3_-19, as well as OH-16 to H_3_-18, indicated that the relative configurations of OH-8 and OH-16 were β-oriented. In addition, the NOESY correlations of H-1′ to H-3′ and H-2′ to H-4′ also supported the presence of the 3-*O*-methyl-2-*O*-sulfated-β-xylopyranosyl moiety in **1**. The deviation of the coupling constant values of anomeric proton H-1′ (*δ*_H_ 4.70, d, *J* = 3.5 Hz) of **1** from those usually observed for the β-xylopyranosyl moiety (*J* = 7.6 Hz) [22] was analyzed by Gluseppe et al. using molecular dynamics and mechanics calculations [21], and the results showed that the model methyl-3-*O*-methyl-2-*O*-sulfated-β-xylopyranoside existed as an equilibrium mixture of two conformers, with ^1^C_4_ (80%) and ^4^C_1_ (20%). The D-configuration of the sugar moiety was determined by comparing its specific rotation after acid hydrolysis with analog [23]. Furthermore, although the chemical shifts in C-24 to C-27 of **1** were in good agreement with oreasteroside F [21], the C-24 configuration of **1** was still difficult to determine unless each of the C-24 epimers had been synthesized. Based on these results, the structure of **1** was determined as a sodium salt of 29-*O*-(3-*O*-methyl-2-*O*-sulfated-β-D-xylopyranosyl)-24-ethyl-5α-cholestane-3β,6α,8β,15α,16β,29-hexaol.

Pectiniferoside B (**2**) was recognized as an isomer of **1** with the molecular formula of C_35_H_61_O_13_SNa based on the HR-ESI-MS ion peak at *m*/*z* 721.3828 [M − Na]^−^ (calcd for C_35_H_61_O_13_S^−^, 721.3838) and the ion peak at *m*/*z* 767.3630 [M + Na]^+^ (calcd for C_35_H_61_O_13_SNa_2_^+^, 767.3623). The assignment of 1D NMR (Table 1) and 2D NMR spectral data suggested that the only difference between **2** and **1** was the position of methoxy group, which was located at C-4′ (*δ*_C_ 80.1) in **2** instead of at C-3′ (*δ*_C_ 82.6), as in **1**. The correlation of H_3_-OMe to C-4′ in the HMBC spectrum (Figure 2) supported the position of the methoxy group at C-4′. The NOESY correlations (Figure 3), coupling constant analysis (*δ*_H_ 4.47, d, *J* = 6.5 Hz, H-1′) and optical rotation analysis, supported the presence of a 4-*O*-methyl-2-*O*-sulfated-β-D-xylopyranosyl moiety. Thus, the structure of **2** was determined as a sodium salt of 29-*O*-(4-*O*-methyl-2-*O*-sulfated-β-D-xylopyranosyl)-24-ethyl-5α-cholestane-3β,6α,8β,15α,16β,29-hexaol.

Pectiniferoside C (**3**) was isolated as a colorless gum; HR-ESI-MS signals at *m*/*z* 707.3715 [M − Na]^−^ (calcd for C_34_H_61_O_13_S^−^, 707.3682) and *m*/*z* 753.3473 [M + Na]^+^ (calcd for C_34_H_59_O_13_SNa_2_^+^, 753.3466) established the molecular formula C_34_H_61_O_13_SNa with five degrees of unsaturation. The fragment ion peak at *m*/*z* 495 [M − Na − 212]^−^ (Figure S20) in the tandem mass spectrum was responsible for the loss of sulfated pentose residue. The chemical shifts in C-1 to C-19 in ^13^C NMR (Table 1) of **3** showed strong similarity to **1**. The glycosylation shift observed for C-29 (*δ*_C_ 68.2) and HMBC correlations identified the location of the sugar moiety as the same as in **1** and **2**. The correlations of protons at C-1′ to C-5′ in ^1^H-^1^H COSY spectrum and from H_2_-5′ to C-4′ and C-3′ in the HMBC spectrum (Figure 2) revealed that the pentose in **3** was in its furanose form, while the obvious downfield shift in C-5′ (*δ*_C_ 68.6) and characteristic fragment ion *m*/*z* 96.9 confirming the sulfate group at C-5′. Furthermore, the 1D NMR spectra and NOESY (Figure 3) correlations showed signals for the 5-*O*-sulfated-α-L-arabinofuranosyl group similar to the monosaccharide signals of scoparioside A [24]. Acid hydrolysis and GC-MS analysis also confirmed this sugar moiety. Therefore, the chemical structure of **3** was established as a sodium salt of 29-*O*-(5-*O*-sulfated-α-L-arabinofuranosyl)-24-ethyl-5α-cholestane-3β,6α,8β,15α,16β,29-hexaol.

Pectiniferoside D (**4**) was isolated as a colorless gum and gave the molecular formula C_34_H_57_O_14_SNa by the HR-ESI-MS ion signals at *m*/*z* 721.3481 [M − Na]^−^ (calcd for C_34_H_57_O_14_S^−^, 721.3475) and *m*/*z* 767.3275 [M + Na]^+^ (calcd for C_34_H_57_O_14_SNa_2_^+^, 767.3259). The mass data indicated six indices of hydrogen deficiency. A fragment ion peak at *m*/*z* 96.9 (Figure S29) indicated a sulfate ester group in **4**. The 1D NMR spectra (Table 3) of four displayed signals of two angular methyls for CH_3_-18 (*δ*_H_ 1.28, s; *δ*_C_ 17.8) and CH_3_-19 (*δ*_H_ 0.99, s; *δ*_C_ 13.9), as well as three doublet methyls for CH_3_-21 (*δ*_H_ 1.04, d, *J* = 7.0 Hz; *δ*_C_ 20.3), CH_3_-27 (*δ*_H_ 0.92, d, *J* = 6.5 Hz; *δ*_C_ 14.9), and CH_3_-28 (*δ*_H_ 0.96, d, *J* = 7.0 Hz; *δ*_C_ 17.6), which were attributable to the typical steroid skeleton. Moreover, five oxygenated methines, one oxygenated methylene, one oxygenated tertiary carbon, and two *sp*^2^ methines were assigned using the DEPT spectra and HSQC correlations. Similarly, one anomeric carbon, three oxymethine carbons, and one methylene closing out a spin system within a xylose were observed. The 1D and 2D NMR spectra supported that **4** had the 3β,6α,7α,8,15β,16β-hexahydroxy tetracyclic steroidal nucleus, similar to oreasteroside K [21]. The signals of H-22 (*δ*_H_ 5.48, dd, *J* = 15.5, 7.5 Hz) and H-23 (*δ*_H_ 5.39, dd, *J* = 15.5, 8.0 Hz), together with the correlations of H_2_-26 with C-24/C-27, H_3_-21 with C-20/C-22, and H_3_-28 with C-23/C-25 in the HMBC spectrum, illustrated the presence of a Δ^22*E*^-24-methyl-26-oxygenated side chain.

Comparing with published data for scoparioside C [24], the 1D NMR data and the correlations of signals in the ^1^H-^1^H COSY and HMBC spectra confirmed the 3-*O*-methyl-4-*O*-sulfated-xylopyranosyl moiety in **4** (Figure 1), which was also indicated by the fragment ion peak at *m*/*z* 495 [M − Na − 226]^−^ in the tandem mass spectrum (Figure S29). The position of the xylose was determined by an HMBC correlation of H-1′ to C-26. The coupling constant of the anomeric proton (*J* = 7.5 Hz) indicated the sugar moiety was β-oriented. The NOESY correlations (Figure 2) and comparison with scoparioside C [24] supported that the sugar moiety of **4** had a β-D-configuration. The NOESY correlations of H_3_-18 with OH-15/OH-16/OH-8, H_3_-19 with H-6/OH-8, H-5 with H-3, and H-14 with OH-7, revealed that the configurations of OH-3/OH-8/OH-15/OH-16 were β-oriented, and that OH-6/OH-7 were α-oriented. Accordingly, the structure of **4** was established as a sodium salt of (22*E*)-26-*O*-(3-*O*-methyl-4-*O*-sulfated-β-D-xylopyranosyl)-24-methyl-5α-cholestane-3β,6α,7α,8β,15β,16β,26-heptol.

Pectiniferoside E (**5**) was an isomer of **4**, with HR-ESI-MS signals at *m*/*z* 721.3470 [M − Na]^−^ (calcd for C_34_H_57_O_14_S^−^, 721.3475) and *m*/*z* 767.3279 [M + Na]^+^ (calcd for C_34_H_57_O_14_SNa_2_^+^, 767.3259), corresponding to a molecular formula of C_34_H_57_O_14_SNa. The 1D NMR spectral data of **5** (Table 3) were very similar to the chemical shifts in C-1 to C-28 of **4**, with the only difference in the monosaccharide residue. The obvious downfield shift in C-2′ (*δ*_c_ 80.2, Δ*δ*_c_ + 6.1) and upfield shifts in C-1′ (*δ*_c_ 102.4, Δ*δ*_c_ − 2.6) and C-3′ (*δ*_c_ 74.3, Δ*δ*_c_ − 10.8) of **5** compared to **4** implied that the sulfate ester group was linked at C-2′ instead of at C-4′ as in **4**. The correlation of H_3_-OMe with C-4′ in the HMBC spectrum (Figure 2) confirmed the methoxy group position at C-4′. The sugar signals of **5** were highly consistent with those of **2**, supporting the notion that compound **5** possessed a 4-*O*-methyl-2-*O*-sulfated-β-D-xylopyranosyl moiety. Therefore, compound **5** was identified as a sodium salt of (22*E*)-26-*O*-(4-*O*-methyl-2-*O*-sulfated-β-D-xylopyranosyl)-24-methyl-5α-cholestane-3β,6α,7α,8β,15β,16β,26-heptol.

Pectiniferoside F (**6**) was isolated as a colorless gum; its formula, C_35_H_59_O_14_SNa with six degrees of unsaturation, was established by HR-ESI-MS signals at *m*/*z* 735.3617 [M − Na]^−^ (calcd for C_35_H_59_O_14_S^−^, 735.3631) and *m*/*z* 781.3448 [M + Na]^+^ (calcd for C_35_H_59_O_14_SNa_2_^+^, 781.3415). The presence of a fragment ion at *m*/*z* 96.9 revealed a sulfate ester group in **6**. The ^1^H NMR data (Table 3) and HSQC spectrum revealed two angular methyls for CH_3_-18 and CH_3_-19, three doublet methyls for CH_3_-21, CH_3_-26, and CH_3_-27; the ^13^C NMR and DEPT spectra showed resonances for four oxymethine carbons, one oxygenated methylene, one oxygenated tertiary carbon, and two *sp*^2^ methines. Moreover, one anomeric carbon, four oxymethine carbons, and one methylene closing out the spin system for a hexose sugar were also observed. The correlations shown in both ^1^H-^1^H COSY and HMBC spectra (Figure 2) confirmed that five hydroxy groups were located at C-3, C-6, C-7, C-8, and C-15, respectively. The *J*_22,23_ = 15.5 Hz and HMBC correlations illustrated the presence of Δ^22*E*^-24-methyl-28-oxygenated side chain.

The NOESY correlations (Figure 3) of H_3_-18 to OH-15/OH-8, H_3_-19 to H-6/OH -8, H-5 to H-3, and H-14 to OH-7 revealed the presence of a 3β,6α,7α,8,15β-pentahydroxy tetracyclic steroidal nucleus. The 1D and 2D NMR data supported the 3-*O*-methyl-6-*O*-sulfated-glucosyl group in **6**, which was also supported by the fragment ion peak at *m*/*z* 479 [M− Na − 256]^−^ (Figure S46). Furthermore, the ^13^C NMR signals for C-6′ shifted downfield to *δ*_C_ 68.4 (*δ*_C_ 62.5 in 3-*O*-methyl-glucosyl group) [25], while the signals for C-5′ shifted upfield to *δ*_C_ 75.7 (*δ*_C_ 77.3 in 3-*O*-methyl-glucosyl group), indicating that the sulfate group was located at C-6′. The β-oriented configuration of glucose was assigned based on the coupling constant of the anomeric proton (*J* = 7.5 Hz), and the position of glucose was determined by the HMBC correlation of H-1′ with C-28. The β-D-configuration of the sugar moiety was confirmed through acid hydrolysis, GC-MS analysis, and comparison to the corresponding derivatives of a standard monosaccharide. Thus, the structure of **6** was established as a sodium salt of (22*E*)-28-*O*-(3-*O*-methyl-6-*O*-sulfated-β-D-glucosyl)-24-methyl-5α-cholestane-3β,6α,7α,8β,15β,28-hexaol.

Pectiniferoside G (**7**) was isolated as a white, amorphous powder. Its molecular formula was established as C_35_H_60_O_11_ by the HR-ESI-MS signals at *m*/*z* 655.4053 [M − H]^−^ (calcd for C_35_H_59_O_11_^−^, 655.4063) and *m*/*z* 657.4222 [M + H]^+^ (calcd for C_35_H_61_O_11_^+^, 657.4208). The ^1^H NMR spectrum of **7** (Table 3) demonstrated that it contained the same aglycone as **6,** but a different sugar moiety. The tandem mass spectrum gave a fragment ion with *m*/*z* 479 [M− H−176]^−^ (Figure S56), along with the presence of a methoxy singlet at *δ*_H_ 3.63 in the ^1^H NMR spectrum, which confirmed that **7** contained a methoxylated hexose moiety. A detailed comparison the chemical shifts in the sugar moiety between **7** and antarcticoside O [25] in 1D NMR revealed the 3-*O*-methyl-β-D-glucosyl moiety in **7.** Therefore, the structure of **7** was established as (22*E*)-28-*O*-(3-*O*-methyl-β-D-glucosyl)-24-methyl-5α-cholestane-3β,6α,7α,8β,15β,28-hexaol.

Pectiniferoside H (**8**) was obtained as a colorless gum, and its HR-ESI-MS signal at *m*/*z* 707.3334 [M − Na]^−^ (calcd for C_33_H_55_O_14_S^−^, 707.3318) and *m*/*z* 753.3111 [M + Na]^+^ (calcd for C_33_H_55_O_14_SNa_2_^+^, 753.3102) established the molecular formula C_33_H_55_O_14_SNa with six degrees of unsaturation. A fragment ion peak at *m*/*z* 96.9 (Figure S64) indicated a sulfate ester group in **8**. One-dimensional NMR spectral data analysis (Table 4) revealed that compounds **8** and **4** possessed identical nuclei with the same sugar moiety, differing only in the side chain. The ^1^H NMR spectrum of **8** showed signals of two doublet methyls, one oxygenated methylene, and two *sp*^2^ methines. The correlations of protons of C-21 to C-26 and C-28 to C-24 in the ^1^H-^1^H COSY spectrum (Figure 2), and the HMBC correlations of H-23 (*δ*_H_ 5.31, dd, *J* = 15.5, 8.5 Hz) with C-28/C-24, H-21 with C-20/C-22, and H-26 with C-24, suggested a Δ^22*E*^-27-nor-24-methyl-26-oxygenated side chain in **8** similar to pectinioside I [26]. The correlation of H-1′ (*δ*_H_ 4.20, d, *J* = 7.5 Hz) to C-26 confirmed that the sugar moiety was linked at C-26. Thus, the structure of **8** was established as a sodium salt of (22*E*)-26-*O*-(3-*O*-methyl-4-*O*-sulfated-β-D-xylopyranosyl)-27-nor-24-methyl-5α-cholestane-3β,6α,7α,8β,15β,16β,26-heptol.

Pectiniferoside I (**9**) was purified as a colorless gum, and the HR-ESI-MS showed molecular ion peaks at *m*/*z* 721.3488 [M − Na]^−^ (calcd for C_34_H_57_O_14_S^−^, 721.3475) and *m*/*z* 767.3259 [M + Na]^+^ (calcd for C_34_H_57_O_14_SNa_2_^+^, 767.3259), establishing the molecular formula C_34_H_57_O_14_SNa with six degrees of unsaturation. The ^1^H NMR spectroscopic data (Table 4) of **9** and **4** were nearly identical except for the olefinic proton signals. The HMBC correlations (Figure 2) from H_2_-28 (*δ*_H_ 4.81, s; *δ*_H_ 4.76, s) to C-23 and C-25, and from H_3_-27 (*δ*_H_ 1.09, d, *J* = 6.5 Hz) to C-24 and C-26, indicated the presence of a vinylic olefin connecting C-24 and C-28, whereas in **4,** the olefin was 1,2-disubstituted. As a result, the structure of **9** was determined as a sodium salt of 26-*O*-(3-*O*-methyl-4-*O*-sulfated-β-D-xylopyranosyl)-24-methyl-5α-cholestane-24(28)-ene-3β,6α,7α,8β,15β,16β,26-heptol.

Pectiniferoside K (**10**) was isolated as a colorless gum; the molecular formula of compound **10** (C_32_H_55_O_12_SNa) was deduced from HR-ESI-MS at *m*/*z* 663.3430 [M − Na]^−^ (calcd for C_32_H_55_O_12_S^−^, 663.3420) and *m*/*z* 709.3224 [M + Na]^+^ (calcd for C_32_H_55_O_12_SNa_2_^+^, 709.3204). The signals of **10** in the ^1^H NMR and ^13^C NMR spectra (Table 4) closely resembled those of the known compound asterosaponin P_1_ [27,28], isolated from *P. pectinifera* and *Patiria pectinifera*, with the only difference being the absence of a methoxy group in **10**. The 1D NMR resonances for the sugar residue matched the 5-*O*-sulfated-α-L-arabinofuranosyl moiety in **3**, while the correlation between the anomeric proton (H-1′, *δ*_H_ 4.91) and C-24 (*δ*_C_ 85.1) confirmed the position of sugar at C-24. NOESY correlations (Figure 3) from H-5 to H-3, H_3_-19 to H-6 and OH-8, and H_3_-18 to H-15 confirmed that the compound **10** possessed a 5α-cholestane-3β,6α,8,15α,24-pentol nucleus. Based on these data, the structure of **10** was determined as a sodium salt of 24-*O*-(5-*O*-sulfated-α-L-arabinofuranosyl)-5α-cholestane-3β,6α,8β,15α,24-pentol.

Overall, ten glycosides of polyhydroxy steroids were isolated from *P. pectinifera*. The sugars linked to the steroid backbone included xylopyranose, arabinofuranose, and glucose. The chemical diversity in *P. pectinifera* is further enhanced by variations in the positions of methoxy and sulfate groups. Notably, the sulfation at C-2 of 4-O-methylxylose in **2** and **5**, as well as at C-6 of 3-O-methylglucose in **6**, represent the first report of such modifications in the study of steroid glycoconjugates from sea stars.

Compounds **1**–**10** were exposed to marine medaka embryos to investigate their toxic effects, as assessed by changes in embryonic phenotypes, such as mortality rate, malformation rate, and heart rate. However, none of these compounds showed toxicity to embryos at 100 μM. The absence of embryotoxicity in these compounds may be attributed to their low bioavailability, limited ability to reach or interact with critical developmental targets, or insufficient potency to disrupt key embryonic processes at the tested concentrations. Compounds **1**–**6** and **10** were also tested for their cytotoxicity and anti-inflammatory effects. The tested compounds showed no cytotoxicity against five human cancer cell lines (HL-60, A549, HepG2, MDA-MB-231, and SW480) at a concentration of 40 μM. Additionally, they did not exhibit anti-inflammatory activities in LPS-stimulated RAW264.7 cells at a concentration of 50 μM (Table S1). This lack of activity suggests that the presence of sulfated and methylated groups alone may not be sufficient to confer anti-inflammatory properties, and other structural features or synergistic interactions may play a more significant role in determining this bioactivity. Recently, three new monosulfated polyhydroxysteroid glycosides, spiculiferosides A–C, were isolated from the sea star *Henricia leviuscula spiculifera*. Among them, spiculiferoside C demonstrated significant antiproliferative activity by inhibiting proliferation and colony formation of colorectal carcinoma HCT 116 cells through induction of cell cycle arrest at the G2/M phase [29].

Previous studies have demonstrated that some saponins are responsible for chemical defense and toxicity [30,31,32]. The metabolome analysis of steroid metabolites from *P. pectinifera* under conditions of active feeding and stresses revealed a decrease in astersaponins concentrations, accompanied by an increase in polyhydroxy steroid glycosides. The observed elevation in polyhydroxy steroid glycosides is likely associated with their neuritogenic activities [33]. In our study, various compounds were predicted through metabolomic analysis. For example, the measured *m*/*z* 721.3845 [M − Na]^−^ in the metabolomic analysis may correspond to compounds **1** or **2**, while the measured *m*/*z* 707.3679 [M − Na]^−^ likely indicates compound **3**. Similarly, the measured *m*/*z* 735.3651 [M − Na]^−^ suggests the presence of compound **6**. Additionally, the significant enrichment of polyhydroxy steroid glycosides demonstrates that these compounds may play a role in enhancing neuroprotection as part of the stress response. Conversely, asterosaponins, which are released into seawater, may exert chemical defense functions through hemolytic activity, potentially deterring predators or pathogens [13,34]. Simultaneously, pharmacological research has shown that asterosaponins possess antitumor activity [35,36], highlighting the need to investigate their pharmacological potential in future research.

## 3. Materials and Methods

### 3.1. General Experimental Procedures

^1^H and ^13^C NMR spectra were acquired via a Bruker AVANCE IIITM 500 NMR spectrometer (500 MHz for ^1^H NMR and 125 MHz for ^13^C NMR spectra) using tetramethylsilane (TMS) as an internal standard for chemical shifts in CD_3_OD and (CD_3_)_2_SO. Chemical shifts (*δ*) were expressed in ppm and coupling constants were reported in Hz. High-resolution electrospray ionization mass spectrometry (HR-ESI-MS) data were performed on the Waters ACOUQUITY UPLC system coupled to a quadrupole time-of-flight mass spectrometer (Waters Xevo^®^ G2-XS Q-Tof, Waters, Milford, MA, USA). Optical rotations were obtained with a Rudolph Autopol VI polarimeter. GC-MS analysis was recorded on an Agilent 7890B-7000D apparatus (Agilent, Santa Clara, CA, USA) with DB-5 capillary column (0.25 mm × 30 m, 0.25 μm). A Waters Prep 150, equipped with a diode array detector using the XBridge^®^ C18 OBD^TM^ column (5.0 μm, 19 mm × 250 mm) and column (5.0 μm, 10 mm × 250 mm), was used to purify compounds. A vacuum centrifugal concentrator (CV200, Jim Instrument Co., Ltd., Beijing, China) was used to remove organic reagents.

Column chromatography was performed using silica gel (200–300 mesh, Marine Chemical, Inc., Qingdao, China), ODS (AAG12S50, YMC Co., Ltd., Osaka, Japan), and Sephadex LH-20 (Sigma, Perrysburg, OH, USA). The compounds were monitored by TLC (GF254, Marine Chemical Ltd., Qingdao, China) and by heating silica gel plates sprayed with 5% H_2_SO_4_ and 0.5% *p*-anisaldehyde in methanol (MeOH). MeOH, ethanol (EtOH), dichloromethane (CH_2_Cl_2_), petroleum ether, ethyl acetate, and *n*-butanol (*n*-BuOH) were of analytical grade, and all of them were purchased from Sinopharm Chemical Reagent Co., Ltd., Beijing, China. The MeOH and acetonitrile (MeCN) used in HPLC were of the highest grade available (OCEANPAK, Goteborg, Sweden).

### 3.2. Animal Material

Specimens of *P. pectinifera* (order Spinulosa, family Asterinidae) were collected during a population outbreak in August 2021 from the junction of the Yellow Sea and the Bohai Sea near Changdao County. Species were identified by Dr. Quanchao Wang of the Yantai Institute of Coastal Zone Research, Chinese Academy of Sciences, China. A voucher specimen of *A. pectinfera* was deposited in the Key Laboratory of Coastal Zone Biology and Biological Resources Utilization (Voucher No. 2021-003).

### 3.3. Extraction and Isolation

The air-dried and powdered animals (87 kg, dry weight) were extracted three times with ethanol at room temperature and then concentrated under a vacuum to yield a crude extract. The EtOH extract was suspended in water and further partitioned with petroleum ether, ethyl acetate, and *n*-BuOH (160 g). The *n*-BuOH fraction was subjected to XAD-2 resin (3 L) with distilled water, 30% MeOH/H_2_O, 50% MeOH/H_2_O, 70% MeOH/H_2_O, 90% MeOH/H_2_O, and MeOH, respectively. The 70% MeOH/H_2_O (35 g) fraction was chromatographed over an RP–C18 column with MeOH/H_2_O (40:60 → 100:0) to afford seven fractions (Fr. 1–Fr. 7). Fr. 2 (4.6 g) was isolated through a silica gel column and stepwise eluted with CH_2_Cl_2_/MeOH (8:1, 5:1, 3:1, and 1:1) to obtain four fractions (Fr. 2-1–Fr. 2-4). Fr. 2-2 (0.4 g) was separated by Sephadex LH-20 column (MeOH) and then purified on an XBridge^®^ C18 OBD^TM^ column (5 μm, 19 mm × 250 mm, 10 mL/min) eluted with MeOH/H_2_O (10:90 → 100:0 for 55 min) to afford **3** (6.9 mg, *t*_R_ = 43.7 min) and **10** (5.6 mg, *t*_R_ = 41.5 min). Fr. 3 (16.6 g) was fragmented by a silica gel column (CH_2_Cl_2_/MeOH 15:1, 8:1, 5:1, 3:1, and 1:1) and yielded fractions (Fr. 3-1–Fr. 3-6). Fr. 3-3 was further purified by preparative HPLC (55% MeOH/H_2_O, 10 mL/min) to afford **8** (2.9 mg, *t*_R_ = 20.0 min) and **9** (3.2 mg, *t*_R_ = 22.5 min). Fr.4 was also isolated by silica gel column to give Fr. 4-1–Fr. 4-3. Compound **1** (10.6 mg, *t*_R_ = 11.5 min) was yielded from Fr. 4-1 by preparative HPLC (63% MeOH/H_2_O, 10 mL/min). Fr. 4-2 was further purified by Sephadex LH-20 column (MeOH) followed by semi-preparative HPLC (55% MeOH/H_2_O, 3 mL/min) to afford **4** (3.8 mg, *t*_R_ = 18.5 min) and **5** (5.8 mg, *t*_R_ = 20.0 min). Fr. 4-3 was purified by HPLC with a gradient elution of MeCN/H_2_O (10% → 100%, 10 mL/min) to afford compounds **2** (10.0 mg, *t*_R_ = 24.2 min), **6** (14.0 mg, *t*_R_ = 20.5 min), and **7** (4.0 mg, *t*_R_ = 26.5 min).

Pectiniferoside A (**1**): colorless gum; ${\left[\alpha \right]}_{D}^{25}$ 13.00 (*c* 0.120, MeOH); negative HR-ESI-MS *m*/*z* 721.3866 [M − Na]^−^ (calcd for C_35_H_61_O_13_S^−^, 721.3838, Δ = 3.9 ppm); positive HR-ESI-MS *m*/*z* 767.3646 [M + Na]^+^ (calcd for C_35_H_61_O_13_SNa_2_^+^, 767.3623, Δ = 3.0 ppm). ^13^C and ^1^H NMR were shown in Table 1 and Table 2. ESIMS/MS of the [M − Na]^−^ ion at *m*/*z* 721: 495 [M − Na − C_6_H_10_O_7_S]^−^, 96.9 [HSO_4_]^−^.

Pectiniferoside B (**2**): colorless gum; ${\left[\alpha \right]}_{D}^{25}$ 19.50 (*c* 0.200, MeOH); negative HR-ESI-MS *m*/*z* 721.3828 [M − Na]^−^ (calcd for C_35_H_61_O_13_S^−^, 721.3838, Δ = −1.4 ppm); positive HR-ESI-MS *m*/*z* 767.3630 [M + Na]^+^ (calcd for C_35_H_61_O_13_SNa_2_^+^, 767.3623, Δ = 0.9 ppm). ^13^C and ^1^H NMR were shown in Table 1. ESIMS/MS of the [M − Na]^−^ ion at *m*/*z* 721: 495 [M − Na − C_6_H_10_O_7_S]^−^, 96.9 [HSO_4_]^−^.

Pectiniferoside C (**3**): colorless gum; ${\left[\alpha \right]}_{D}^{20}$ 8.54 (*c* 0.110, MeOH); negative HR-ESI-MS *m*/*z* 707.3715 [M − Na]^−^ (calcd for C_34_H_59_O_13_S^−^, 707.3682, Δ = 4.7 ppm); positive HR-ESI-MS *m*/*z* 753.3473 [M + Na]^+^ (calcd for C_34_H_59_O_13_SNa_2_^+^, 753.3466, Δ = 0.9 ppm). ^13^C and ^1^H NMR were shown in Table 1. ESIMS/MS of the [M − Na]^−^ ion at *m*/*z* 707: 495 [M − Na − C_5_H_8_O_7_S]^−^, 96.9 [HSO_4_]^−^.

Pectiniferoside D (**4**): colorless gum; ${\left[\alpha \right]}_{D}^{25}$ 6.60 (*c* 0.100, MeOH); negative HR-ESI-MS *m*/*z* 721.3481 [M − Na]^−^ (calcd for C_34_H_57_O_14_S^−^, 721.3475, Δ = 0.8 ppm); positive HR-ESI-MS *m*/*z* 767.3275 [M + Na]^+^ (calcd for C_34_H_57_O_14_SNa_2_^+^, 767.3259, Δ = 2.1 ppm). ^13^C and ^1^H NMR were shown in Table 2 and Table 3. ESIMS/MS of the [M − Na]^−^ ion at *m*/*z* 721: 495 [M − Na − C_6_H_10_O_7_S]^−^, 96.9 [HSO_4_]^−^.

Pectiniferoside E (**5**): colorless gum; ${\left[\alpha \right]}_{D}^{25}$ 6.77 (*c* 0.130, MeOH); negative HR-ESI-MS *m*/*z* 721.3469 [M − Na]^−^ (calcd for C_34_H_57_O_14_S^−^, 721.3475, Δ = −0.8 ppm); positive HR-ESI-MS *m*/*z* 767.3279 [M + Na]^+^ (calcd for C_34_H_57_O_14_SNa_2_^+^, 767.3259, Δ = 2.6 ppm). ^13^C and ^1^H NMR were shown in Table 3. ESIMS/MS of the [M − Na]^−^ ion at *m*/*z* 721: 495 [M − Na − C_6_H_10_O_7_S]^−^, 96.9 [HSO_4_]^−^.

Pectiniferoside F (**6**): colorless gum; ${\left[\alpha \right]}_{D}^{25}$ –4.55 (*c* 0.110, MeOH); negative HR-ESI-MS *m*/*z* 735.3617 [M − Na]^−^ (calcd for C_35_H_59_O_14_S^−^, 735.3631, Δ = −1.9 ppm); positive HR-ESI-MS *m*/*z* 781.3448 [M + Na]^+^ (calcd for C_35_H_59_O_14_SNa_2_^+^, 781.3415, Δ = 4.2 ppm). ^13^C and ^1^H NMR were shown in Table 2 and Table 3. ESIMS/MS of the [M − Na]^−^ ion at *m*/*z* 735: 479 [M − Na − C_7_H_12_O_8_S]^−^, 96.9 [HSO_4_]^−^.

Pectiniferoside G (**7**): white amorphous powder; ${\left[\alpha \right]}_{D}^{25}$ −12.73 (*c* 0.110, MeOH); negative HR-ESI-MS *m*/*z* 655.4053 [M − H]^−^ (calcd for C_35_H_59_O_11_^−^, 655.4063, Δ = −1.5 ppm); positive HR-ESI-MS *m*/*z* 657.4222 [M + H]^+^ (calcd for C_35_H_61_O_11_^+^, 657.4208, Δ = 2.1 ppm). ^13^C and ^1^H NMR were shown in Table 3. ESIMS/MS of the [M − H]^−^ ion at *m*/*z* 655: 479 [M − H − C_7_H_12_O_5_]^−^.

Pectiniferoside H (**8**): colorless gum; ${\left[\alpha \right]}_{D}^{25}$ 10.44 (*c* 0.090, MeOH); negative HR-ESI-MS *m*/*z* 707.3344 [M − Na]^−^ (calcd for C_33_H_55_O_14_S^−^, 707.3318, Δ = 3.7 ppm); positive HR-ESI-MS *m*/*z* 753.3111 [M + Na]^+^ (calcd for C_33_H_55_O_14_SNa_2_^+^, 753.3102, Δ = 1.2 ppm). ^13^C and ^1^H NMR were shown in Table 4. ESIMS/MS of the [M − Na]^−^ ion at *m*/*z* 707: 481 [M − Na − C_6_H_10_O_7_S]^−^, 96.9 [HSO_4_]^−^.

Pectiniferoside I (**9**): colorless gum; ${\left[\alpha \right]}_{D}^{25}$ 7.50 (*c* 0.120, MeOH); negative HR-ESI-MS *m*/*z* 721.3488 [M − Na]^−^ (calcd for C_34_H_57_O_14_S^−^, 721.3475, Δ = 1.8 ppm); positive HR-ESI-MS *m*/*z* 767.3275 [M + Na]^+^ (calcd for C_34_H_57_O_14_SNa_2_^+^, 767.3259, Δ = 2.1 ppm). ^13^C and ^1^H NMR were shown in Table 4. ESIMS/MS of the [M − Na]^−^ ion at *m*/*z* 721: 495 [M − Na − C_6_H_10_O_7_S]^−^, 96.9 [HSO_4_]^−^.

Pectiniferoside J (**10**): colorless gum; ${\left[\alpha \right]}_{D}^{25}$ 8.16 (*c* 0.120, MeOH); negative HR-ESI-MS *m*/*z* 663.3430 [M − Na]^−^ (calcd for C_32_H_55_O_12_S^−^, 663.3420, Δ = 1.5 ppm); positive HR-ESI-MS *m*/*z* 709.3224 [M + Na]^+^ (calcd for C_32_H_55_O_12_SNa_2_^+^, 709.3204, Δ = 2.8 ppm). ^13^C and ^1^H NMR were shown in Table 2 and Table 4. ESIMS/MS of the [M − Na]^−^ ion at *m*/*z* 663: 451 [M − Na − C_5_H_8_O_7_S]^−^, 96.9 [HSO_4_]^−^.

### 3.4. Hydrolysis and Identification of the Sugar Moieties in ***1***, ***2***, ***3***, and ***6***

The experiments were conducted according to the published methods [37] with minor modifications. Briefly, **3** and **6** (3.0 mg of each) were dissolved in 2 M TFA-H2O (5 mL) and stirred for 8 h at 100 °C individually and then extracted by CH_2_Cl_2_ (5 mL × 3), and the aqueous layer was evaporated under reduced pressure to yield the monosaccharide. The residue was dissolved in anhydrous pyridine (1.0 mL) containing NH_2_OH∙HCl (2.0 mg) and stirred for 30 min at 90 °C. Next, the Ac_2_O (0.8 mL) was added to the reaction mixtures and stirred for 1 h at 90 °C. The reaction mixtures were evaporated under reduced pressure and dissolved in acetone. The resulting aldononitrile peracetates were analyzed by GC-MS: the carrier gas was N_2_; the constant flow rate was 3 mL/min; the GC oven was set at 150 °C to 300 °C at 15 °C/min; the final temperature was maintained for 10 min; the injector temperature was 300 °C; and the detector temperature was 230 °C. The absolute configurations of carbohydrates were determined by comparing the retention times with standard aldononitrile peracetates prepared from authentic sugars.

Compounds **1** and **2** (3.0 mg of each) were individually dissolved in MeOH (5 mL) and then refluxed with 0.5 M HCl (3 mL) at 70 °C for 4 h. Each reaction mixture was diluted with H_2_O and extracted with CH_2_Cl_2_ (5 mL × 3). The aqueous layer was then neutralized with AgCO_3_, and the precipitate was filtered to give the monosaccharide. The D-configurations of sugar moieties for **1** with optical rotation ${\left[\alpha \right]}_{D}^{23}$ 44.25 (0.08, H_2_O) and **2** with optical rotation ${\left[\alpha \right]}_{D}^{23}$ 73.2 (0.05, H_2_O) were proposed by comparing specific rotation with analogs [23].

### 3.5. Acute Toxicity Tests on Embryos of Marine Medaka (Oryzias melastigma)

Marine medaka (*O. melastigma*) have been cultured in the laboratory for 9 months. The fish were kept in filtered seawater (FS, salinity 30‰) at 27–28 °C with a cycle of 14 h light/10 h dark and were fed with brine shrimp three times daily. Embryos were collected within 8–10 h post-fertilization after spawning to ensure the synchronization of the eggs for the subsequent bioassays. Healthy embryos were selected using an Olympus CKX53 (Olympus, Waltham, MA, USA) inverted microscope and randomly assigned to 12-well plates for experiments containing 10 embryos/well in a 2 mL exposure solution for each group. Groups included blank control (BC) = FS, negative control (NC) = FS plus cosolvent (0.1% DMSO and 0.5% ethanol), treat = FS plus cosolvent, and test compounds (**1**–**10**). The experiment consisted of four replicates. During the embryotoxicity assays, marine medaka embryos were maintained under controlled conditions to ensure reproducibility. The salinity was kept at approximately 30 parts per thousand (ppt), and the temperature was maintained at 28 ± 1 °C. These parameters were carefully monitored throughout the experiments to replicate the optimal natural habitat of marine medaka and to provide consistent conditions for evaluating embryotoxicity. The exposure solution was changed daily. Every 24 h, embryos were examined under a microscope to count the number of deaths and deformities (24–96 h). Three embryos/well were randomly selected and their heart beats per minute were recorded (48–96 h).

### 3.6. Cytotoxicity Assay

Cell viability was determined by the MTS method [38]. Five cancer cells, including human leukemia HL60, liver cancer HepG2, breast cancer MDA-MB-231, lung cancer A549, and colon cancer SW480, were selected to test the cytotoxicity of **1**–**6** and **10**. The concentration for preliminary screening was 40 μM, with cisplatin (DDP) and taxol as positive controls.

### 3.7. Anti-Inflammatory Activity Assay

The RAW 264.7 cell line induced by LPS [39] was used to evaluate the anti-inflammatory activity of **1**–**6** and **10**. The test compounds at 50 μM were used for preliminary screening, with the positive control being N^G^-Methyl-L-arginine acetate salt (L-NMMA). The nitric oxide (NO) production was measured with the Griess reagent. The inhibition rate of NO production = (OD_control_ − OD_test_)/OD_control_ at 570 nm.

### 3.8. Statistical Analysis

The data were presented as the mean ± standard error of the mean (SEM) and tested for normal distribution (Shapiro–Wilk test) and homogeneity of variances (Levene’ test). Statistical significance of differences was assessed using Independent-Samples *T*-Test for normally distributed data, otherwise the Mann–Whitney U Test was applied (SPSS Inc., Chicago, IL, USA). Statistical significance was determined at *p* < 0.05.

## 4. Conclusions

In summary, a total of ten new glycosides of polyhydroxy steroids, pectiniferosides A–J, were isolated from sea star *P. pectinifera*, which has been a widespread species in the Yellow Sea and the Bohai Sea of China. After utilizing marine medaka embryos to evaluate the toxicological effects of isolated glycosides, the results showed that none of these compounds exhibited embryotoxicity at 100 μM. In addition, these compounds did not exhibit significant cytotoxicity and anti-inflammatory activity. Overall, studying the constituents of *P. pectinifera* could provide a potential foundation for ecotoxicology and drug development. Future studies could focus on evaluating additional biological activities, such as antiviral or neuroprotective effects, and exploring the ecological roles of these compounds in marine ecosystems. Furthermore, detailed structure-activity relationship studies and the optimization of these compounds could be conducted to enhance their potential for drug development.

## Figures and Tables

**Table 1:** The ^1^H NMR and ^13^C NMR data of compounds **1**–**3** in CD_3_OD (*δ* in ppm, *J* in Hz).

No.	1		2		3	
	*δ*_H_	*δ*_C_, Type	*δ*_H_	*δ*_C_, Type	*δ*_H_	*δ*_C_, Type
1	1.73, m; 0.97, m	39.5, CH_2_	1.73, m; 0.98, m	39.5, CH_2_	1.73, m; 0.97, m	39.5, CH_2_
2	1.73, m; 1.45, m	31.5, CH_2_	1.71, m; 1.45, m	31.4, CH_2_	1.73, m; 1.63, m	31.4, CH_2_
3	3.47, m	72.2, CH	3.49, m	72.1, CH	3.48, m	72.1, CH
4	2.17, m; 1.21, m	32.3, CH_2_	2.18, m; 1.20, m	32.3, CH_2_	2.18, m; 1.20, m	32.3, CH_2_
5	1.02, m	53.6, CH	1.03, m	53.6, CH	1.03, m	53.5, CH
6	3.63, overlap	67.7, CH	3.63, td (11.0, 4.0)	67.6, CH	3.63, td (11.0, 4.0)	67.6, CH
7	2.41, dd (13.5, 4.0); 1.35, dd (13.5, 11.5)	50.1, CH_2_	2.41, dd (13.5, 4.0); 1.35, dd (13.5, 11.0)	50.0, CH_2_	2.41, dd (13.5, 4.0); 1.35, dd (13.5, 11.0)	50.0, CH_2_
8		75.8, C		75.8, C		75.8, C
9	0.87, m	57.3, CH	0.87, m	57.3, CH	0.87, m	57.3, CH
10		37.8, C		37.8, C		37.8, C
11	1.71, m; 1.49, m	19.4, CH2	1.71, m; 1.49, m	19.4, CH_2_	1.70, m; 1.49, m	19.4, CH_2_
12	1.95, m; 1.20, m	43.1, CH2	1.95, m; 1.20, m	43.1, CH_2_	1.95, m; 1.19, m	43.1, CH_2_
13		45.3, C		45.2, C		45.2, C
14	1.13, d (11.0)	64.4, CH	1.14, d (11.0)	64.3, CH	1.14, d (11.0)	64.3, CH
15	4.06, dd (11.0, 2.5)	80.7, CH	4.07, overlap	80.7, CH	4.06, overlap	80.8, CH
16	4.03, dd (7.5, 2.5)	82.9, CH	4.02, dd (7.0, 2.5)	82.9, CH	4.01, dd (7.5, 2.5)	83.0, CH
17	1.22, m	60.6, CH	1.23, m	60.5, CH	1.24, m	60.4, CH
18	1.12, s	17.0, CH3	1.11, s	16.9, CH_3_	1.12, s	16.9, CH_3_
19	1.02, s	14.2, CH_3_	1.02, s	14.2, CH_3_	1.02, s	14.2, CH_3_
20	1.81, m	31.3, CH	1.81, m	31.2, CH	1.83, m	31.1, CH
21	0.93, d (7.0)	18.5, CH_3_	0.93, d (6.5)	18.5, CH_3_	0.94, d (6.5)	18.6, CH_3_
22	1.62, m; 1.00, m	35.0, CH_2_	1.58, m; 1.03, m	34.8, CH_2_	1.59, m; 1.02, m	34.9, CH_2_
23	1.43, m; 1.09, m	28.8, CH_2_	1.41, m; 1.13, m	28.7, CH_2_	1.43, m; 1.13, m	28.7, CH_2_
24	1.27, m	42.3, CH	1.29, m	42.0, CH	1.22, m	42.5, CH
25	1.73, m	30.7, CH	1.74, m	30.5, CH	1.74, m	30.7, CH
26	0.87, d (7.0)	19.9, CH_3_	0.87, d (7.0)	20.1, CH_3_	0.86, d (7.0)	19.9, CH_3_
27	0.85, d (6.5)	19.1, CH_3_	0.84, d (6.5)	18.9, CH_3_	0.85, d (6.5)	19.1, CH_3_
28	1.44, m; 1.67, m	31.4, CH_2_	1.71, m; 1.45, m	31.4, CH_2_	1.63, m; 1.46, m	31.6, CH_2_
29	3.74, m; 3.51, m	68.9, CH_2_	3.81, m; 3.54, m	69.6, CH_2_	3.75, m; 3.43, m	68.2, CH_2_
1′	4.70, d (3.5)	101.6, CH	4.47, d (6.5)	102.3, CH	4.85, d (2.0)	109.4, CH
2′	4.23, dd (5.0, 3.5)	77.1, CH	4.07, overlap	80.6, CH	3.94, brd	83.5, CH
3′	3.39, t (5.0)	82.6, CH	3.75, t (7.5)	74.8, CH	3.89, dd (6.0, 4.0)	78.9, CH
4′	3.62, m	68.8, CH	3.27, m	80.1, CH	4.06, overlap	82.7, CH
5′	3.94, dd (12.0, 4.0); 3.35, t (6.0)	63.8, CH_2_	4.05, overlap; 3.27, overlap	63.1, CH_2_	4.18, dd (10.0, 3.0); 4.09, overlap	68.6, CH2
OMe	3.55, s	59.5, CH_3_	3.47, s	58.8, CH_3_		

Measured at 500/125 MHz.

**Table 2:** The ^1^H NMR and ^13^C NMR data of compounds **1**, **4**, **6**, and **10** in DMSO-*d*_6_ (*δ* in ppm, *J* in Hz).

No.	1		4		6		10	
	*δ*_H_	*δ*_C_	*δ*_H_	*δ*_C_	*δ*_H_	*δ*_C_	*δ*_H_	δ_C_
1	1.57, m; 0.81, m	38.2	1.54, m; 0.85, m	38.0	1.55, m; 0.85, m	38.0	1.57, m; 0.84, m	38.3
2	1.57, m; 1.30, m	30.8	1.57, m; 1.29, m	30.8	1.57, m; 1.29, m	30.8	1.57, m; 1.30, m	30.8
3	3.26, m	69.9	3.28, m	69.9	3.27, m	70.0	3.27, m	69.9
4	2.05, m; 0.96, m	31.9	1.95, m; 0.99, m	31.5	1.94, m; 0.98, m	31.5	2.05, m; 0.96, m	31.8
5	0.80, m	52.3	1.43, m	42.6	1.42, m	42.5	0.82, m	52.3
6	3.39, overlap	65.0	3.61, m	67.0	3.60, m	67.0	3.38, m	65.0
7	2.28, dd (8.5, 4.0); 1.15, overlap	49.1	3.69, overlap	74.6	3.64, t (3.5)	74.4	2.26, dd (11.0, 4.0); 1.17, m	49.3
8		73.9		77.7		77.9		74.1
9	0.67, d (12.5)	55.8	1.04, m	48.3	1.03, m	48.2	0.68, d (11.5)	55.7
10		36.2		36.0		36.0		36.2
11	1.51, m; 1.34, m	18.1	1.65, m; 1.39, m	17.7	1.68, m; 1.41, m	18.0	1.56, m; 1.36, m	18.2
12	1.80, m; 1.07, m	41.8	1.80, m; 1.03, m	41.3	1.85, m; 1.08, m	41.3	1.83, m; 1.13, m	41.4
13		43.6		42.6		42.3		43.7
14	0.95, d (10.5)	63.3	1.29, d (5.0)	53.3	1.32, d (5.5)	54.7	1.11, overlap	65.7
15	3.90, m	78.8	4.35, dd (7.0, 5.0)	69.2	4.38, m	68.8	4.07, m	67.2
16	3.83, m	80.9	3.96, t (7.0)	71.2	2.07, m; 1.23, m	42.3	1.70, m; 1.63, m	40.7
17	1.07, m	58.7	0.85, m	62.0	0.95, m	55.7	1.22, m	54.2
18	1.03, s	16.5	1.19, s	17.2	1.22, s	16.0	0.87, s	15.1
19	0.90, s	13.7	0.88, s	13.4	0.88, s	13.4	0.90, s	13.8
20	1.73, m	29.3	2.51, m	32.3	2.09, m	39.6	1.22, m	34.6
21	0.84, d (6.5)	18.0	0.98, d (7.0)	19.3	0.97, d (6.5)	20.5	0.84, d (6.0)	18.4
22	1.50, m; 0.91, m	33.2	5.54, dd (15.5, 7.0)	135.3	5.25, dd (15.5, 8.5)	138.8	1.55, m; 0.84, m	31.3
23	1.32, m; 0.99, m	27.1	5.31, dd (15.5, 7.5)	131.8	5.17, dd (15.5, 8.5)	125.0	1.46, m; 1.15, m	27.3
24	1.67, m	28.9	2.14, m	37.6	2.08, m	47.6	3.20, m	81.9
25	1.13, m	40.6	1.64, m	38.0	1.89, m	26.8	1.76, m	30.1
26	0.81, d (6.0)	19.3	3.69, m; 3.07, m	72.2	0.84, d (7.0)	21.0	0.84, d (6.0)	17.9
27	0.80, d (6.0)	18.8	0.81, d (6.5)	13.6	0.77, d (7.0)	17.5	0.84, d (6.0)	17.8
28	1.50, m; 1.33, m	30.2	0.87, d (6.5)	16.1	3.70, m; 3.35, m	70.3		
29	3.57, m; 3.31, m	66.3						
1′	4.67, overlap	99.5	4.08, d (7.5)	103.8	4.12, d (8.0)	102.8	4.75, d (2.0)	107.5
2′	3.99, dd (4.0, 3.0)	74.4	3.15, overlap	72.2	3.02, m	72.9	3.76, td (4.5, 2.0)	82.4
3′	3.22, overlap	81.4	2.99, t (9.0)	83.6	2.93, t (8.5)	86.4	3.59, overlap	77.6
4′	3.57, m	66.0	3.94, m	74.0	3.11, m	69.5	3.85, m	81.0
5′	3.72, dd (11.0, 4.5); 3.22, overlap	62.6	4.04, overlap; 3.13, overlap	63.5	3.27, m	74.7	3.88, dd (11.5, 3.5); 3.71, dd (11.5, 6.0)	66.2
6′					4.03, m; 3.73, m	65.8		
OMe	3.36, s	57.6	3.44, s	59.1	3.48, s	59.9		
OH-3	4.42, d (4.5)		4.43, d (4.5)		4.42, d (4.5)		4.42, d (4.5)	
OH-6	4.02, d (6.5)		4.05, overlap		4.00, d (7.5)		4.02, d (6.5)	
OH-7			4.39, d (3.5)		4.30, d (3.5)			
OH-8	3.61, s		3.82, s		4.07, s		3.59, s	
OH-15	4.12, d (6.5)		5.35, d (4.5)		5.43, d (3.5)		4.07, overlap	
OH-16	4.48, d (4.5)		4.17, d (6.5)					

Measured at 500/125 MHz.

**Table 3:** The ^1^H NMR and ^13^C NMR data of compounds **4**–**7** in CD_3_OD (*δ* in ppm, *J* in Hz).

**No.**	**4**		**5**		**6**		**7**	
	***δ*_H_**	***δ*_C_, Type**	***δ*_H_**	***δ*_C_, Type**	***δ*_H_**	***δ*_C_, Type**	***δ*_H_**	***δ*_C_, Type**
1	1.69, m; 1.00, m	39.3, CH_2_	1.70, m; 1.01, m	39.2, CH_2_	1.70, m; 1.01, m	39.3, CH_2_	1.71, m; 1.00, m	39.3, CH_2_
2	1.72, m; 1.46, m	31.4, CH_2_	1.73, m; 1.48, m	31.5, CH_2_	1.73, m; 1.48, m	31.5, CH_2_	1.73, m; 1.48, m	31.5, CH_2_
3	3.51, m	72.3, CH	3.51, m	72.2, CH	3.50, m	72.2, CH	3.50, m	72.3, CH
4	2.09, m; 1.22, m	32.1, CH_2_	1.22, m; 2.08, m	32.1, CH_2_	2.08, m; 1.21, m	32.1, CH_2_	2.09, m; 1.21, m	32.1, CH_2_
5	1.57, td (11.5, 2.5)	44.3, CH	1.59, m	44.3, CH	1.57, td (13.0, 2.0)	44.3, CH	1.57, td (13.0, 2.0)	44.3, CH
6	3.83, overlap	69.5, CH	3.85, dd (11.5, 2.5)	69.5, CH	3.83, overlap	69.5, CH	3.82, overlap	69.4, CH
7	3.89, d (2.5)	76.5, CH	3.92, d (2.5)	76.7, CH	3.87, d (2.5)	76.3, CH	3.84, overlap	76.3, CH
8		79.3, C		79.2, C		79.5, C		79.5, C
9	1.14, m	50.2, CH	1.15, m	50.1, CH	1.14, m	50.1, CH	1.14, m	50.0, CH
10		37.6, C		37.6, C		37.6, C		37.6, C
11	1.80, m; 1.49, m	19.2, CH_2_	1.81, m; 1.49, m	19.1, CH_2_	1.83, m; 1.52, m	19.5, CH_2_	1.83, m; 1.52, m	19.5, CH_2_
12	1.92, m; 1.13, m	43.0, CH_2_	1.14, m; 1.92, m	43.0, CH_2_	1.94, m; 1.19, m	42.9, CH_2_	1.95, m; 1.18, m	43.0, CH_2_
13		44.4, C		44.4, C		44.1, C		44.1, C
14	1.41, d (5.0)	55.3, CH	1.40, d (5.5)	55.2, CH	1.43, d (5.5)	56.5, CH	1.43, d (5.5)	56.7, CH
15	4.49, dd (7.0, 5.5)	71.1, CH	4.46, dd (7.0, 5.5)	71.2, CH	4.50, m	71.1, CH	4.49, m	71.1, CH
16	4.15, t (7.0)	73.2, CH	4.16, t (7.0)	73.1, CH	2.26, m; 1.35, m	43.7, CH_2_	2.24, m; 1.36, m	43.6, CH_2_
17	0.98, m	63.6, CH	1.01, m	63.5, CH	1.03, m	57.6, CH	1.03, m	57.7, CH
18	1.28, s	17.8, CH_3_	1.28, s	17.9, CH_3_	1.30, s	16.6, CH_3_	1.30, s	16.6, CH_3_
19	0.99, s	13.9, CH_3_	0.99, s	13.9, CH_3_	0.99, s	13.9, CH_3_	0.99, s	13.9, CH_3_
20	2.61, m	34.5, CH	2.60, m	34.5, CH	2.16, m	41.3, CH	2.17, m	41.2, CH
21	1.04, d (7.0)	20.3, CH_3_	1.04, d (7.0)	20.3, CH_3_	1.03, d (6.5)	21.1, CH_3_	1.03, d (6.5)	21.1, CH_3_
22	5.48, dd (15.5, 7.5)	137.0, CH	5.51, dd (15.5, 8.0)	137.1, CH	5.22, m	140.3, CH	5.28, m	140.3, CH
23	5.39, dd (15.5, 8.0)	134.0, CH	5.39, dd (15.5, 8.5)	134.2, CH	5.22, m	127.6, CH	5.26, m	127.3, CH
24	2.09, m	40.3, CH	1.62, m	40.0, CH	2.08, m	50.1, CH	2.13, m	50.1, CH
25	1.66, m	39.8, CH	2.18, m	40.0, CH	1.87, m	29.1, CH	1.89, m	29.1, CH
26	3.85, m; 3.25, dd (9.5, 7.0)	74.3, CH_2_	3.77, m; 3.33, m	74.3, CH_2_	0.91, d (6.5)	21.5, CH_3_	0.90, d (6.5)	21.5, CH_3_
27	0.92, d (6.5)	14.9, CH_3_	0.95, d (7.0)	15.0, CH_3_	0.84, d (7.0)	18.8, CH_3_	0.84, d (7.0)	18.5, CH_3_
28	0.96, d (7.0)	17.6, CH3	0.95, d (6.5)	17.7, CH_3_	3.85, m; 3.51, m	72.9, CH_2_	3.90, dd (9.0, 7.0); 3.50, m	72.7, CH_2_
1′	4.20, d (7.5)	105.0, CH	4.44, d (6.0)	102.4, CH	4.24, d (7.5)	104.3, CH	4.24, d (8.0)	104.4, CH
2′	3.28, dd (9.0, 7.5)	74.1, CH	4.08, dd (7.5, 6.0)	80.2, CH	3.22, dd (9.5, 7.5)	74.8, CH	3.23, dd (9.0, 8.0)	75.0, CH
3′	3.18, t (9.0)	85.1, CH	3.78, overlap	74.3, CH	3.09, t (9.5)	87.7, CH	3.07, t (9.0)	88.0, CH
4′	4.24, m	77.2, CH	3.27, m	80.0, CH	3.39, t (9.5)	71.1, CH	3.35, t (9.0)	71.2, CH
5′	4.23, overlap; 3.32, overlap	64.7, CH_2_	4.04, dd (15.0, 8.5); 3.27, overlap	62.8, CH_2_	3.46, m	75.7, CH	3.24, m	77.8, CH
6′					4.32, dd (11.5, 1.5); 4.14, dd (11.5, 6.0)	68.4, CH_2_	3.85, overlap; 3.67, dd (12.0, 5.5)	62.7, CH_2_
OMe	3.62, s	60.8, CH_3_	3.47, s	58.7, CH_3_	3.63, s	61.1, CH_3_	3.63, s	61.1, CH_3_

Measured at 500/125 MHz.

**Table 4:** The ^1^H NMR and ^13^C NMR data of compounds **8**–**10** in CD_3_OD (*δ* in ppm, *J* in Hz).

No.	8		9		10	
	*δ*_H_	*δ*_C_, Type	*δ*_H_	*δ*_C_, Type	*δ*_H_	*δ*_C_, Type
1	1.69, m; 1.00, m	39.3, CH_2_	1.69, m; 1.00, m	39.2, CH_2_	1.74, m; 0.99, m	39.5, CH_2_
2	1.72, m; 1.47, m	31.4, CH_2_	1.72, m; 1.47, m	31.4, CH_2_	1.73, m; 1.48, m	31.4, CH_2_
3	3.50, m	72.3, CH	3.51, m	72.3, CH	3.49, m	72.2, CH
4	2.10, m; 1.21, m	32.1, CH_2_	2.09, m; 1.22, m	32.1, CH	2.19, m; 1.20, m	32.3, CH_2_
5	1.57, m	44.3, CH	1.58, td (11.5, 2.5)	44.3, CH	1.04, m	53.6, CH
6	3.83, dd (11.5, 3.0)	69.5, CH	3.82, overlap	69.5, CH	3.62, td (11.0, 4.0)	67.7, CH
7	3.89, d (3.0)	76.5, CH	3.89, d (2.5)	76.5, CH	2.39, dd (13.5, 4.0); 1.38, dd (13.5, 11.0)	50.2, CH_2_
8		79.3, C		79.3, C		76.0, C
9	1.14, m	50.2, CH	1.14, m	50.1, CH	0.88, m	57.3, CH
10		37.6, C		37.6, C		37.8, C
11	1.81, m; 1.49, m	19.1, CH_2_	1.80, m; 1.48, m	19.2, CH_2_	1.70, m; 1.49	19.6, CH_2_
12	1.93, m; 1.13, m	43.0, CH_2_	1.93, m; 1.14, m	43.1, CH_2_	1.96, m; 1.26, m	42.9, CH_2_
13		44.4, C		44.4, C		45.5, C
14	1.41, d (5.5)	55.3, CH	1.41, d (5.5)	55.2, CH	1.30, d (10.5)	66.9, CH
15	4.49, dd (7.0, 5.5)	71.1, CH	4.50, dd (7.0, 5.5)	71.0, CH	4.21, td (10.5, 3.0)	69.9, CH
16	4.16, t (7.0)	73.1, CH	4.24, overlap	72.7, CH	1.92, m; 1.79, m	41.8, CH_2_
17	0.98, m	63.5, CH	1.02, m	62.3, CH	1.36, m	56.0, CH
18	1.28, s	17.8, CH_3_	1.25, s	17.8, CH_3_	0.97, s	15.5, CH_3_
19	0.99, s	13.9, CH_3_	0.99, s	13.9, CH_3_	1.02, s	14.2, CH_3_
20	2.61, m	34.5, CH	2.01, m	30.7, CH	1.34, m	36.4, CH
21	1.04, d (7.0)	20.3, CH_3_	0.98, d (6.5)	18.6, CH_3_	0.91, d (6.0)	19.0, CH_3_
22	5.51, dd (15.5, 7.5)	137.0, CH	1.78, m; 1.41, m	35.1, CH_2_	1.65, m; 0.97, m	33.1, CH_2_
23	5.31, dd (15.5, 8.5)	134.7, CH	2.01, m; 2.15, m	31.8, CH_2_	1.58, m; 1.27, m	29.0, CH_2_
24	2.26, m	35.3, CH		154.0, C	3.29, m	85.1, CH
25	1.63, m; 1.51, m	38.0, CH_2_	2.52, m	41.1, CH	1.83, m	32.2, CH
26	3.83, m; 3.52, m	69.0, CH_2_	3.83, dd (9.0, 6.0); 3.33, m	75.2, CH_2_	0.91, d (6.0)	18.4, CH_3_
27			1.09, d (6.5)	17.6, CH_3_	0.91, d (6.0)	18.3, CH_3_
28	0.99, d (6.5)	21.8, CH_3_	4.81, s; 4.76, s	109.3, CH2		
1′	4.20, d (7.5)	104.9, CH	4.22, overlap	105.1, CH	4.91, overlap	109.3, CH
2′	3.27, dd (9.0, 7.5)	74.1, CH	3.26, dd (9.0, 7.5)	74.2, CH	3.96, dd (4.5, 2.0)	83.9, CH
3′	3.17, t (9.0)	85.2, CH	3.19, t (9.0)	85.1, CH	3.85, dd (6.5, 4.5)	79.1, CH
4′	4.24, m	77.2, CH	4.25, m	77.2, CH	4.12, overlap	82.4, CH
5′	4.23, overlap; 3.32, overlap	64.8, CH_2_	4.20, overlap; 3.34, overlap	64.8, CH_2_	4.17, dd (9.5, 3.0); 4.10, overlap	68.7, CH_2_
OMe	3.62, s	60.8, CH_3_	3.62, s	60.8, CH_3_		

Measured at 500/125 MHz.

## Data Availability

Data are contained within the article or Supplementary Materials.

## Supplementary Materials

The following supporting information can be downloaded at: https://www.mdpi.com/article/10.3390/md22120545/s1. Figures S1–S8: NMR and MS spectra of compound **1**. Figures S9–S10: optical rotation data of **1** and its monosaccharide. Figures S11–S17: NMR and MS spectra of compound **2**. Figures S18–S19: optical rotation data of **2** and its monosaccharide. Figures S20–S26: NMR and MS spectra of compound **3**. Figures S27–S28: optical rotation data of **3** and GC-MS spectra of monosaccharide. Figures S29–S54: NMR, MS spectra, and optical rotation of compounds **4**–**6**. Figure S55: GC-MS spectra of monosaccharide of **6**. Figures S56–S88: NMR, MS spectra, and optical rotation of compounds **7**–**10**. Table S1: The anti-inflammatory activities of compounds **1**–**6** and **10.**
