# Supplementary material for: Pectiniferosides A–J: Diversified Glycosides of Polyhydroxy Steroids Isolated from the Sea Star Patiria (=Asterina) pectinifera
Source: Mar Drugs. 2024 Dec 3;22(12):545. doi: 10.3390/md22120545 (PMC11678026; doi:10.3390/md22120545)
Supplement: Supplementary file 1 [file marinedrugs-22-00545-s001.zip › marinedrugs-3323380-supplementary.pdf]

Pectiniferosides A–J: Diversified Glycosides of Polyhydroxy Steroids Isolated from the Sea Star *Patiria (=Asterina) pectinifera*

Ranran Zhang [1,2] Zhen Lu [1], Derui Wang [3], Zhi Yan [1], Xueting Sun [1], Xiaodong Li [1], Xiuli Yin [1], Song Wang [4], Ke Li [1,5,]*

[1] Yantai Institute of Coastal Zone Research, Chinese Academy of Sciences, Yantai 264003, China; rrzhang@yic.ac.cn (R.Z.); zlu@yic.ac.cn (Z.L.); zyan@yic.ac.cn (Z.Y.); sunxueting233@163.com (X.S.); xiaodongli@yic.ac.cn (X.L.); xlyin@yic.ac.cn (X.Y.)

[2] College of Resources and Environment, University of Chinese Academy of Sciences, Beijing 100049, China

[3] College of Marine Science, Beibu Gulf University, Qinzhou 535011, China; wdrui@bbgu.edu.cn

[4] Co-Innovation Center of Jiangsu Marine Bio-industry Technology, Jiangsu Ocean University, Lianyungang 222005, China; wangsong75@163.com

[5] Center for Ocean Mega-Science, Chinese Academy of Sciences, Qingdao 266071, China

* Correspondence: kli@yic.ac.cn; Tel.: +86-535-210-9286

Table of Contents

Figure S1 HR-ESI-MS and MS/MS spectra of **1** 5
Figure S2 ^{1}H NMR spectrum of **1** in CD_3OD (500 MHz)..7
Figure S3 ^{13}C NMR spectra of **1** in CD_3OD (125 MHz)..7
Figure S4 HSQC spectrum of **1** in CD_3OD (500 MHz)..8
Figure S5 ^{1}H-^{1}H COSY spectrum of **1** in CD_3OD (500 MHz) 8
Figure S6 HMBC spectrum of **1** in CD_3OD (500 MHz)...9
Figure S7 NOESY spectrum of **1** in CD_3OD (500 MHz) 9
Figure S8 NOESY spectrum of **1** in $(CD_3)_2SO$ (500 MHz) 10
Figure S9 $[\alpha]_D$ data of **1** 11
Figure S10 $[\alpha]_D$ data of monosaccharide of **1** 11
Figure S11 HR-ESI-MS and MS/MS spectra of **2** 12
Figure S12 ^{1}H NMR spectrum of **2** in CD_3OD (500 MHz) 14
Figure S13 ^{13}C NMR spectra of **2** in CD_3OD (125 MHz) 14
Figure S14 HSQC spectrum of **2** in CD_3OD (500 MHz)..15
Figure S15 ^{1}H-^{1}H COSY spectrum of **2** in CD_3OD (500 MHz) 15
Figure S16 HMBC spectrum of **2** in CD_3OD (500 MHz)...16
Figure S17 NOESY spectrum of **2** in CD_3OD (500 MHz) 16
Figure S18 $[\alpha]_D$ data of **2**...17
Figure S19 $[\alpha]_D$ data of monosaccharide of **2** 17
Figure S20 HR-ESI-MS and MS/MS spectra of **3** 18
Figure S21 ^{1}H NMR spectrum of **3** in CD_3OD (500 MHz) 20
Figure S22 ^{13}C NMR spectra of **3** in CD_3OD (125 MHz) 20
Figure S23 HSQC spectrum of **3** in CD_3OD (500 MHz)..21
Figure S24 ^{1}H-^{1}H COSY spectrum of **3** in CD_3OD (500 MHz) 21
Figure S25 HMBC spectrum of **3** in CD_3OD (500 MHz)...22
Figure S26 NOESY spectrum of **3** in CD_3OD (500 MHz) 22
Figure S27 $[\alpha]_D$ data of **3**...23
Figure S28 GC-MS analysis of L-arabinose and monosaccharide of **3** 23
Figure S29 HR-ESI-MS and MS/MS spectra of **4** 24
Figure S30 ^{1}H NMR spectrum of **4** in CD_3OD (500 MHz)..26
Figure S31 ^{13}C NMR spectra of **4** in CD_3OD (125 MHz) 26
Figure S32 HSQC spectrum of **4** in CD_3OD (500 MHz)..27
Figure S33 ^{1}H-^{1}H COSY spectrum of **4** in CD_3OD (500 MHz) 27
Figure S34 HMBC spectrum of **4** in CD_3OD (500 MHz)...28
Figure S35 NOESY spectrum of **4** in CD_3OD (500 MHz) 28
Figure S36 NOESY spectrum of **4** in $(CD_3)_2SO$ (500 MHz) 29
Figure S37 $[\alpha]_D$ data of **4** 29

Figure S38 HR-ESI-MS and MS/MS spectra of **5** 30
Figure S39 ^{1}H NMR spectrum of **5** in CD_3OD (500 MHz) 32
Figure S40 ^{13}C NMR spectra of **5** in CD_3OD (125 MHz) 32
Figure S41 HSQC spectrum of **5** in CD_3OD (500 MHz) 33
Figure S42 ^{1}H-^{1}H COSY spectrum of **5** in CD_3OD (500 MHz) 33
Figure S43 HMBC spectrum of **5** in CD_3OD (500 MHz) 34
Figure S44 NOESY spectrum of **5** in CD_3OD (500 MHz) 34
Figure S45 [α] D data of **5** 35
Figure S46 HR-ESI-MS and MS/MS spectra of **6** 36
Figure S47 ^{1}H NMR spectrum of **6** in CD_3OD (500 MHz) 38
Figure S48 ^{13}C NMR spectra of **6** in CD_3OD (125 MHz) 38
Figure S49 HSQC spectrum of **6** in CD_3OD (500 MHz) 39
Figure S50 ^{1}H-^{1}H COSY spectrum of **6** in CD_3OD (500 MHz) 39
Figure S51 HMBC spectrum of **6** in CD_3OD (500 MHz) 40
Figure S52 NOESY spectrum of **6** in CD_3OD (500 MHz) 40
Figure S53 NOESY spectrum of **6** in $(CD_3)_2SO$ (500 MHz) 41
Figure S54 [α] D data of **6** 41
Figure S55 GC-MS analysis of D-3-*O*-methyl-glucose and monosaccharide of **6** 42
Figure S56 HR-ESI-MS and MS/MS spectra of **7** 43
Figure S57 ^{1}H NMR spectrum of **7** in CD_3OD (500 MHz) 45
Figure S58 ^{13}C NMR spectra of **7** in CD_3OD (125 MHz) 45
Figure S59 HSQC spectrum of **7** in CD_3OD (500 MHz) 46
Figure S60 ^{1}H-^{1}H COSY spectrum of **7** in CD_3OD (500 MHz) 46
Figure S61 HMBC spectrum of **7** in CD_3OD (500 MHz) 47
Figure S62 NOESY spectrum of **7** in CD_3OD (500 MHz) 47
Figure S63 [α] D data of **7** 48
Figure S64 HR-ESI-MS and MS/MS spectra of **8** 49
Figure S65 ^{1}H NMR spectrum of **8** in CD_3OD (500 MHz) 51
Figure S66 ^{13}C NMR spectra of **8** in CD_3OD (125 MHz) 51
Figure S67 HSQC spectrum of **8** in CD_3OD (500 MHz) 52
Figure S68 ^{1}H-^{1}H COSY spectrum of **8** in CD_3OD (500 MHz) 52
Figure S69 HMBC spectrum of **8** in CD_3OD (500 MHz) 53
Figure S70 NOESY spectrum of **8** in CD_3OD (500 MHz) 53
Figure S71 [α] D data of **8** 54
Figure S72 HR-ESI-MS and MS/MS spectra of **9** 55
Figure S73 ^{1}H NMR spectrum of **9** in CD_3OD (500 MHz) 57
Figure S74 ^{13}C NMR spectra of **9** in CD_3OD (125 MHz) 57
Figure S75 HSQC spectrum of **9** in CD_3OD (500 MHz) 58
Figure S76 ^{1}H-^{1}H COSY spectrum of **9** in CD_3OD (500 MHz) 58

Figure S77 HMBC spectrum of **9** in CD_3OD (500 MHz)..59
Figure S78 NOESY spectrum of **9** in CD_3OD (500 MHz) 59
Figure S79 [α] D data of **9**..60
Figure S80 HR-ESI-MS and MS/MS spectra of **10** 61
Figure S81 ^1H NMR spectrum of **10** in CD_3OD (500 MHz) 63
Figure S82 ^{13}C NMR spectra of **10** in CD_3OD (125 MHz) 63
Figure S83 HSQC spectrum of **10** in CD_3OD (500 MHz)...64
Figure S84 ^1H-^1H COSY spectrum of **10** in CD_3OD (500 MHz) 64
Figure S85 HMBC spectrum of **10** in CD_3OD (500 MHz)...65
Figure S86 NOESY spectrum of **10** in CD_3OD (500 MHz) 65
Figure S87 NOESY spectrum of **10** in $(CD_3)_2SO$ (500 MHz) 66
Figure S88 [α] D data of **10** 66
Table S1 The inhibitory effects of compounds **1-6** and **10** on nitric oxide (NO) production in RAW264.7 cell activated by LPS..67

Figure S1 HR-ESI-MS and MS/MS spectra of **1**

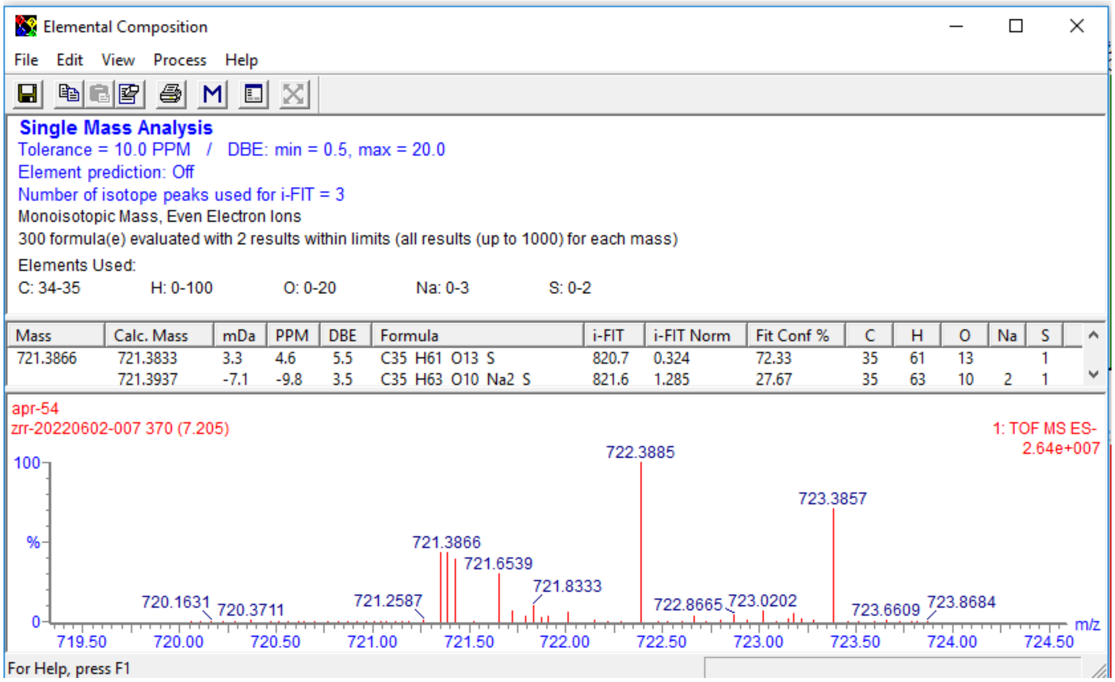

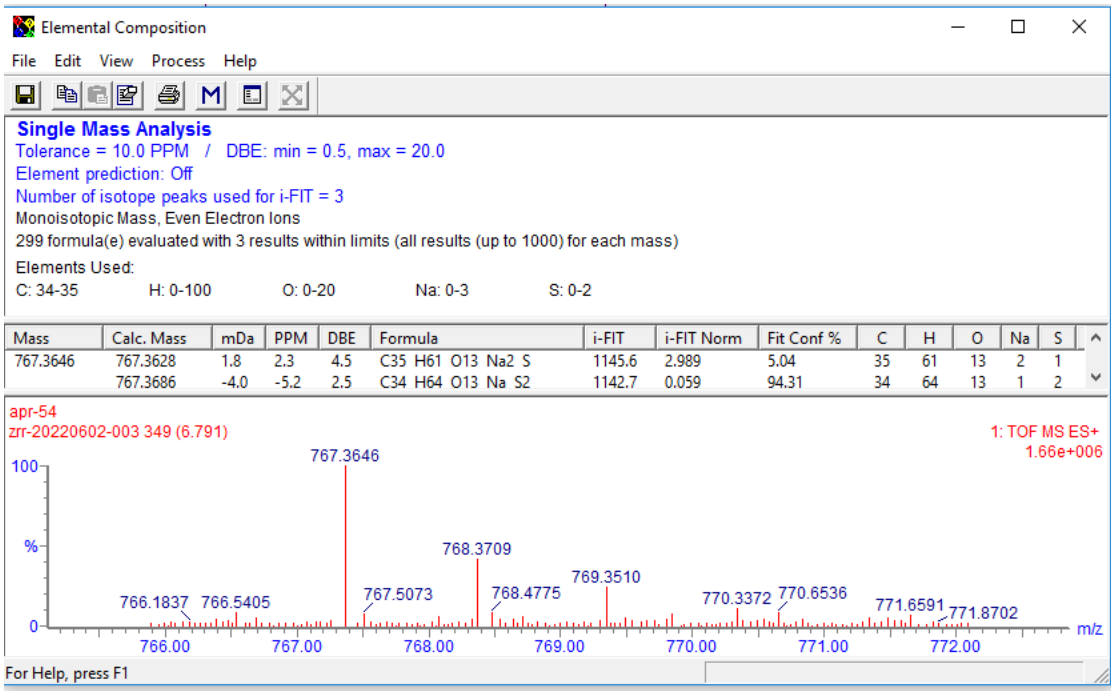

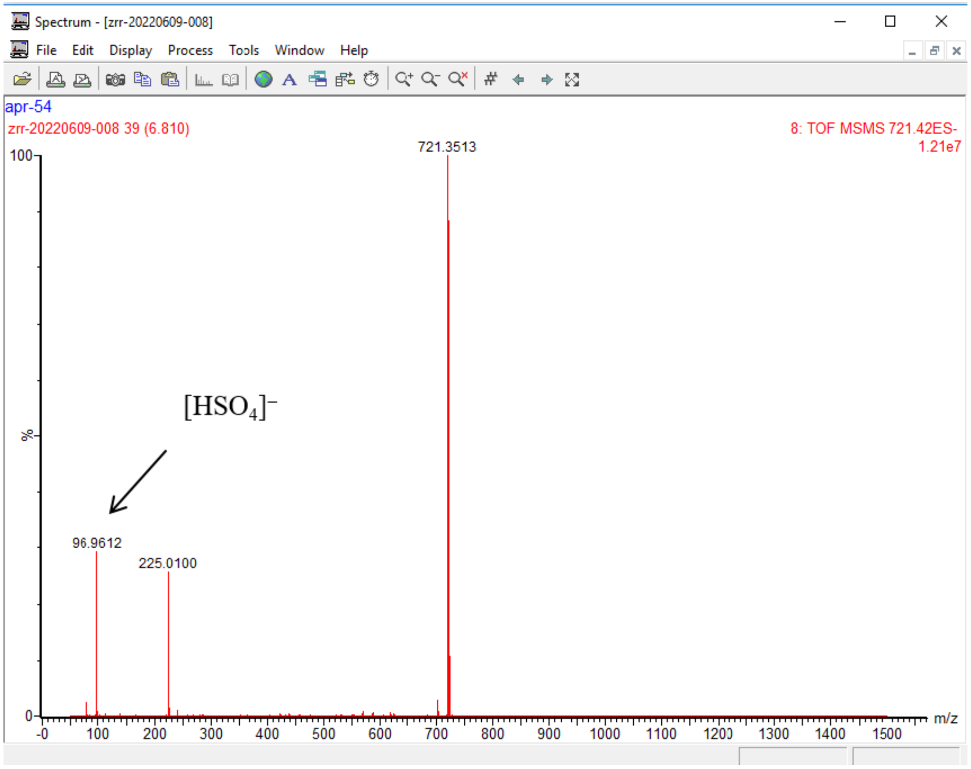
Spectrum - [zrr-20220609-008]
apr-54
zrr-20220609-008 39 (6.810)
8: TOF MSMS 721.42ES-
1.21e7
721.3513
[HSO4]⁻
96.9612
225.0100
m/z

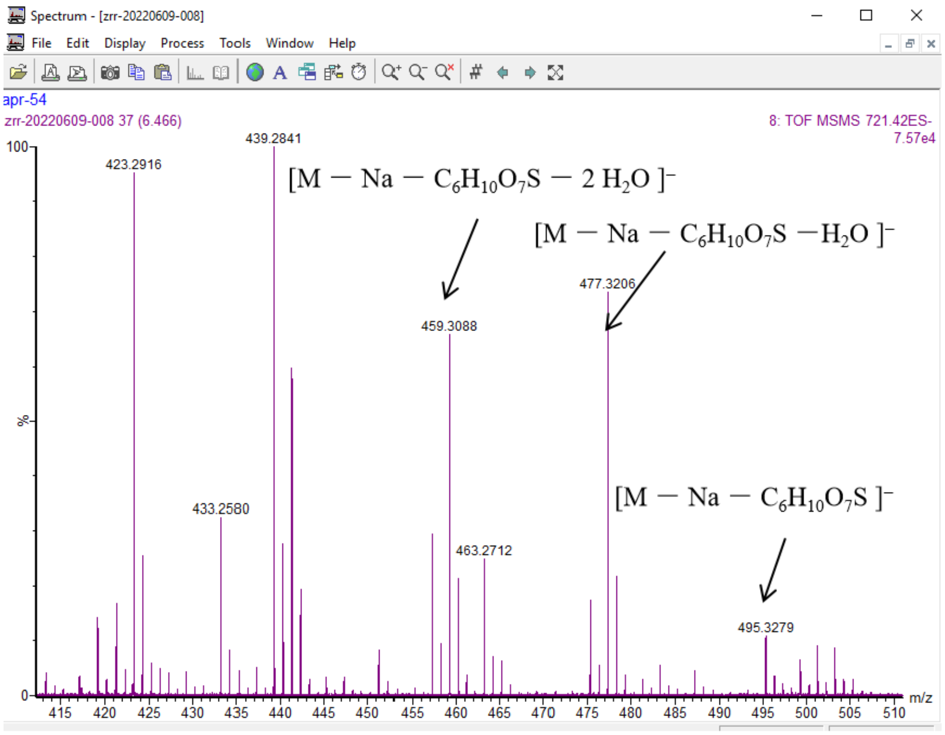
Spectrum - [zrr-20220609-008]
apr-54
zrr-20220609-008 37 (6.466)
8: TOF MSMS 721.42ES-
7.57e4
439.2841
423.2916
[M − Na − C6H10O7S − 2 H2O]⁻
[M − Na − C6H10O7S −H2O]⁻
477.3206
459.3088
[M − Na − C6H10O7S]⁻
433.2580
463.2712
495.3279
m/z

Figure S2 ^{1}H NMR spectrum of **1** in CD_3OD (500 MHz)

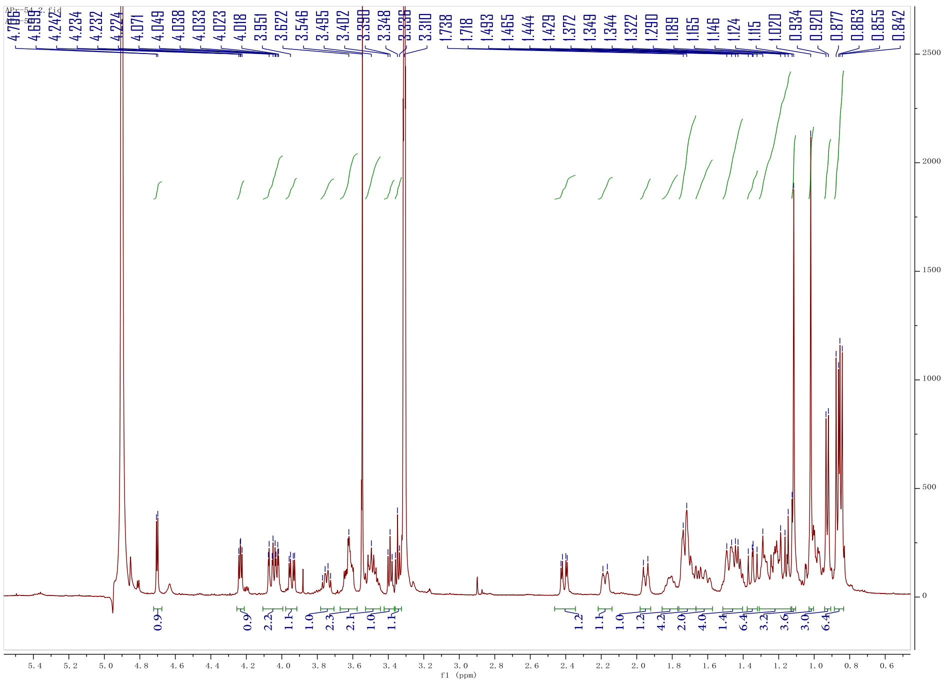

Figure S3 ^{13}C NMR spectra of **1** in CD_3OD (125 MHz)

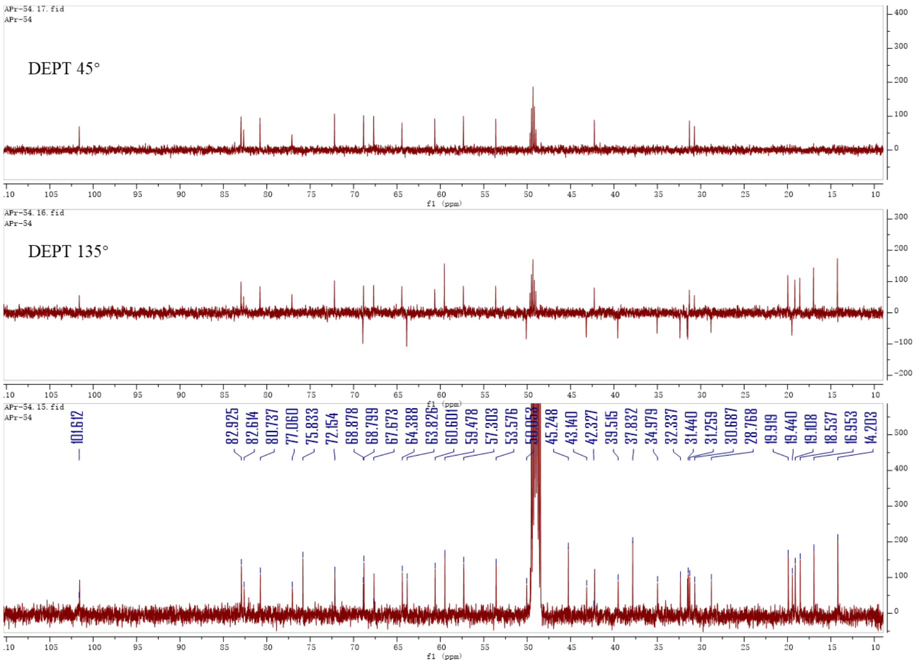

Figure S4 HSQC spectrum of **1** in CD_3OD (500 MHz)

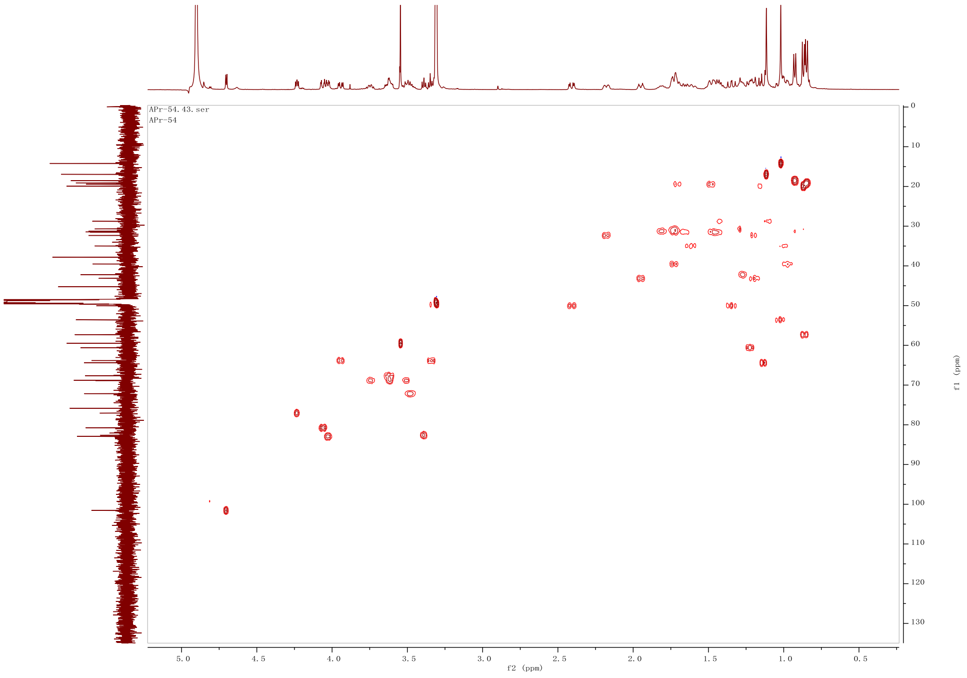

Figure S5 ^1H-^1H COSY spectrum of **1** in CD_3OD (500 MHz)

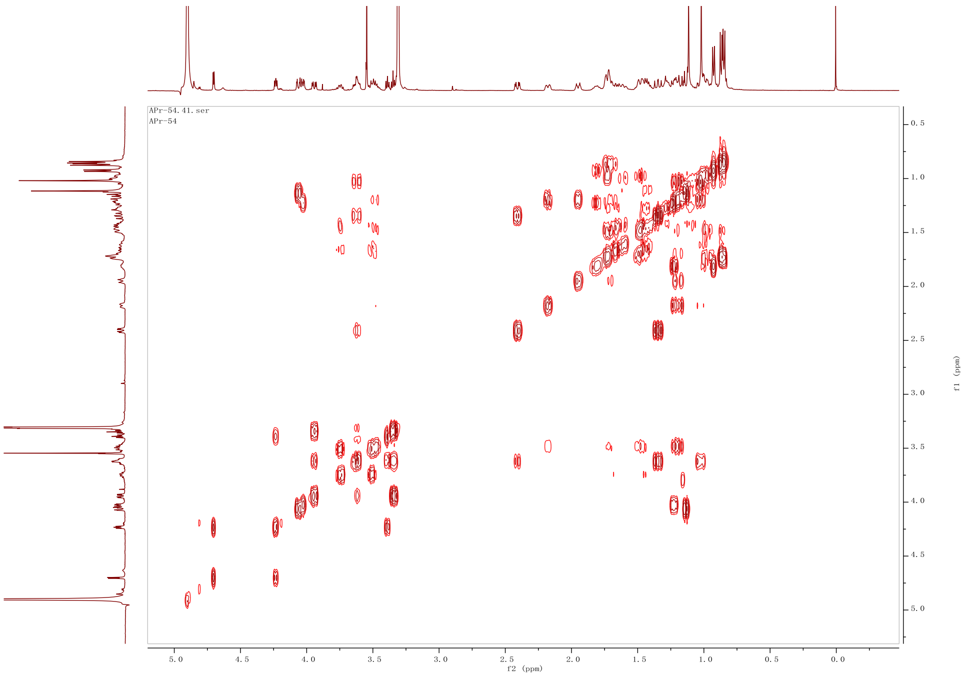

Figure S6 HMBC spectrum of **1** in CD_3OD (500 MHz)

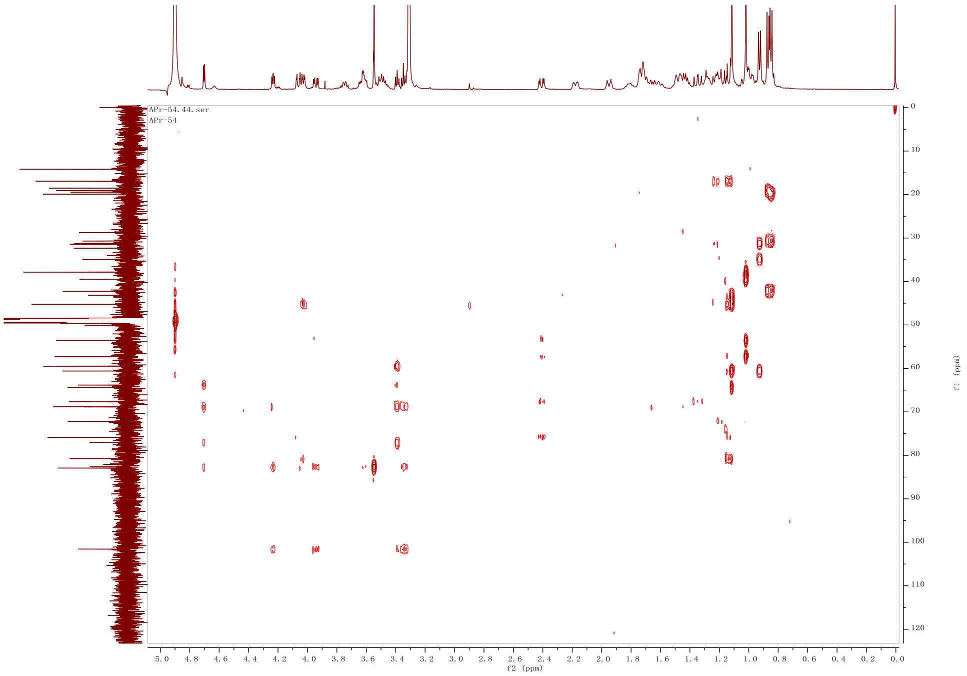

Figure S7 NOESY spectrum of **1** in CD_3OD (500 MHz)

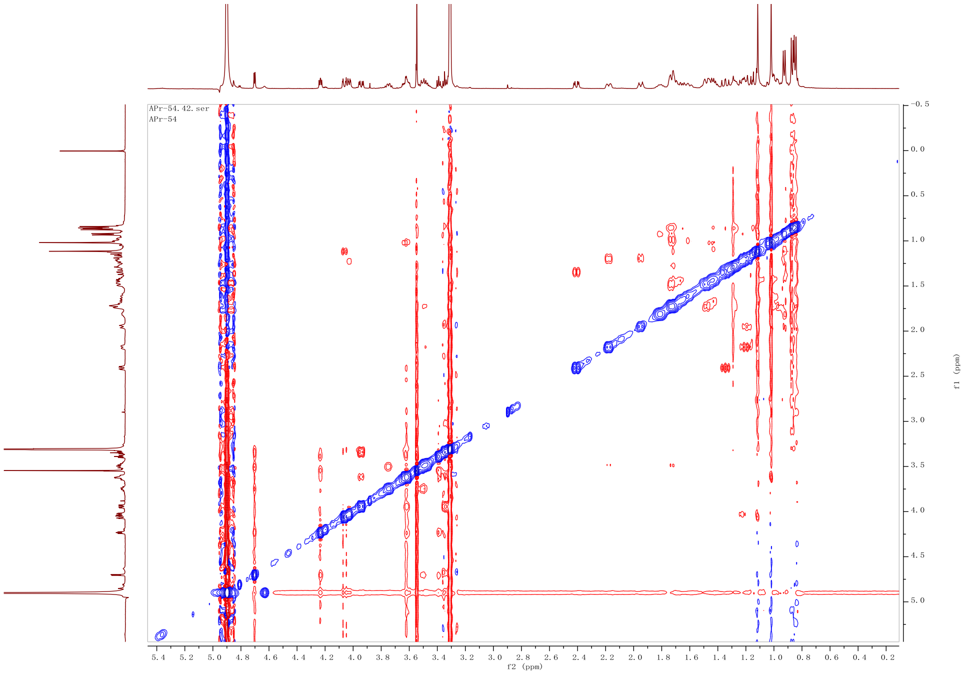

Figure S8 NOESY spectrum of **1** in $(CD_3)_2SO$ (500 MHz)

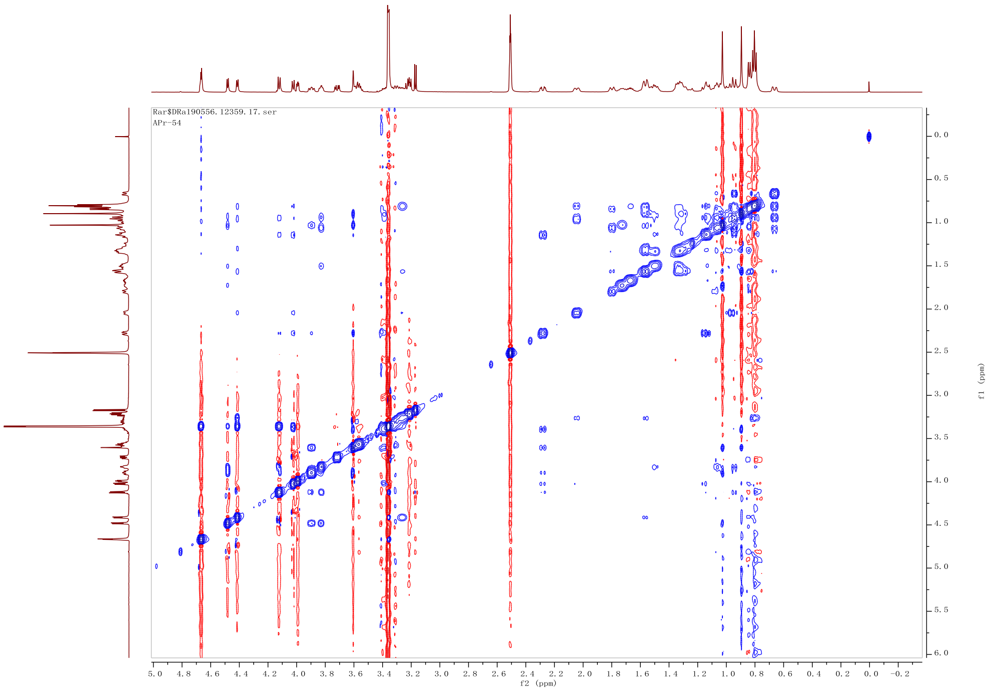

Figure S9 [α] $_D$ data of **1**

Rudolph Research Analytical

This sample was measured on an Autopol VI, Serial #91058
Manufactured by Rudolph Research Analytical, Hackettstown, NJ, USA.

Measurement Date : Tuesday, 18-JUL-2023

Set Temperature : 25.0

Time Delay : Disabled

Delay between Measurement : Disabled

n	Average	Std.Dev.	% RSD	Maximum	Minimum
5	13.00	0.45	3.46	13.33	12.50

S.No	Sample ID	Time	Result	Scale	OR °Arc	WLG.nm	Lg.mm	Conc.g/100ml	Temp.
1	APR-54	09:09:08 PM	12.50	SR	0.015	589	100.00	0.120	25.0
2	APR-54	09:09:14 PM	13.33	SR	0.016	589	100.00	0.120	25.0
3	APR-54	09:09:20 PM	12.50	SR	0.015	589	100.00	0.120	25.0
4	APR-54	09:09:27 PM	13.33	SR	0.016	589	100.00	0.120	25.0
5	APR-54	09:09:33 PM	13.33	SR	0.016	589	100.00	0.120	25.0

Figure S10 [α] $_D$ data of monosaccharide of **1**

Rudolph Research Analytical

This sample was measured on an Autopol VI, Serial #91058
Manufactured by Rudolph Research Analytical, Hackettstown, NJ, USA.

Measurement Date : Saturday, 23-MAR-2024

Set Temperature : OFF

Time Delay : Disabled

Delay between Measurement : Disabled

n	Average	Std.Dev.	% RSD	Maximum	Minimum
5	44.25	0.68	1.53	45.00	43.75

S.No	Sample ID	Time	Result	Scale	OR °Arc	WLG.nm	Lg.mm	Conc.g/100ml	Temp.
1	APR-54G	12:27:21 PM	43.75	SR	0.035	589	100.00	0.080	23.2
2	APR-54G	12:27:28 PM	45.00	SR	0.036	589	100.00	0.080	23.2
3	APR-54G	12:27:34 PM	43.75	SR	0.035	589	100.00	0.080	23.3
4	APR-54G	12:27:40 PM	43.75	SR	0.035	589	100.00	0.080	23.3
5	APR-54G	12:27:47 PM	45.00	SR	0.036	589	100.00	0.080	23.2

Figure S11 HR-ESI-MS and MS/MS spectra of **2**

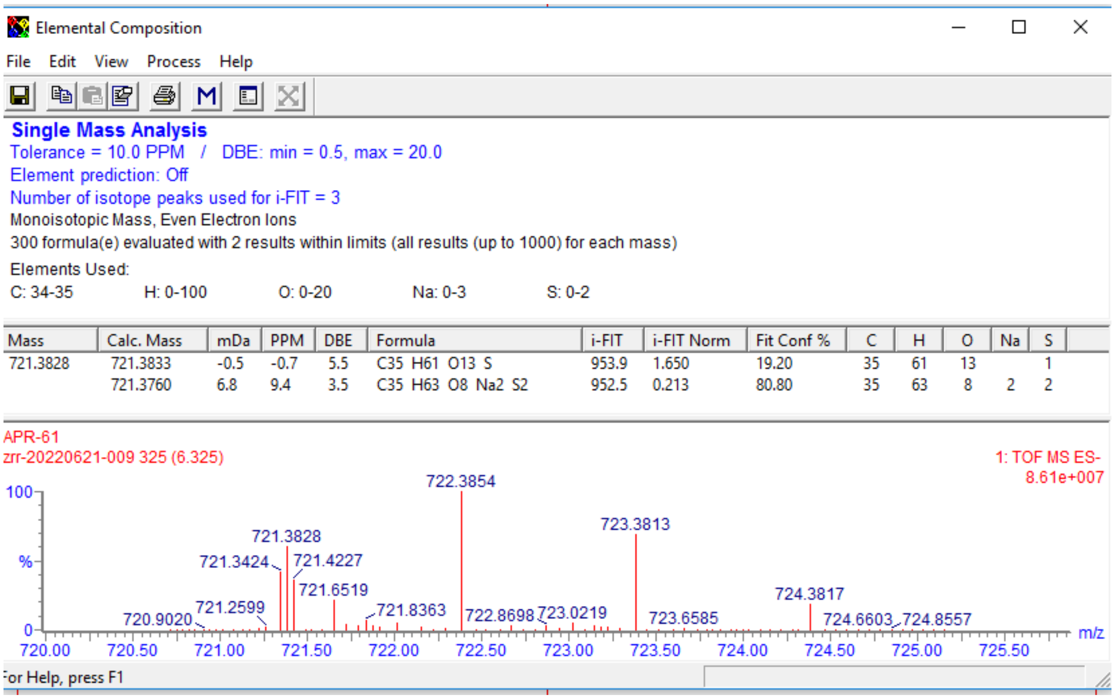

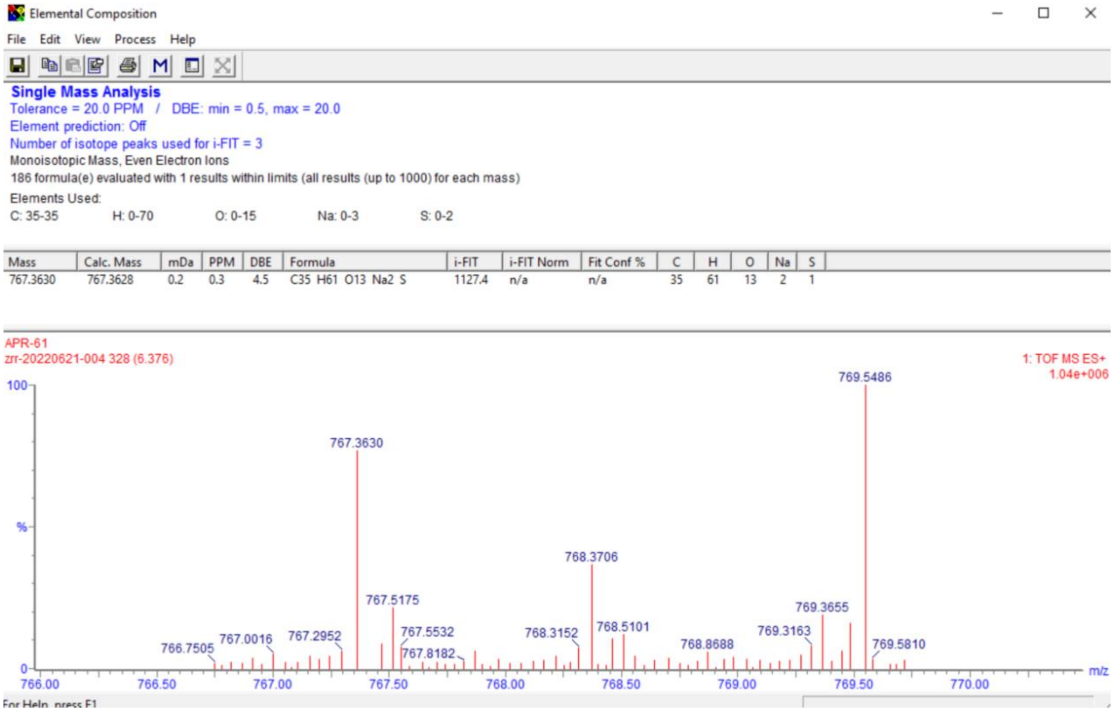

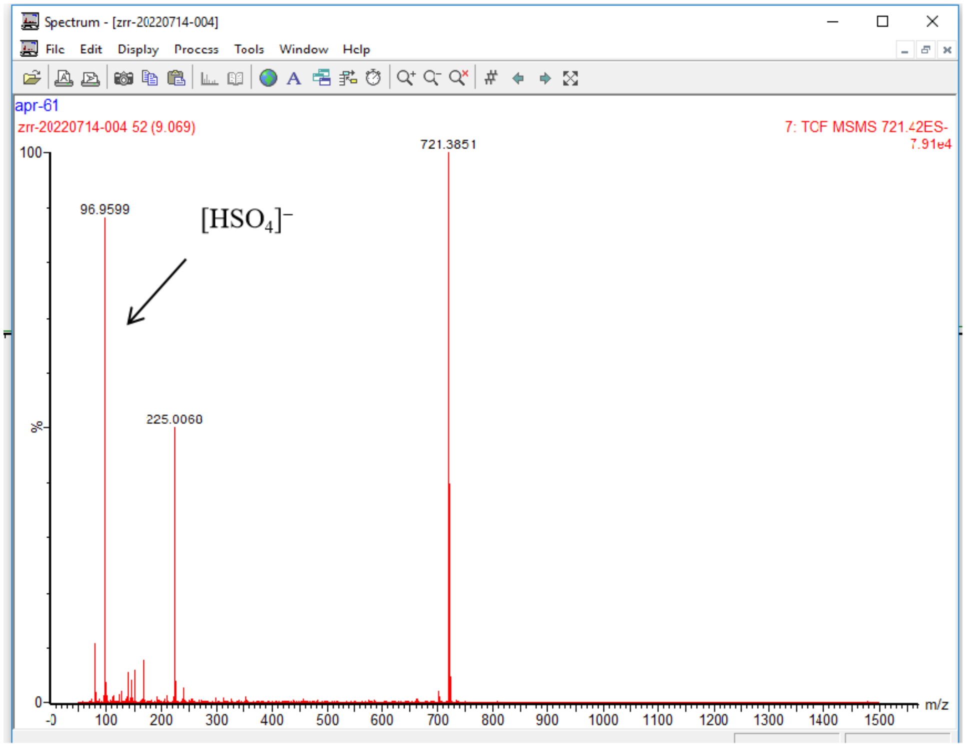
Spectrum - [zrr-20220714-004]
File Edit Display Process Tools Window Help
apr-61
zrr-20220714-004 52 (9.069)
7: TOF MSMS 721.42ES-
7.91e4
721.3851
96.9599
$[HSO_4]^-$
225.0060
m/z

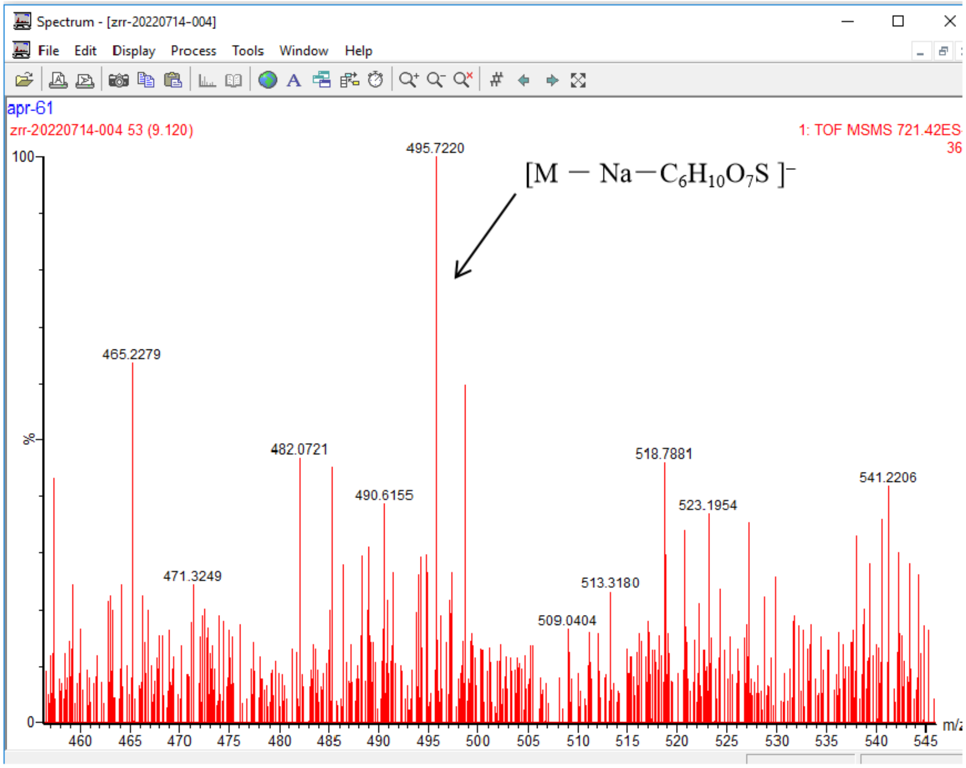
Spectrum - [zrr-20220714-004]
File Edit Display Process Tools Window Help
apr-61
zrr-20220714-004 53 (9.120)
1: TOF MSMS 721.42ES
36
495.7220
$[M - Na - C_6H_{10}O_7S]^-$
465.2279
482.0721
490.6155
471.3249
518.7881
523.1954
541.2206
513.3180
509.0404

Figure S12 ^{1}H NMR spectrum of **2** in CD_3OD (500 MHz)

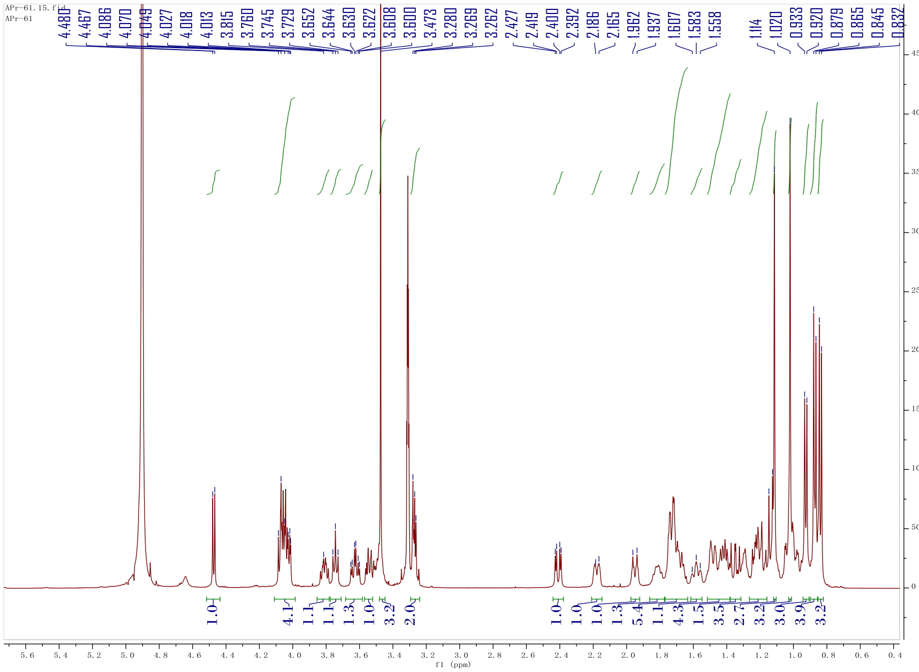

Figure S13 ^{13}C NMR spectra of **2** in CD_3OD (125 MHz)

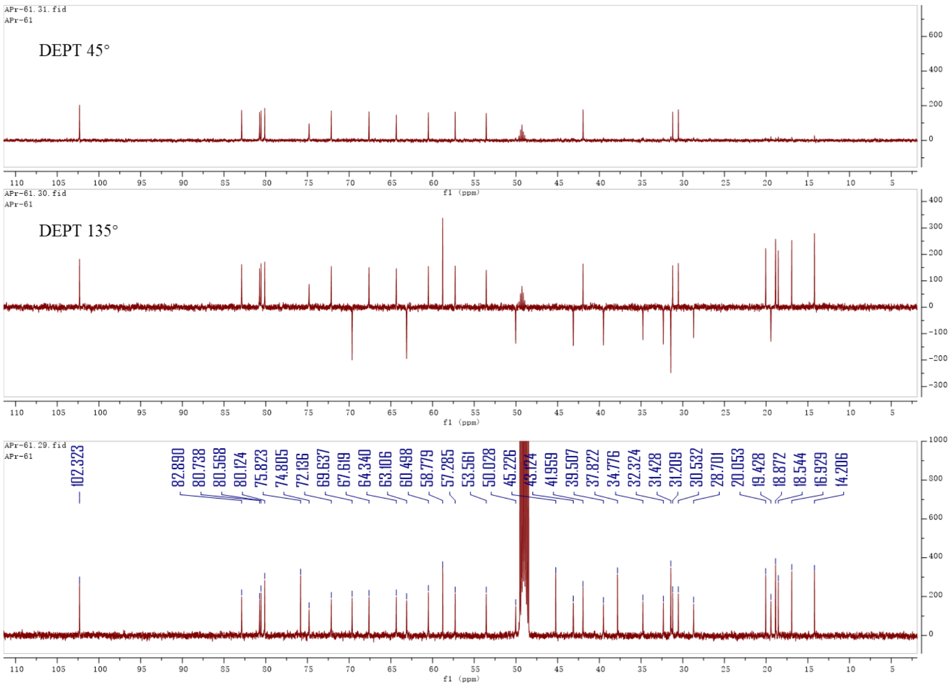

Figure S14 HSQC spectrum of **2** in CD_3OD (500 MHz)

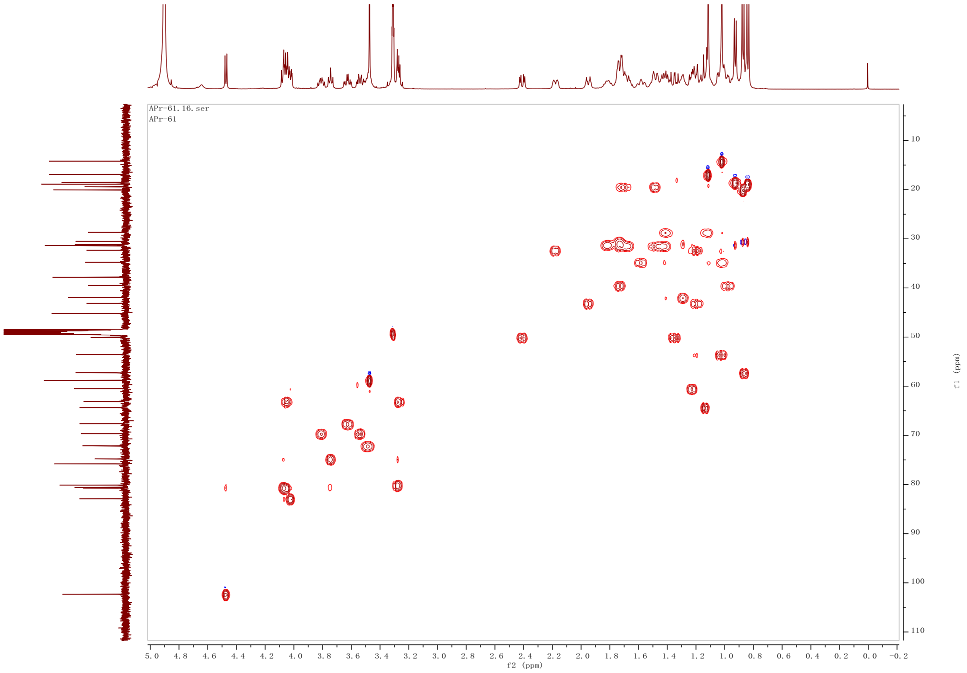

Figure S15 ^{1}H-^{1}H COSY spectrum of **2** in CD_3OD (500 MHz)

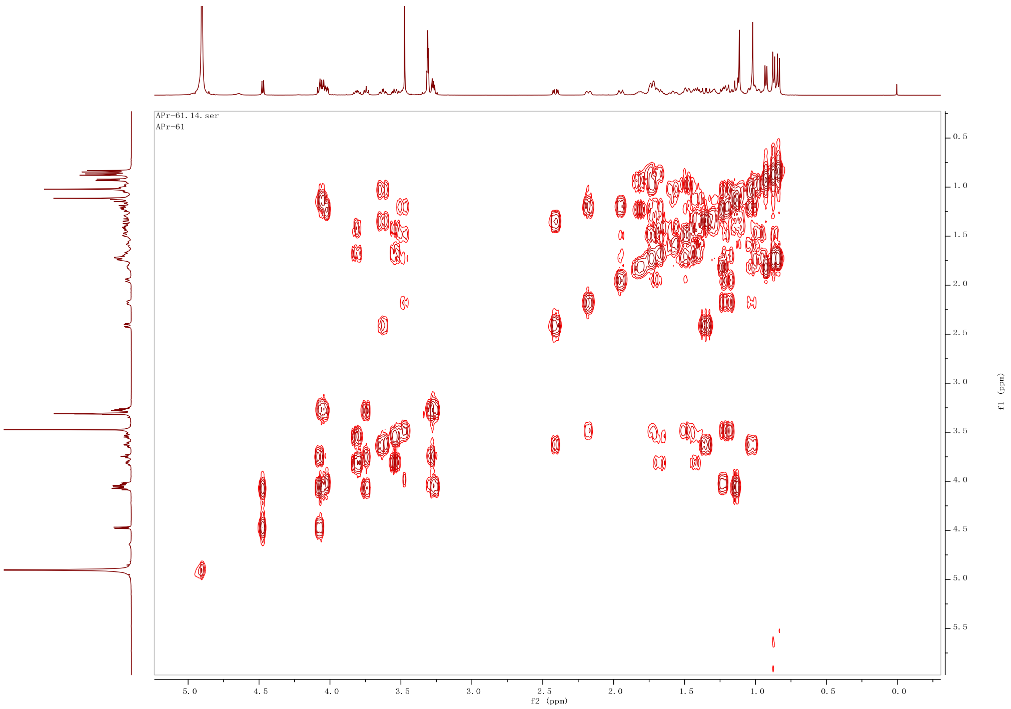

Figure S16 HMBC spectrum of **2** in CD_3OD (500 MHz)

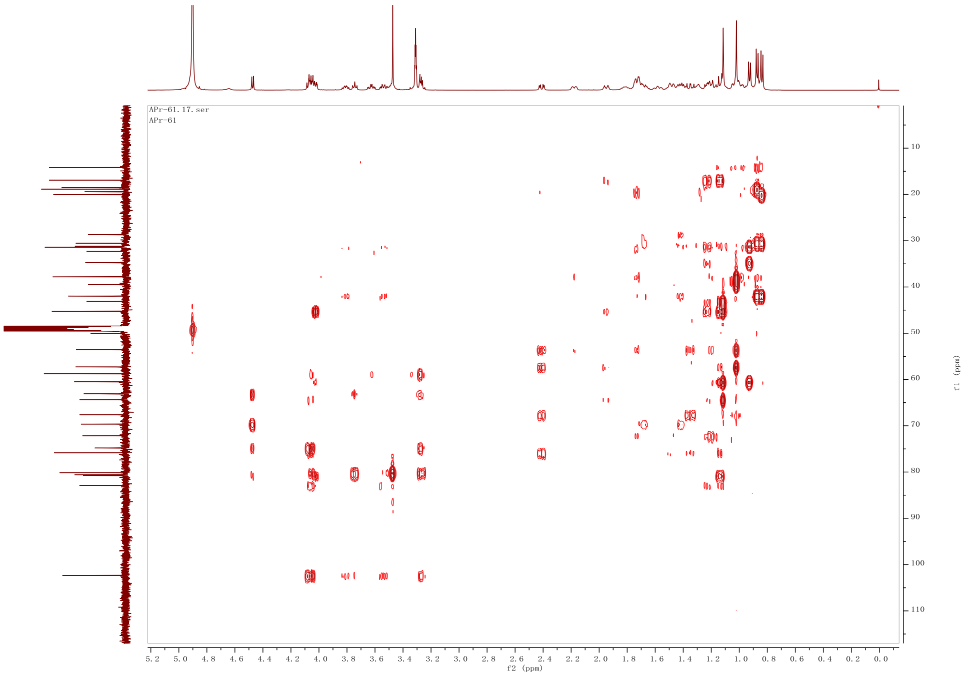

Figure S17 NOESY spectrum of **2** in CD_3OD (500 MHz)

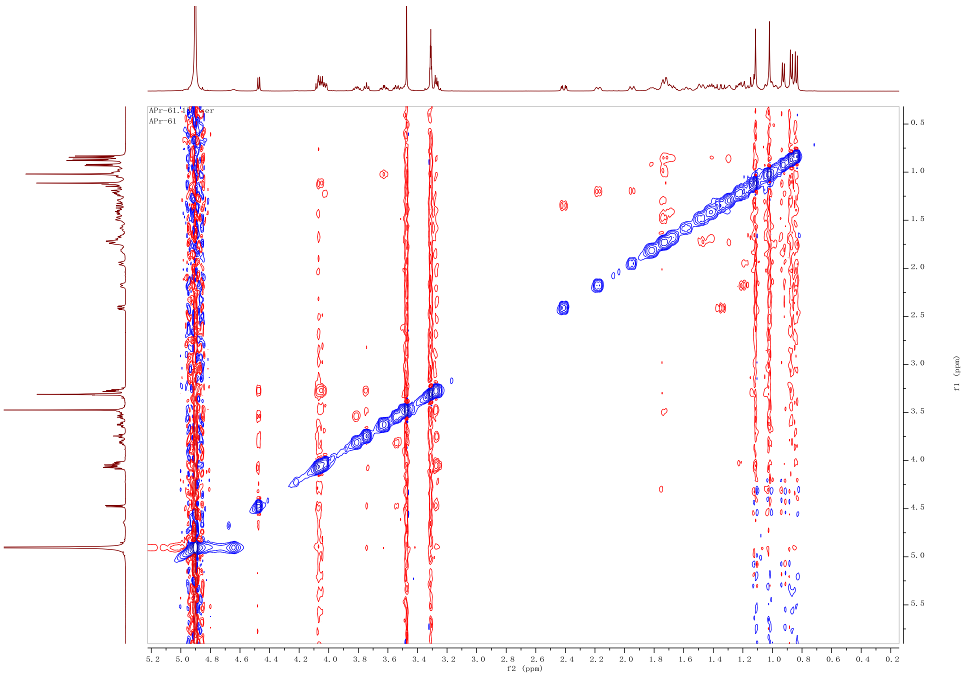

Figure S18 [α] $_D$ data of **2**

Rudolph Research Analytical

This sample was measured on an Autopol VI, Serial #91058
Manufactured by Rudolph Research Analytical, Hackettstown, NJ, USA.

Measurement Date : Wednesday, 19-JUL-2023

Set Temperature : OFF

Time Delay : Disabled

Delay between Measurement : Disabled

n	Average	Std.Dev.	% RSD	Maximum	Minimum
5	19.50	0.00	0.00	19.50	19.50

S.No	Sample ID	Time	Result	Scale	OR °Arc	WLG.nm	Lg.mm	Conc.g/100ml	Temp.
1	APR-61	12:16:45 PM	19.50	SR	0.039	589	100.00	0.200	25.0
2	APR-61	12:16:52 PM	19.50	SR	0.039	589	100.00	0.200	25.0
3	APR-61	12:16:58 PM	19.50	SR	0.039	589	100.00	0.200	25.0
4	APR-61	12:17:04 PM	19.50	SR	0.039	589	100.00	0.200	25.0
5	APR-61	12:17:11 PM	19.50	SR	0.039	589	100.00	0.200	25.0

Figure S19 [α] $_D$ data of monosaccharide of **2**

Rudolph Research Analytical

This sample was measured on an Autopol VI, Serial #91058
Manufactured by Rudolph Research Analytical, Hackettstown, NJ, USA.

Measurement Date : Saturday, 23-MAR-2024

Set Temperature : OFF

Time Delay : Disabled

Delay between Measurement : Disabled

n	Average	Std.Dev.	% RSD	Maximum	Minimum
5	73.20	13.01	17.77	88.00	54.00

S.No	Sample ID	Time	Result	Scale	OR °Arc	WLG.nm	Lg.mm	Conc.g/100ml	Temp.
1	APR-61G	12:35:56 PM	88.00	SR	0.044	589	100.00	0.050	23.3
2	APR-61G	12:36:02 PM	72.00	SR	0.036	589	100.00	0.050	23.3
3	APR-61G	12:36:08 PM	82.00	SR	0.041	589	100.00	0.050	23.3
4	APR-61G	12:36:15 PM	70.00	SR	0.035	589	100.00	0.050	23.3
5	APR-61G	12:36:21 PM	54.00	SR	0.027	589	100.00	0.050	23.3

Figure S20 HR-ESI-MS and MS/MS spectra of **3**

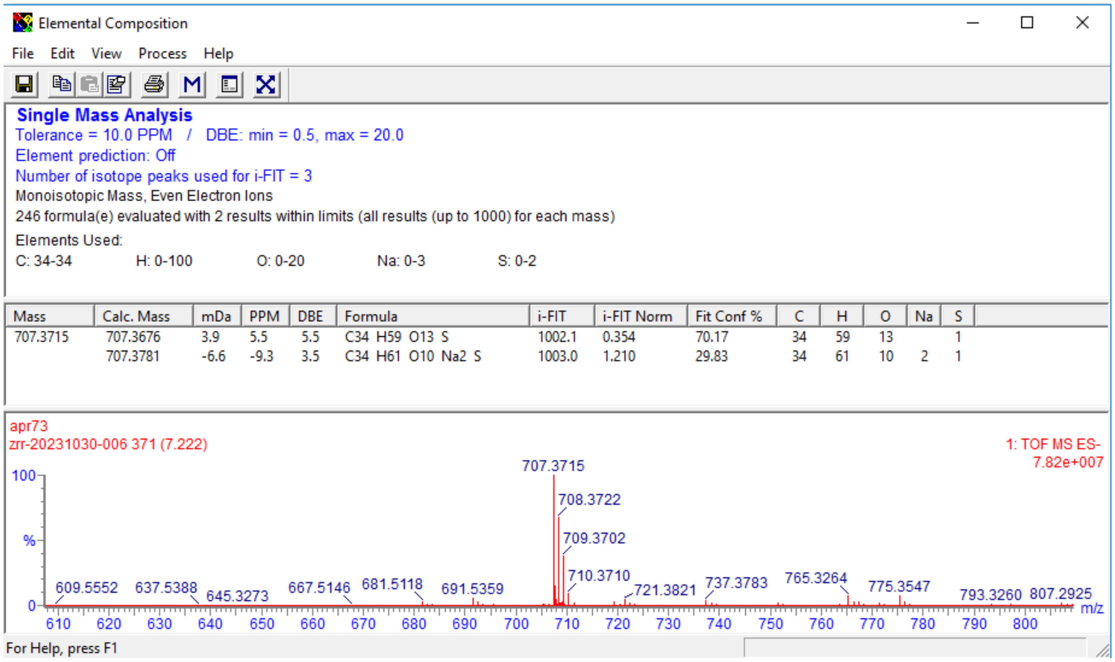

Mass	Calc. Mass	mDa	PPM	DBE	Formula	i-FIT	i-FIT Norm	Fit Conf %	C	H	O	Na	S
707.3715	707.3676	3.9	5.5	5.5	C34 H59 O13 S	1002.1	0.354	70.17	34	59	13		1
	707.3781	-6.6	-9.3	3.5	C34 H61 O10 Na2 S	1003.0	1.210	29.83	34	61	10	2	1

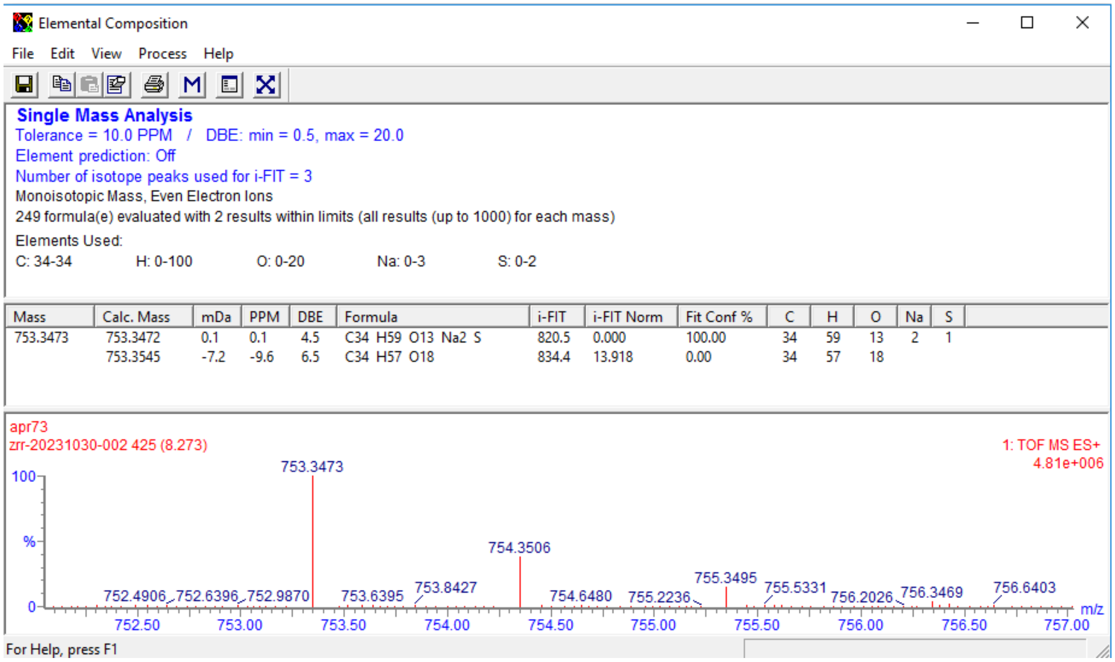

Mass	Calc. Mass	mDa	PPM	DBE	Formula	i-FIT	i-FIT Norm	Fit Conf %	C	H	O	Na	S
753.3473	753.3472	0.1	0.1	4.5	C34 H59 O13 Na2 S	820.5	0.000	100.00	34	59	13	2	1
	753.3545	-7.2	-9.6	6.5	C34 H57 O18	834.4	13.918	0.00	34	57	18		

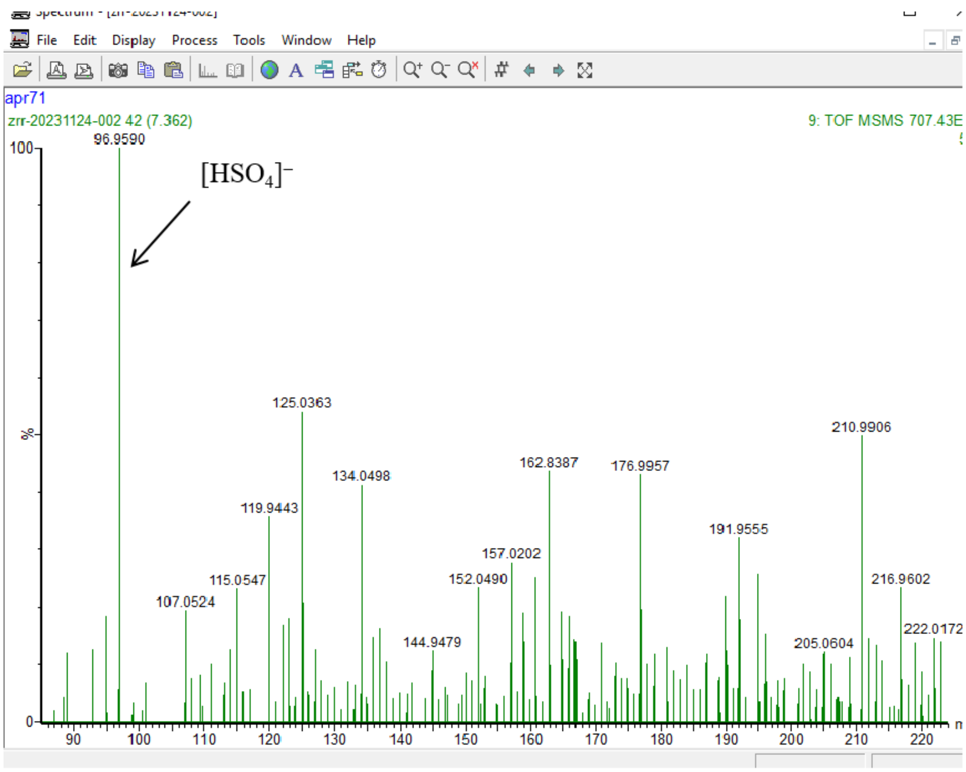

apr71
zrr-20231124-002 42 (7.362)
9: TOF MSMS 707.43E
96.9590
[HSO_4]⁻
125.0363
210.9906
134.0498
162.8387
176.9957
119.9443
191.9555
157.0202
115.0547
152.0490
216.9602
107.0524
222.0172
144.9479
205.0604

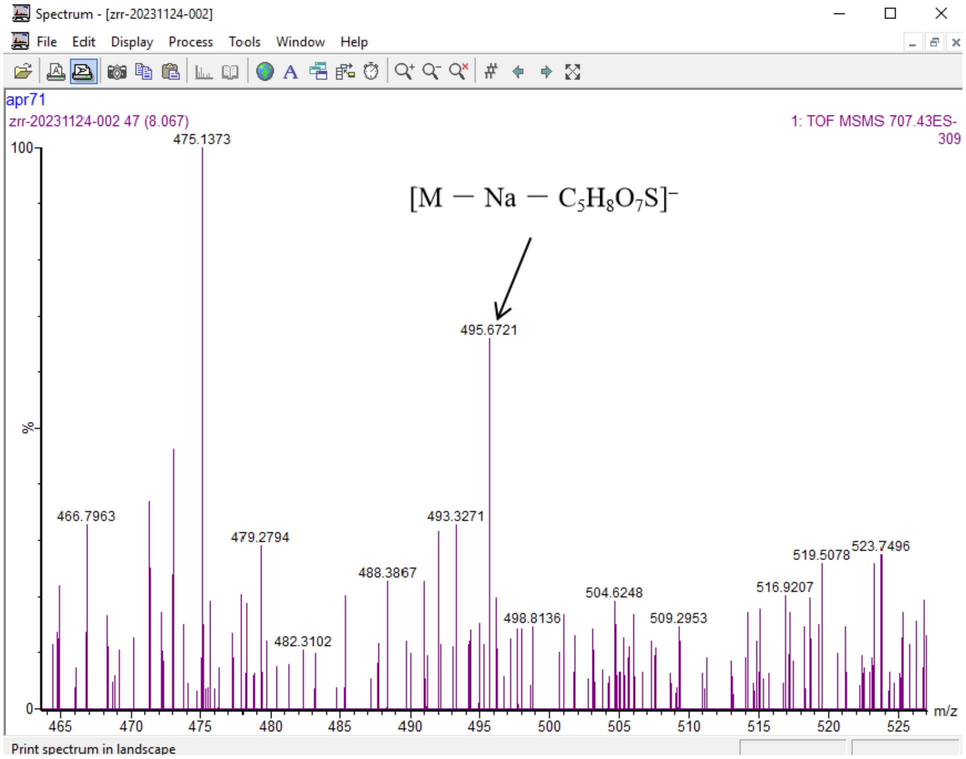

Spectrum - [zrr-20231124-002]
apr71
zrr-20231124-002 47 (8.067)
1: TOF MSMS 707.43ES-
309
475.1373
[M − Na − $C_5H_8O_7S$]⁻
495.6721
466.7963
493.3271
479.2794
519.5078
523.7496
488.3867
504.6248
516.9207
498.8136
509.2953
482.3102
m/z
Print spectrum in landscape

Figure S21 ^{1}H NMR spectrum of **3** in CD_3OD (500 MHz)

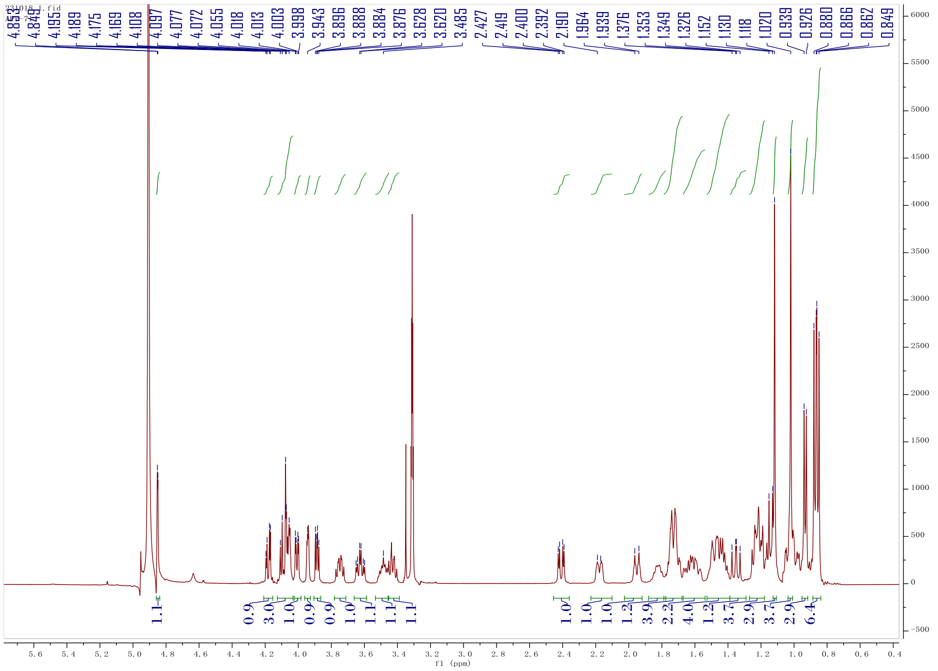

Figure S22 ^{13}C NMR spectra of **3** in CD_3OD (125 MHz)

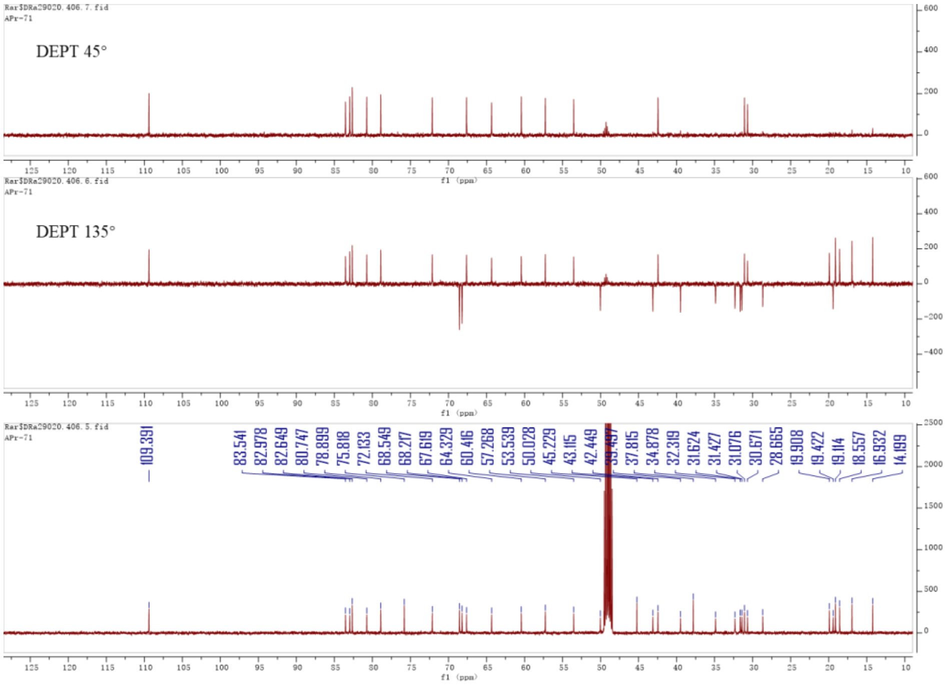

Figure S23 HSQC spectrum of **3** in CD_3OD (500 MHz)

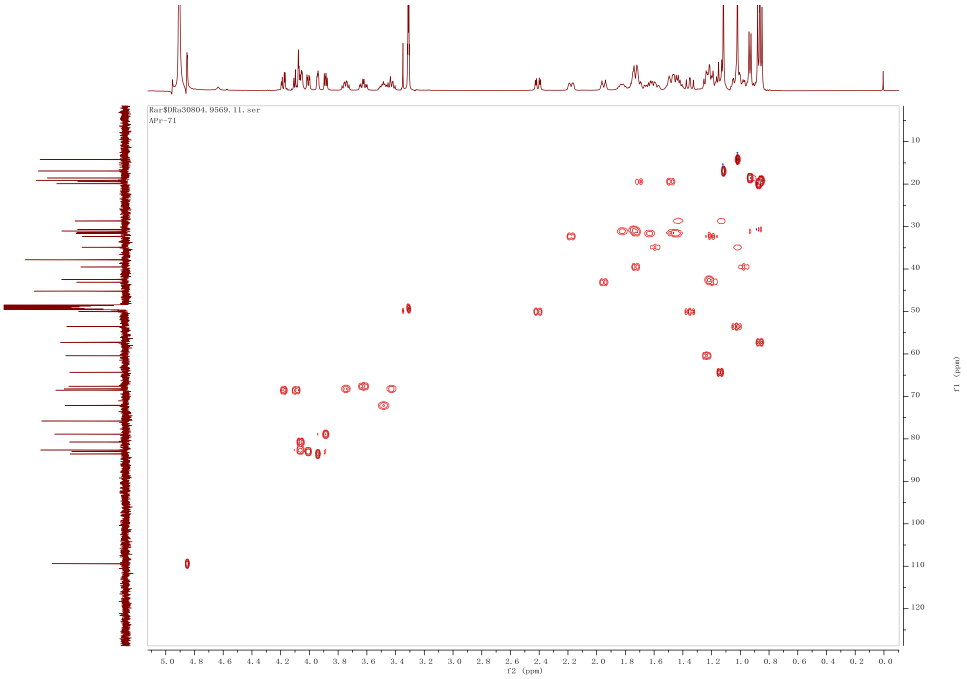

Figure S24 ^1H-^1H COSY spectrum of **3** in CD_3OD (500 MHz)

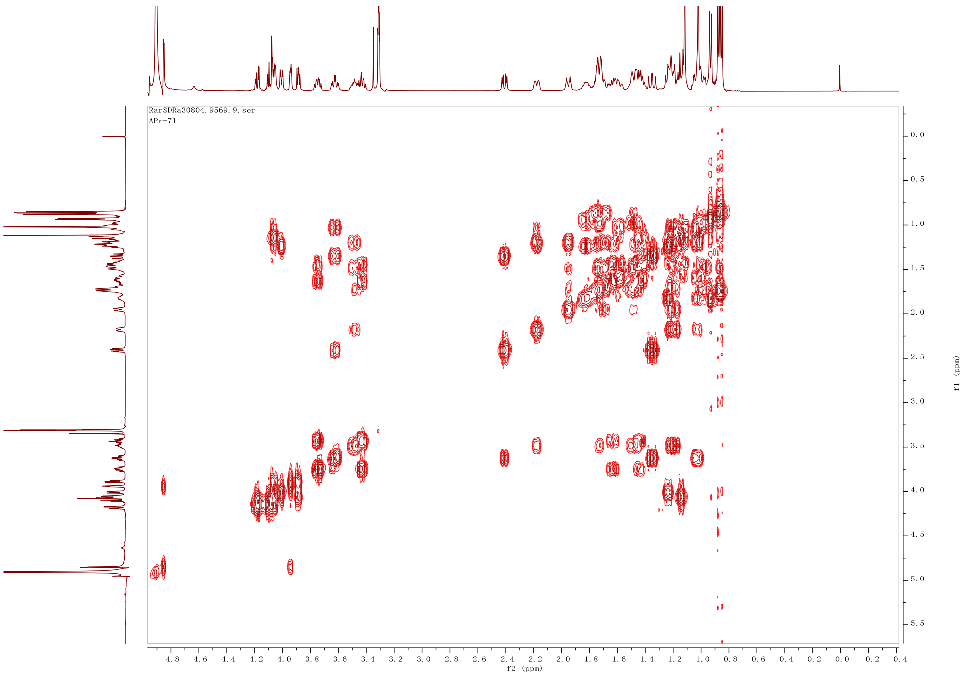

Figure S25 HMBC spectrum of **3** in CD_3OD (500 MHz)

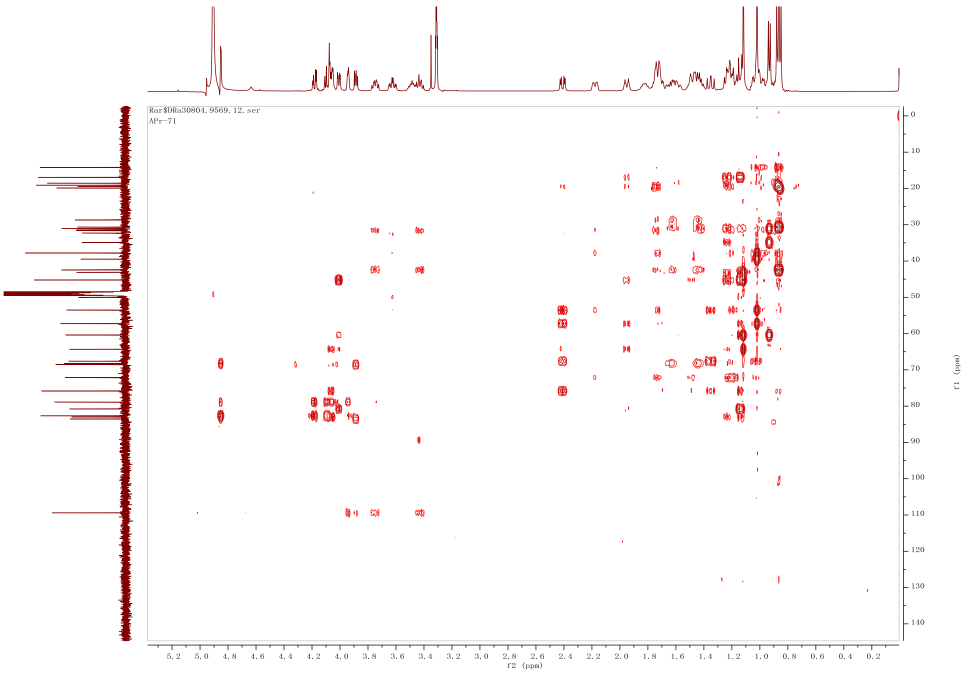

Figure S26 NOESY spectrum of **3** in CD_3OD (500 MHz)

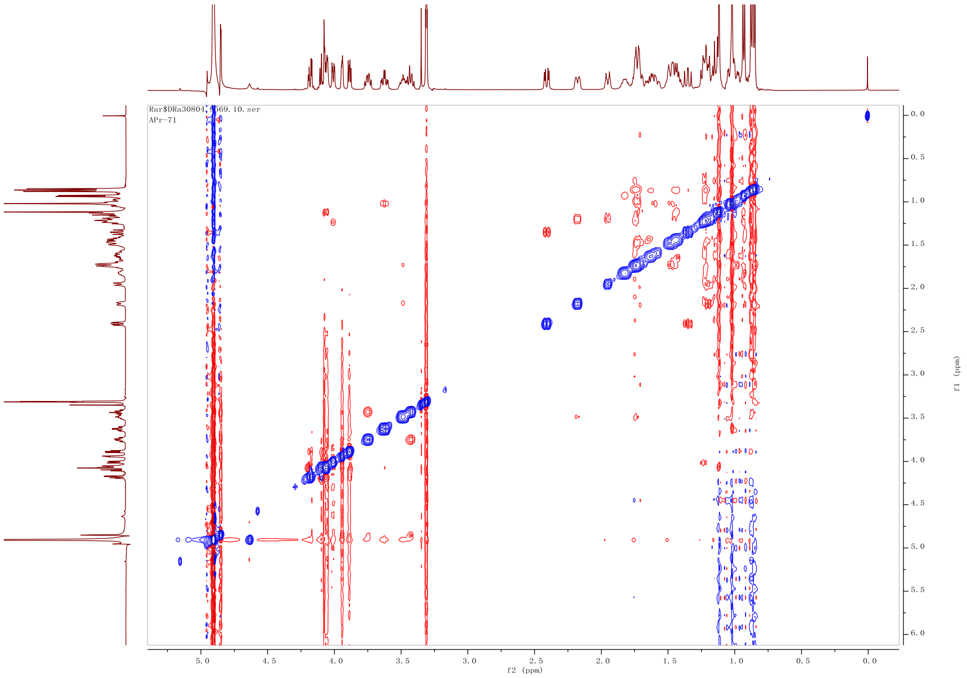

Figure S27 [α] $_D$ data of **3**

Rudolph Research Analytical

This sample was measured on an Autopol VI, Serial #91058
Manufactured by Rudolph Research Analytical, Hackettstown, NJ, USA.

Measurement Date : Wednesday, 27-DEC-2023

Set Temperature : 20.0

Time Delay : Disabled

Delay between Measurement : Disabled

n	Average	Std.Dev.	% RSD	Maximum	Minimum
5	8.54	0.50	5.85	9.09	8.18

S.No	Sample ID	Time	Result	Scale	OR °Arc	WLG.nm	Lg.mm	Conc.g/100ml	Temp.
1	APR-71	02:32:35 PM	8.18	SR	0.009	589	100.00	0.110	20.0
2	APR-71	02:32:41 PM	8.18	SR	0.009	589	100.00	0.110	20.0
3	APR-71	02:32:48 PM	9.09	SR	0.010	589	100.00	0.110	20.0
4	APR-71	02:32:55 PM	9.09	SR	0.010	589	100.00	0.110	20.0
5	APR-71	02:33:02 PM	8.18	SR	0.009	589	100.00	0.110	20.0

Figure S28 GC-MS analysis of L-arabinose and monosaccharide of **3**

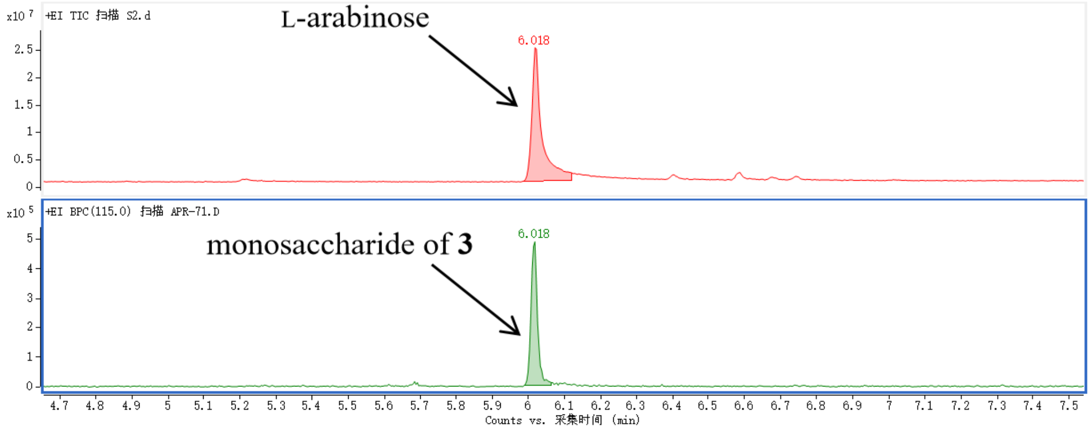

Figure S29 HR-ESI-MS and MS/MS spectra of **4**

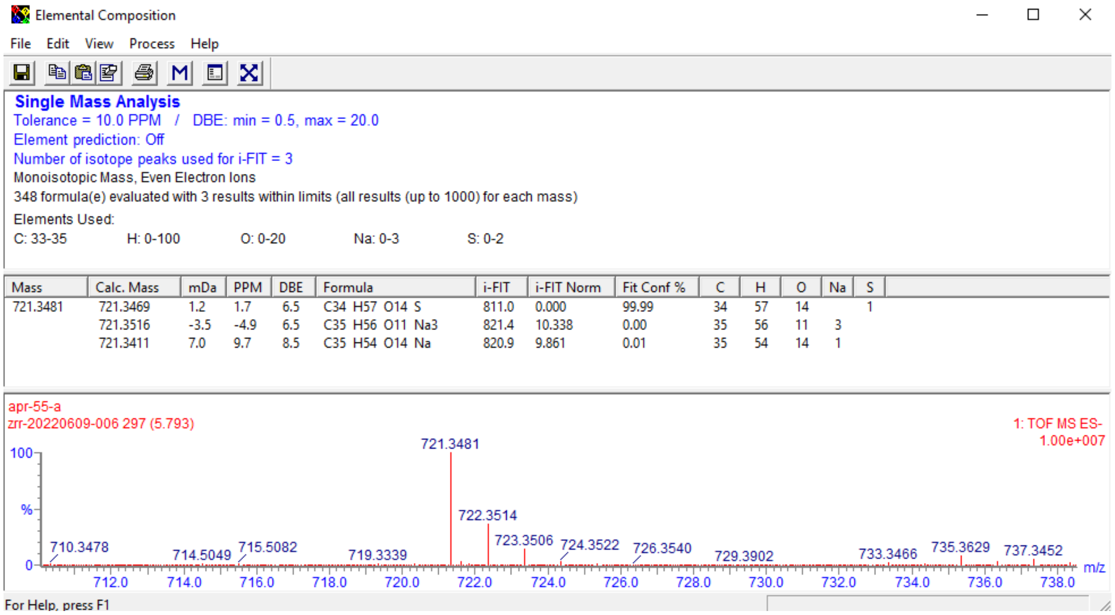

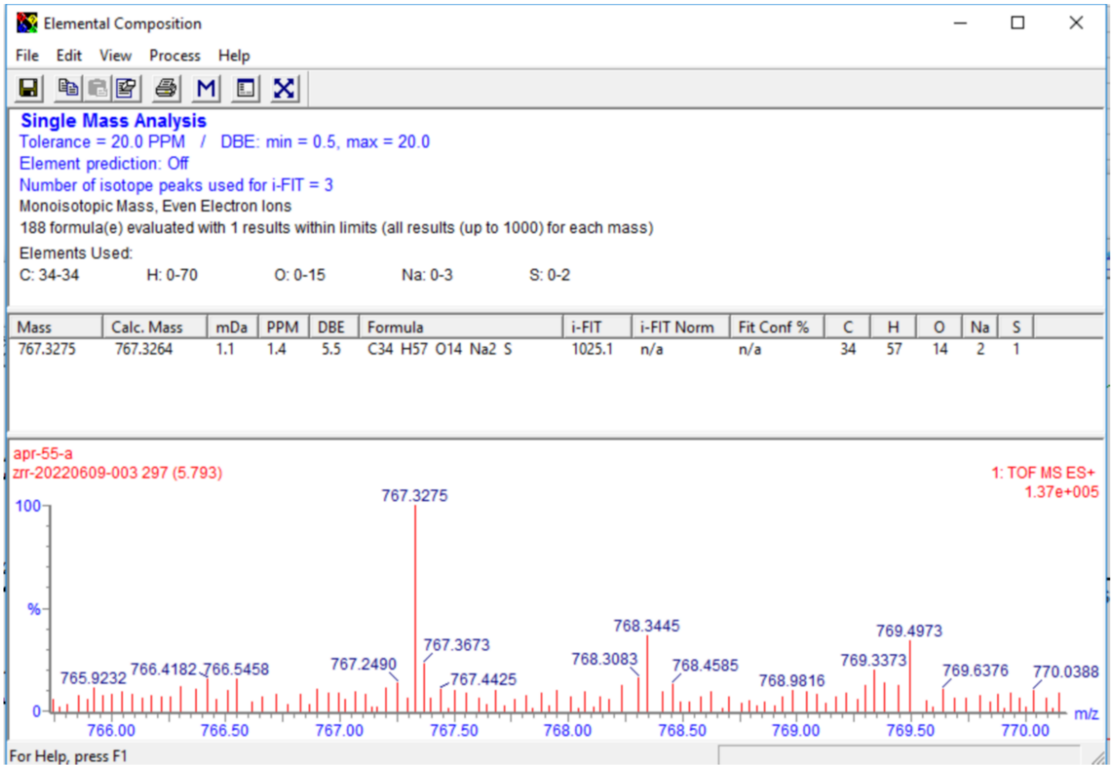

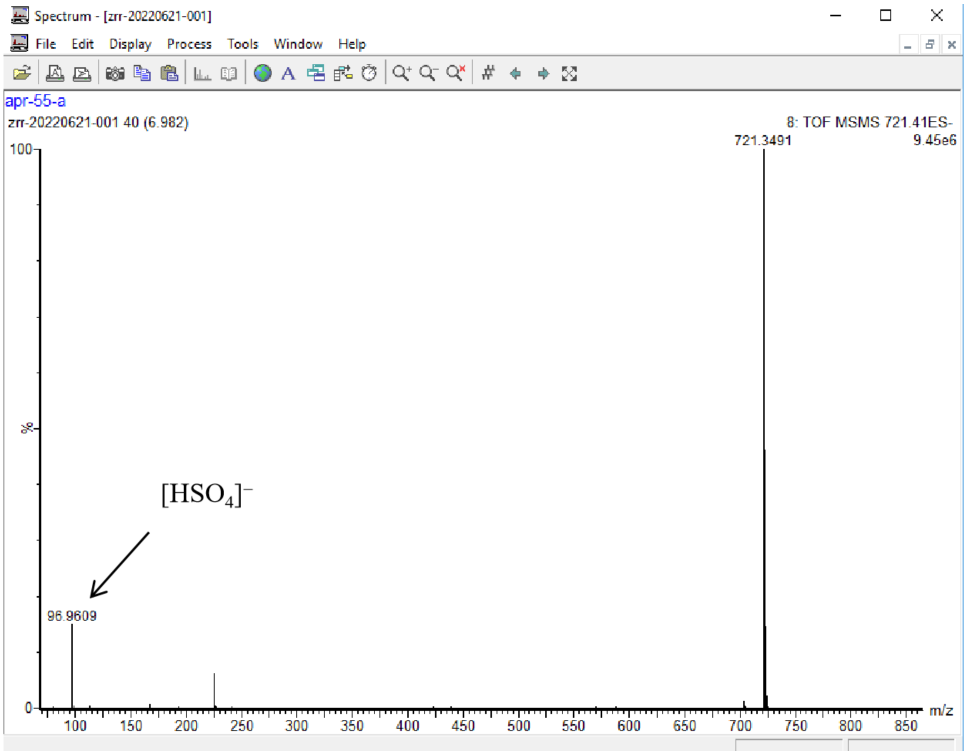
Spectrum - [zrr-20220621-001]
File Edit Display Process Tools Window Help
apr-55-a
zrr-20220621-001 40 (6.982)
8: TOF MSMS 721.41ES-
9.45e6
721.3491
100
%
0
[HSO_4]$^-$
96.9609
m/z
100 150 200 250 300 350 400 450 500 550 600 650 700 750 800 850

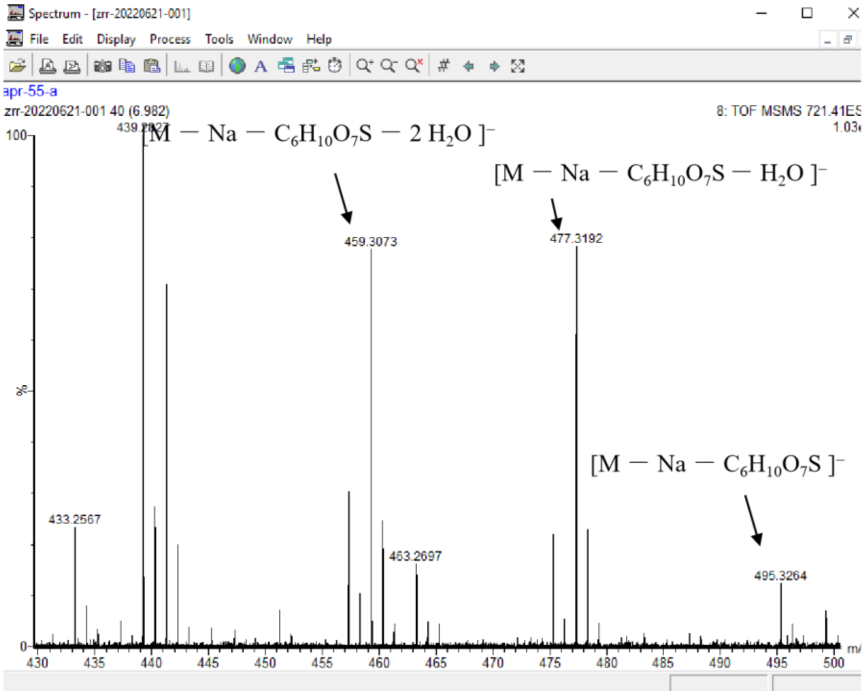
Spectrum - [zrr-20220621-001]
File Edit Display Process Tools Window Help
apr-55-a
zrr-20220621-001 40 (6.982)
8: TOF MSMS 721.41ES
[M − Na − $C_6H_{10}O_7S$ − 2 H_2O]$^-$
[M − Na − $C_6H_{10}O_7S$ − H_2O]$^-$
459.3073
477.3192
[M − Na − $C_6H_{10}O_7S$]$^-$
433.2567
463.2697
495.3264
100
%
0
430 435 440 445 450 455 460 465 470 475 480 485 490 495 500

Figure S30 ^{1}H NMR spectrum of **4** in CD_3OD (500 MHz)

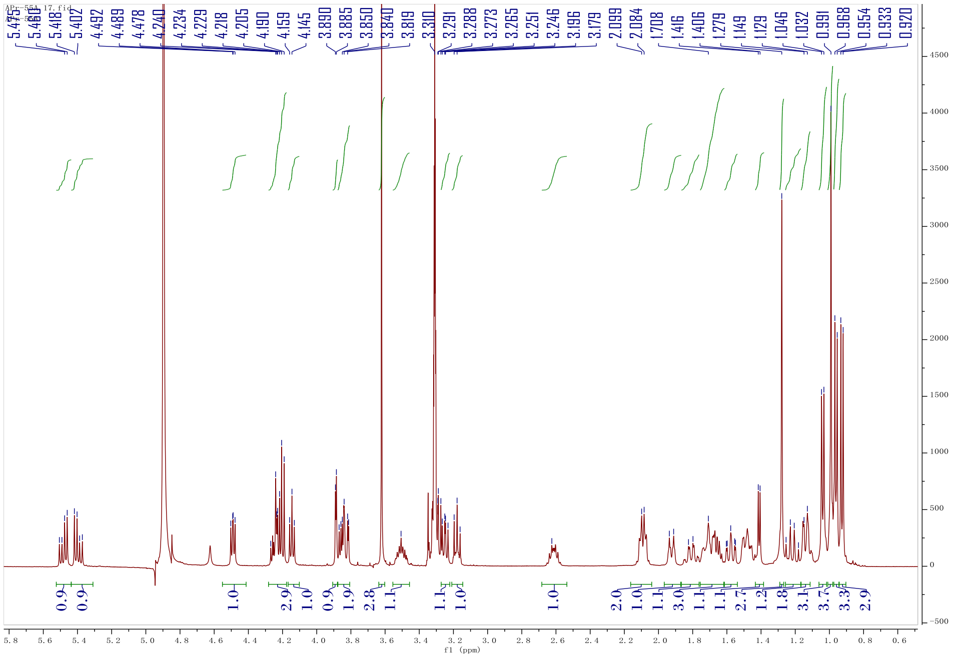

Figure S31 ^{13}C NMR spectra of **4** in CD_3OD (125 MHz)

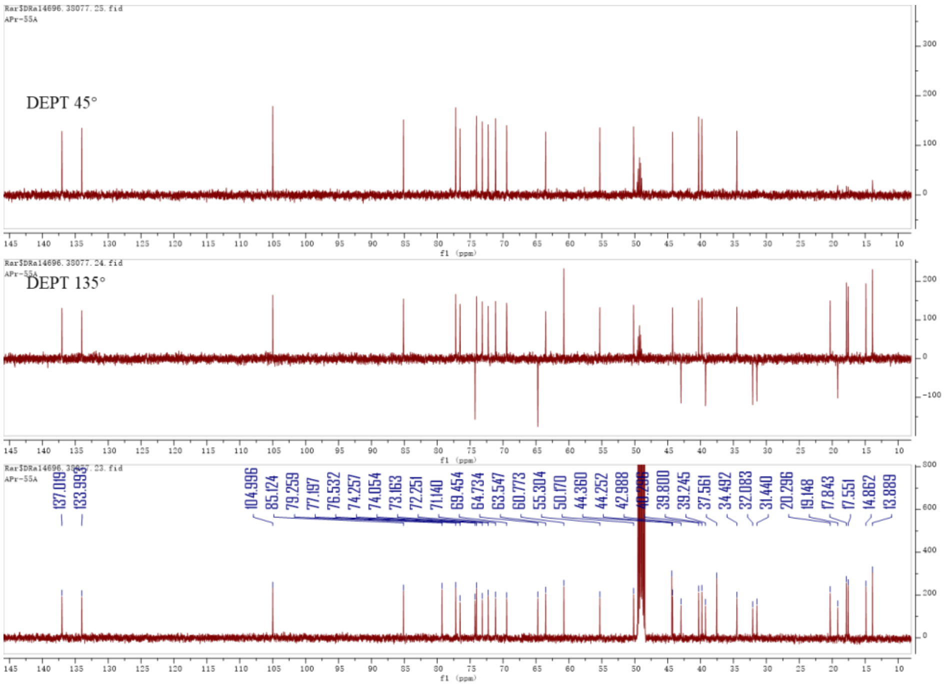

Figure S32 HSQC spectrum of **4** in CD_3OD (500 MHz)

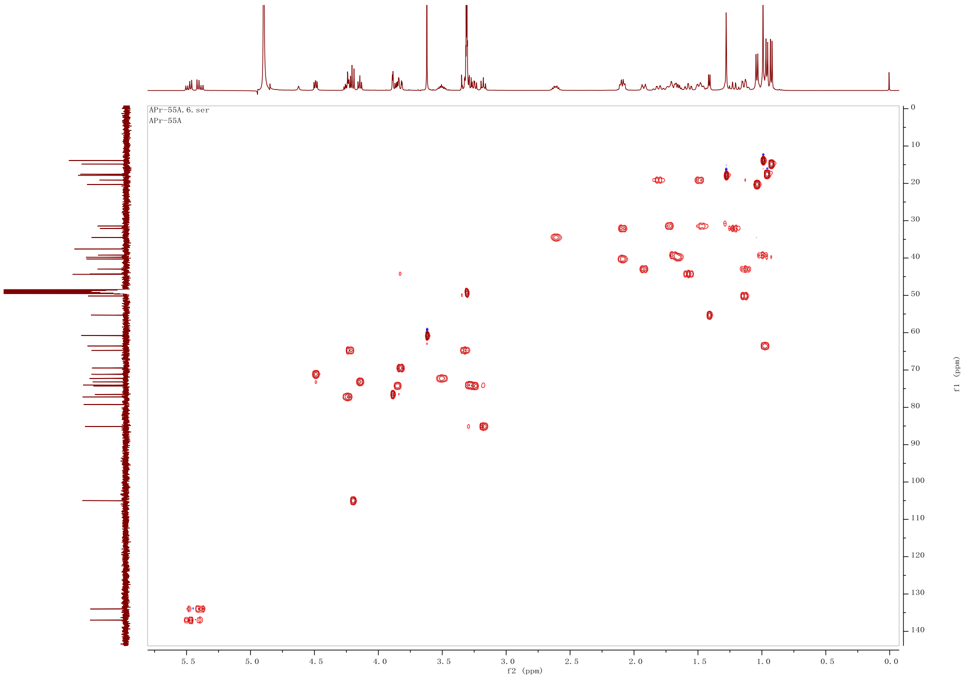

Figure S33 ^1H-^1H COSY spectrum of **4** in CD_3OD (500 MHz)

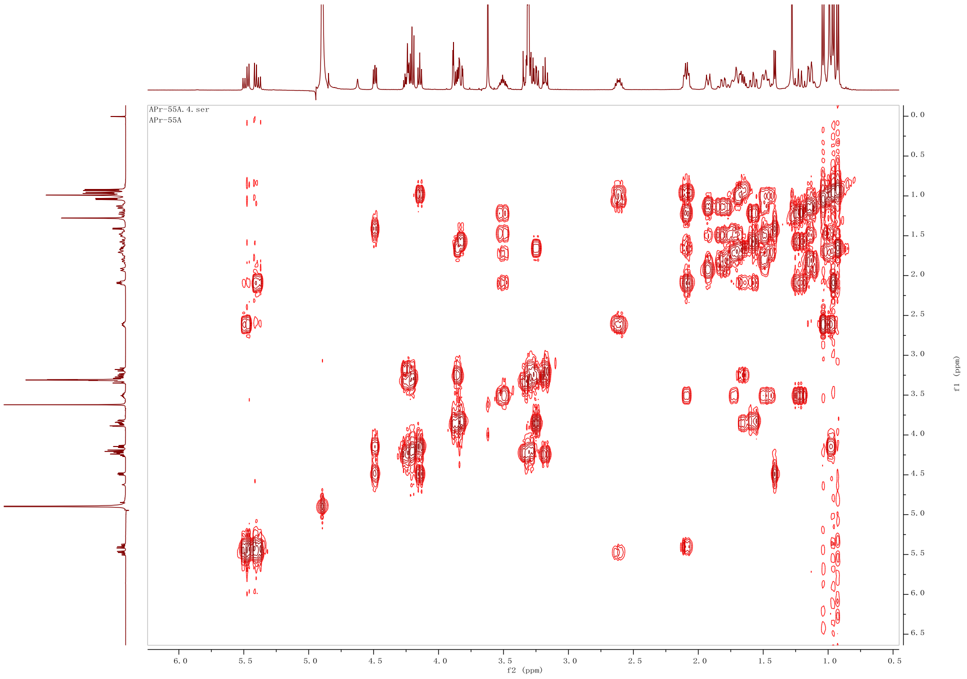

Figure S34 HMBC spectrum of **4** in CD_3OD (500 MHz)

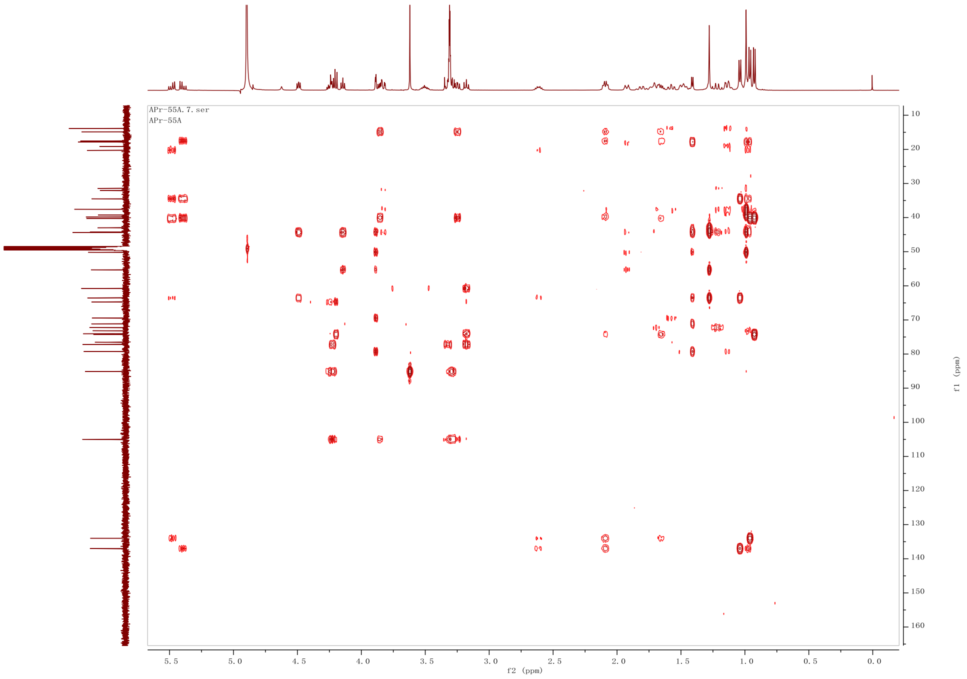

Figure S35 NOESY spectrum of **4** in CD_3OD (500 MHz)

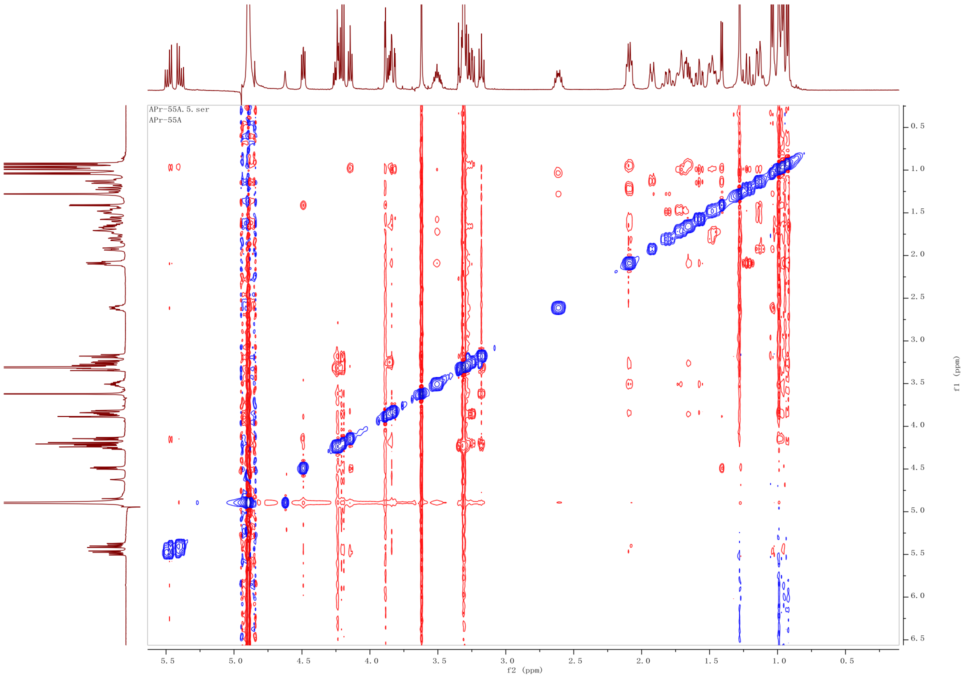

Figure S36 NOESY spectrum of **4** in $(CD_3)_2SO$ (500 MHz)

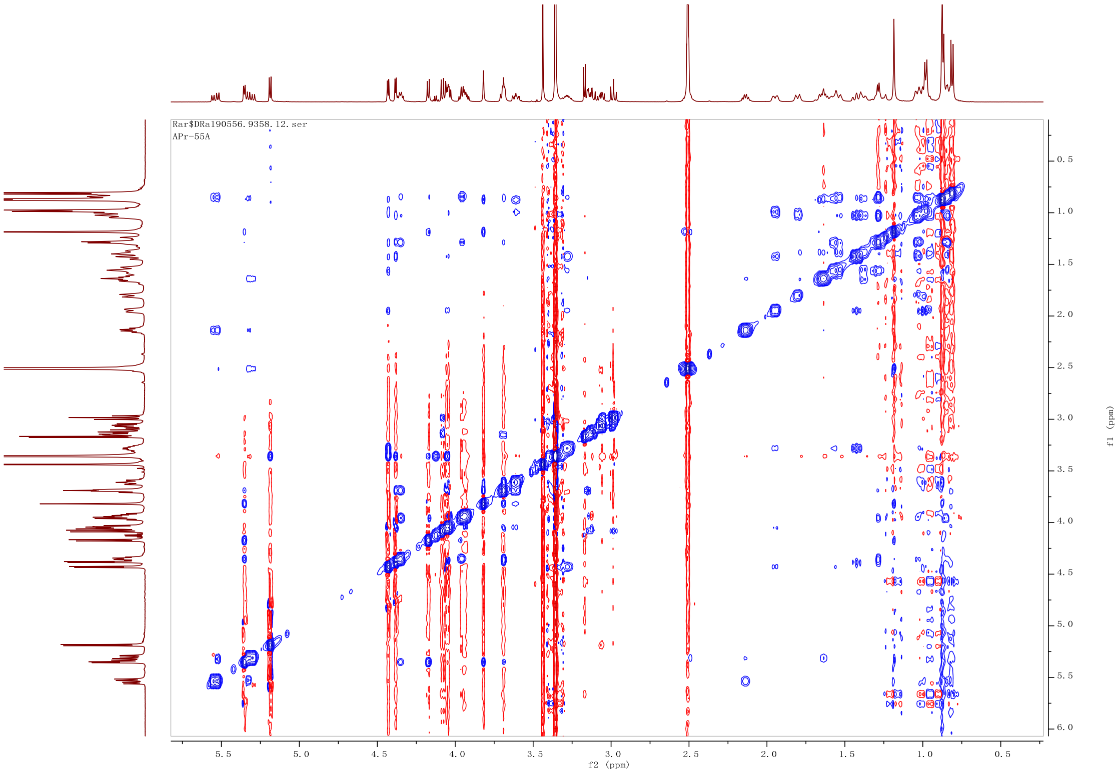

Figure S37 $[\alpha]_D$ data of **4**

Rudolph Research Analytical

This sample was measured on an Autopol VI, Serial #91058
Manufactured by Rudolph Research Analytical, Hackettstown, NJ, USA.

Measurement Date : Tuesday, 18-JUL-2023

Set Temperature : 25.0

Time Delay : Disabled

Delay between Measurement : Disabled

n	Average	Std.Dev.	% RSD	Maximum	Minimum
5	6.60	0.55	8.33	7.00	6.00

S.No	Sample ID	Time	Result	Scale	OR °Arc	WLG.nm	Lg.mm	Conc.g/100ml	Temp.
1	APR-55A	09:18:05 PM	7.00	SR	0.007	589	100.00	0.100	25.0
2	APR-55A	09:18:11 PM	6.00	SR	0.006	589	100.00	0.100	25.0
3	APR-55A	09:18:18 PM	7.00	SR	0.007	589	100.00	0.100	25.0
4	APR-55A	09:18:24 PM	6.00	SR	0.006	589	100.00	0.100	25.0
5	APR-55A	09:18:30 PM	7.00	SR	0.007	589	100.00	0.100	25.0

Figure S38 HR-ESI-MS and MS/MS spectra of **5**

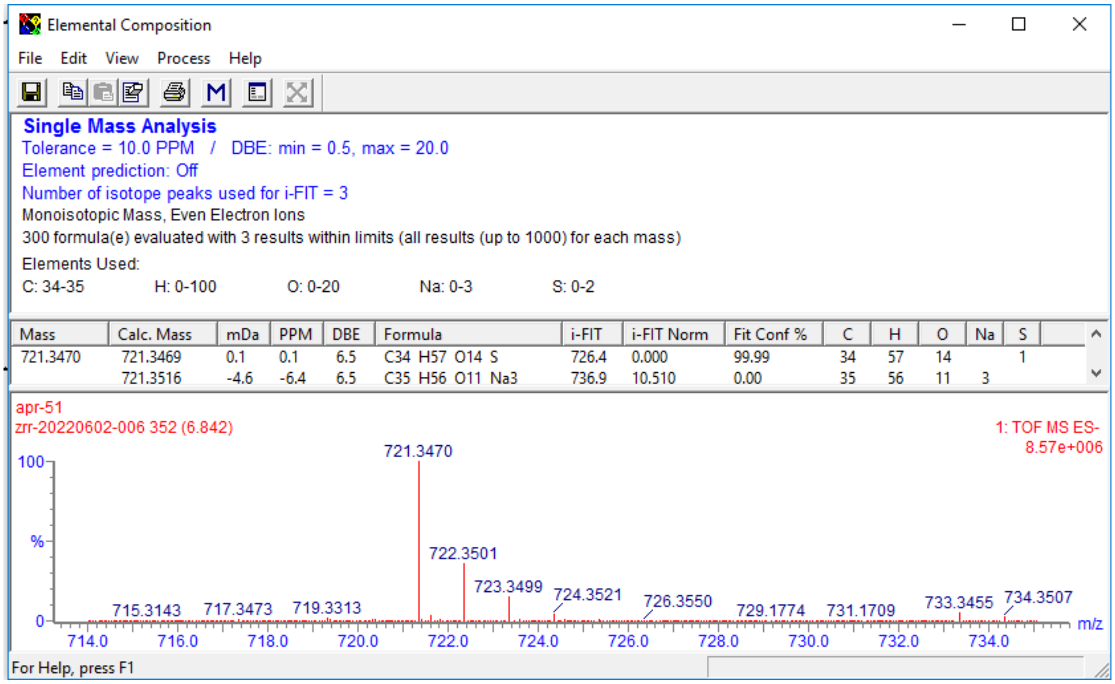

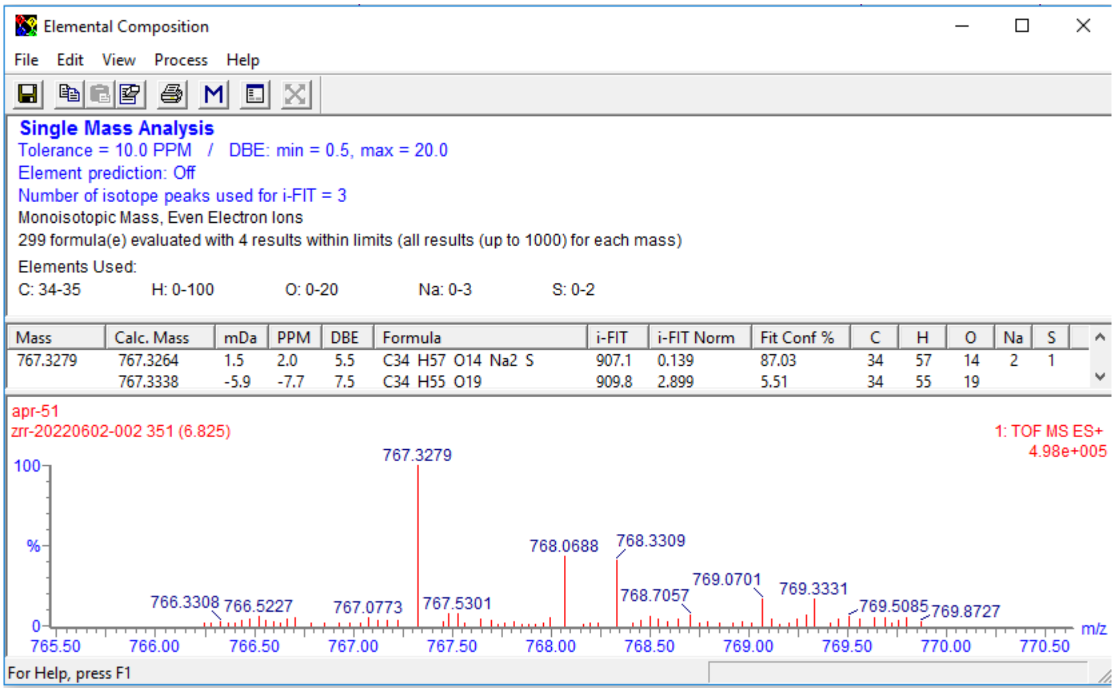

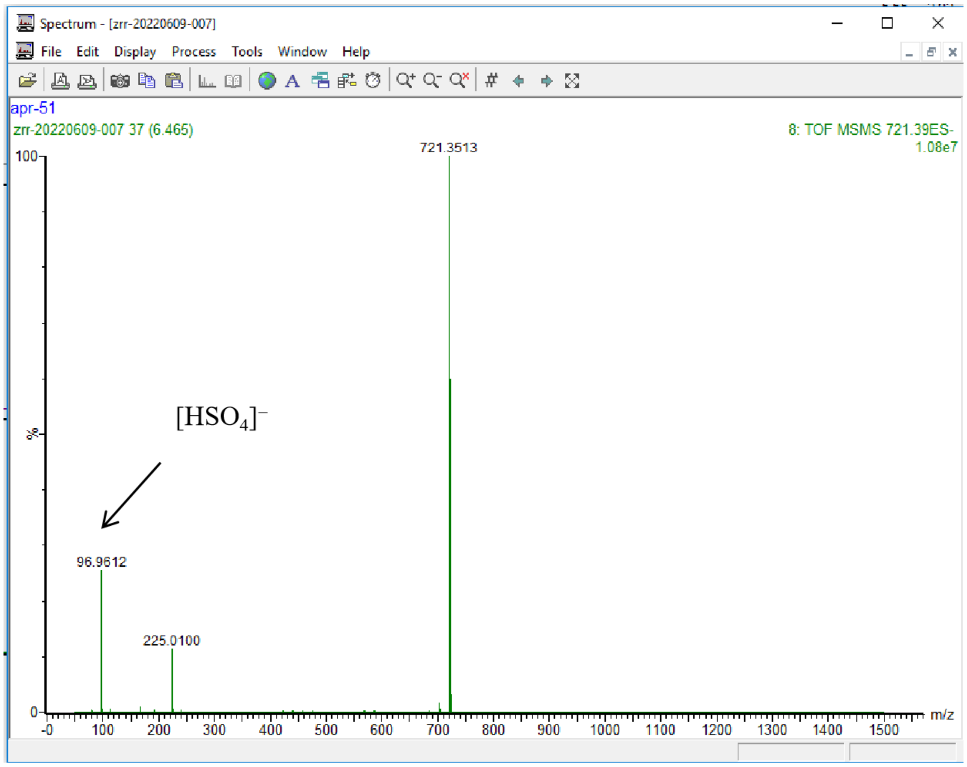
Spectrum - [zrr-20220609-007]
File Edit Display Process Tools Window Help
apr-51
zrr-20220609-007 37 (6.465)
8: TOF MSMS 721.39ES-
1.08e7
721.3513
[HSO4]⁻
96.9612
225.0100
100
%
0
m/z
-0 100 200 300 400 500 600 700 800 900 1000 1100 1200 1300 1400 1500

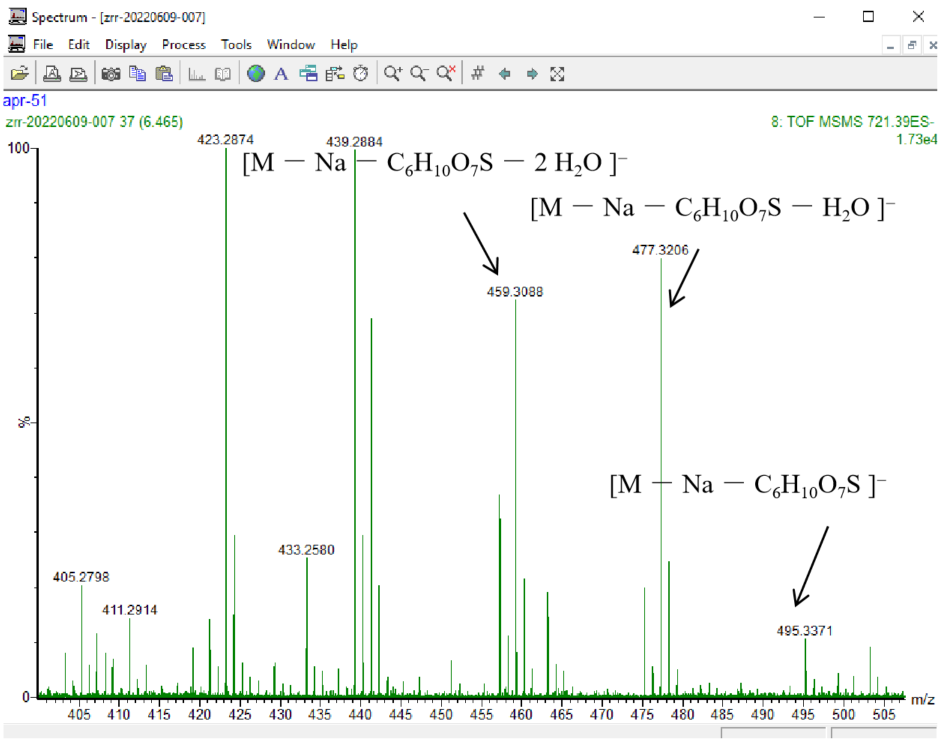
Spectrum - [zrr-20220609-007]
File Edit Display Process Tools Window Help
apr-51
zrr-20220609-007 37 (6.465)
8: TOF MSMS 721.39ES-
1.73e4
423.2874
439.2884
[M − Na − C6H10O7S − 2 H2O]⁻
[M − Na − C6H10O7S − H2O]⁻
477.3206
459.3088
[M − Na − C6H10O7S]⁻
433.2580
405.2798
411.2914
495.3371
100
%
0
m/z
405 410 415 420 425 430 435 440 445 450 455 460 465 470 475 480 485 490 495 500 505

Figure S39 ^{1}H NMR spectrum of **5** in CD_3OD (500 MHz)

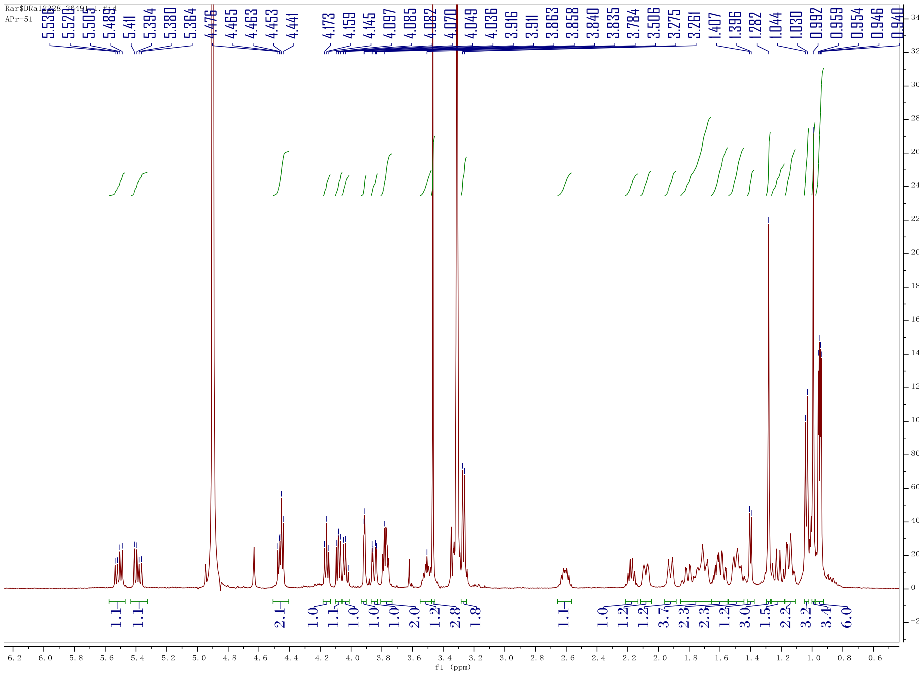

Figure S40 ^{13}C NMR spectra of **5** in CD_3OD (125 MHz)

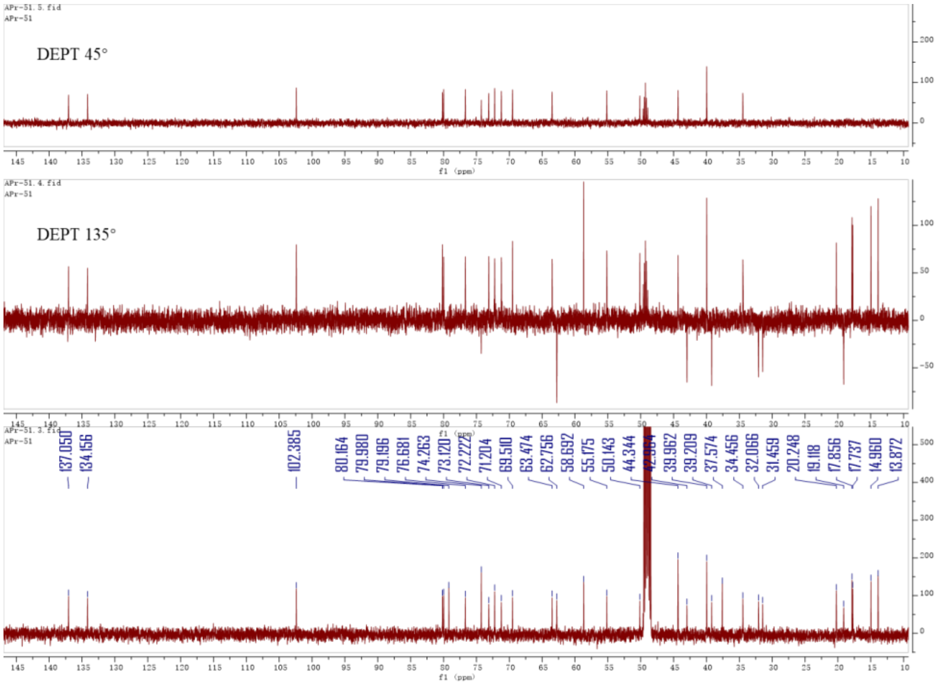

Figure S41 HSQC spectrum of **5** in CD_3OD (500 MHz)

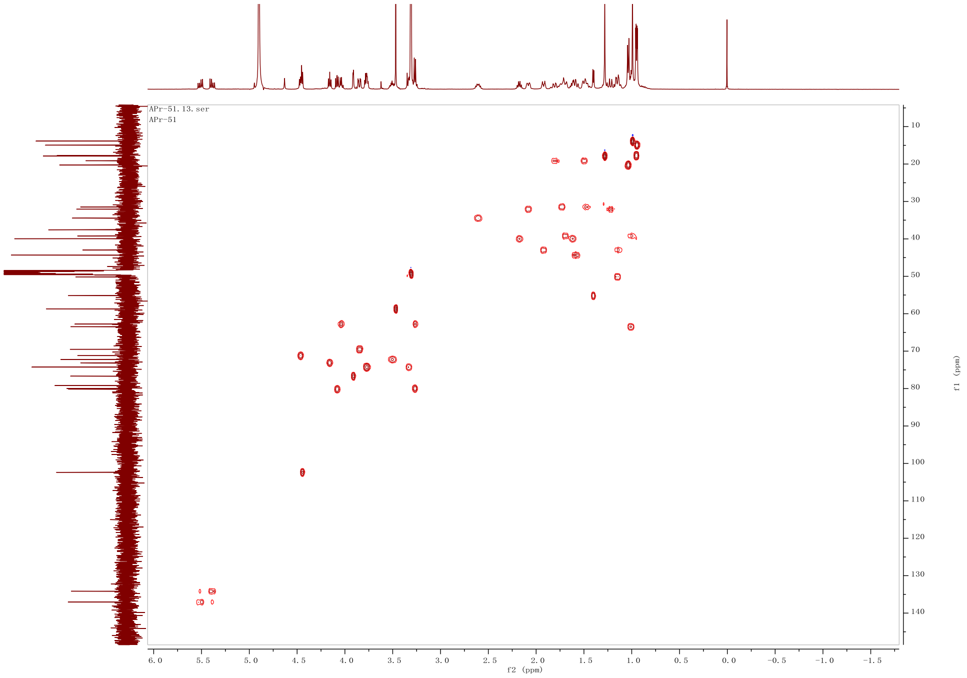

Figure S42 ^1H-^1H COSY spectrum of **5** in CD_3OD (500 MHz)

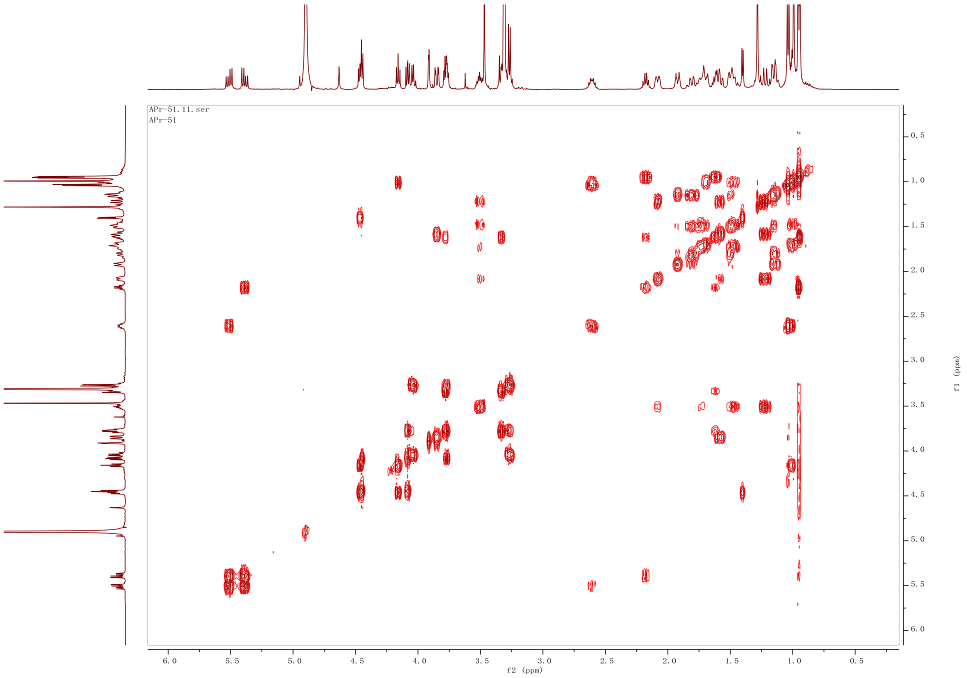

Figure S43 HMBC spectrum of **5** in CD_3OD (500 MHz)

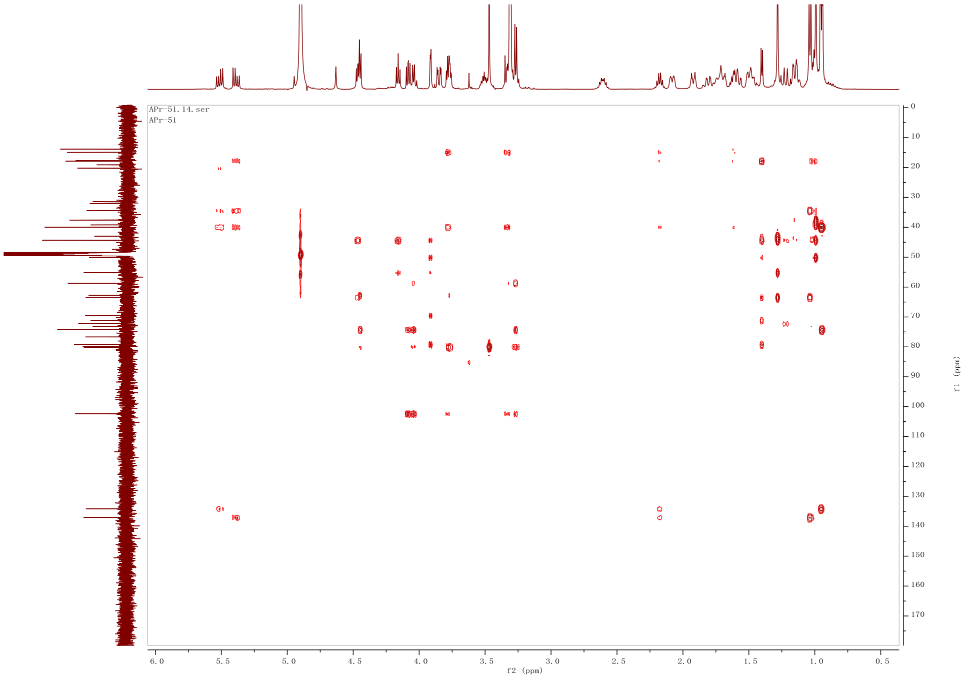

Figure S44 NOESY spectrum of **5** in CD_3OD (500 MHz)

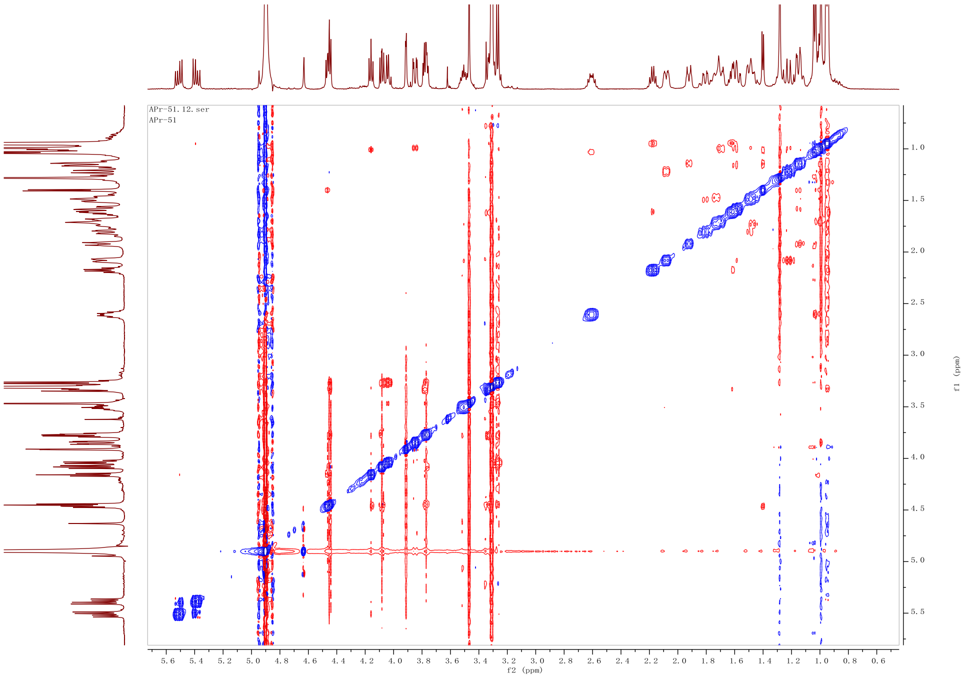

Figure S45 [α] $_D$ data of **5**

Rudolph Research Analytical

This sample was measured on an Autopol VI, Serial #91058
Manufactured by Rudolph Research Analytical, Hackettstown, NJ, USA.

Measurement Date : Tuesday, 18-JUL-2023

Set Temperature : 25.0

Time Delay : Disabled

Delay between Measurement : Disabled

n	Average	Std.Dev.	% RSD	Maximum	Minimum
5	6.77	0.34	5.02	6.92	6.15

S.No	Sample ID	Time	Result	Scale	OR °Arc	WLG.nm	Lg.mm	Conc.g/100ml	Temp.
1	APR-51	09:02:57 PM	6.92	SR	0.009	589	100.00	0.130	25.0
2	APR-51	09:03:03 PM	6.92	SR	0.009	589	100.00	0.130	25.0
3	APR-51	09:03:10 PM	6.92	SR	0.009	589	100.00	0.130	25.0
4	APR-51	09:03:16 PM	6.92	SR	0.009	589	100.00	0.130	25.0
5	APR-51	09:03:23 PM	6.15	SR	0.008	589	100.00	0.130	25.0

Figure S46 HR-ESI-MS and MS/MS spectra of **6**

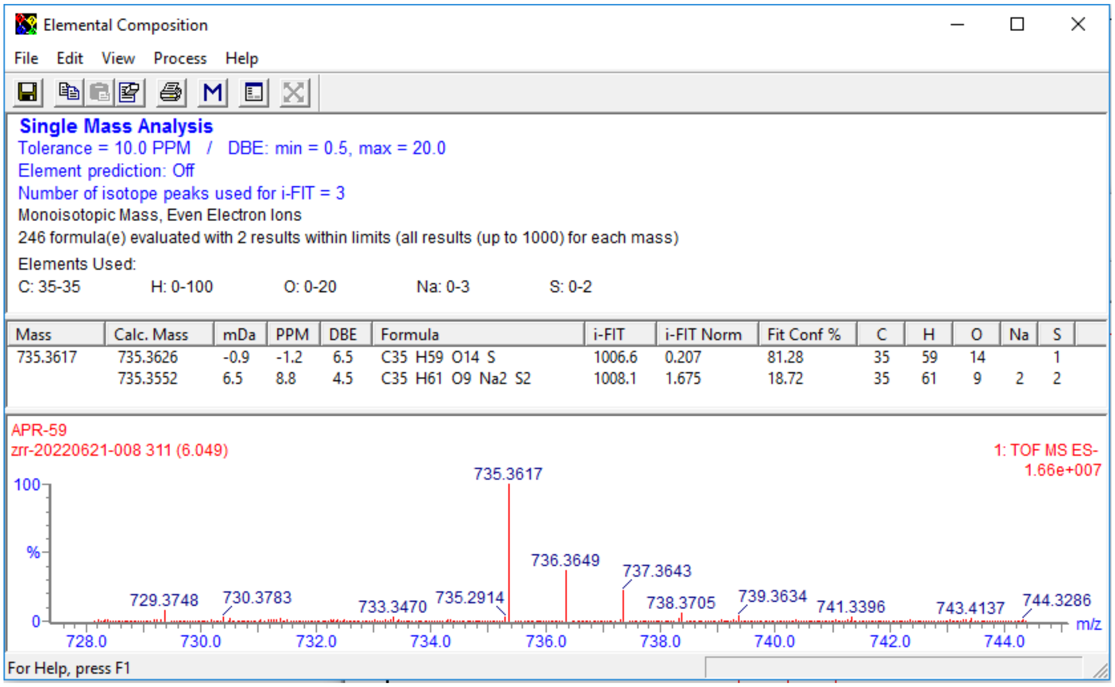

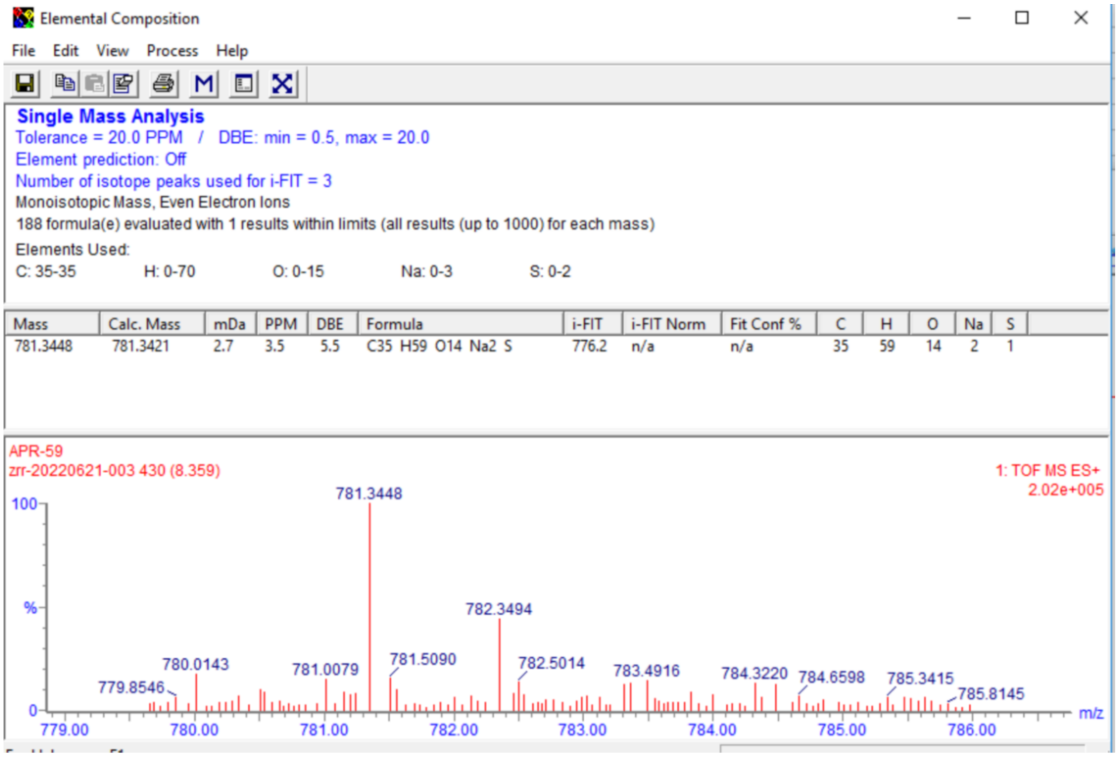

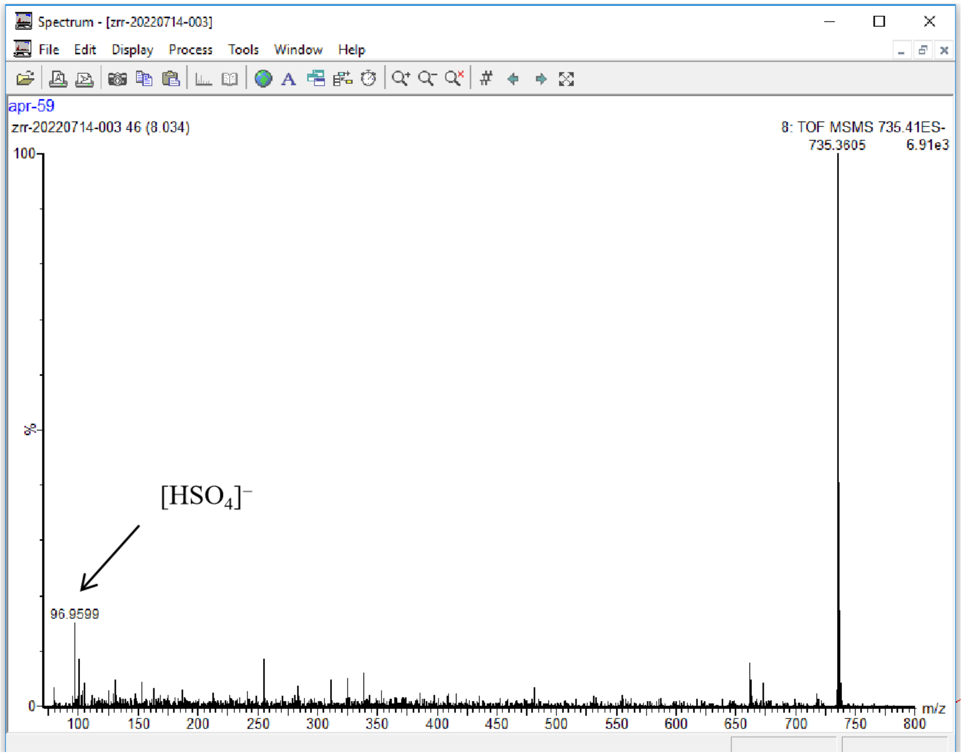
Spectrum - [zrr-20220714-003]
File Edit Display Process Tools Window Help
apr-59
zrr-20220714-003 46 (8.034)
8: TOF MSMS 735.41ES-
735.3605
6.91e3
100
%
0
$[HSO_4]^-$
96.9599
m/z
100 150 200 250 300 350 400 450 500 550 600 650 700 750 800

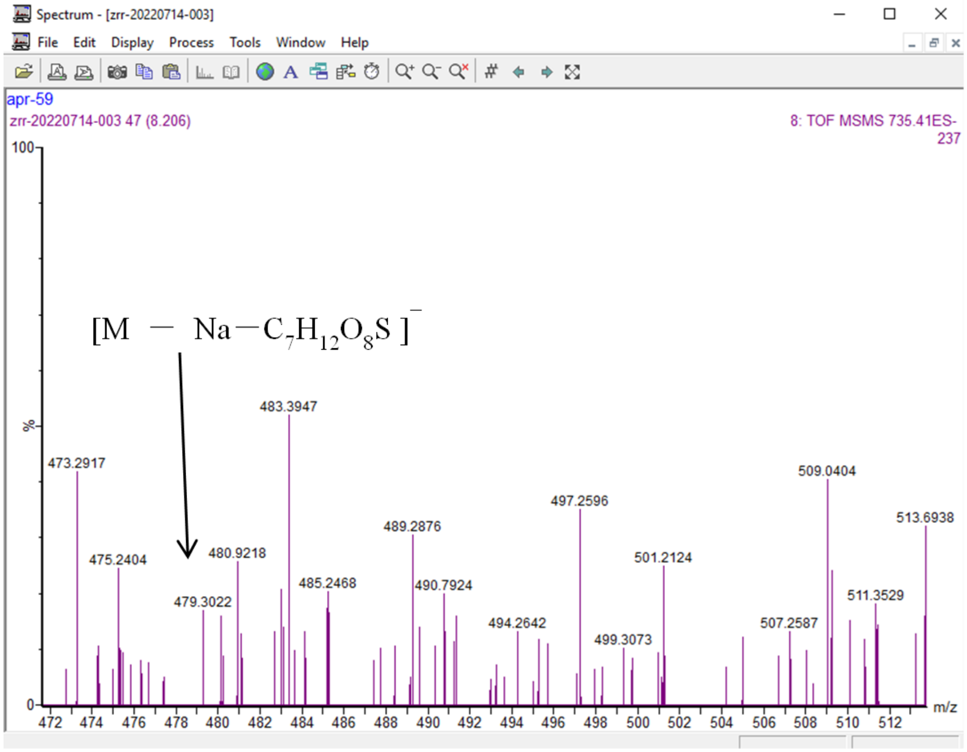
Spectrum - [zrr-20220714-003]
File Edit Display Process Tools Window Help
apr-59
zrr-20220714-003 47 (8.206)
8: TOF MSMS 735.41ES-
237
100
%
0
$[M - Na - C_7H_{12}O_8S]^-$
483.3947
473.2917
509.0404
497.2596
513.6938
489.2876
475.2404
480.9218
501.2124
485.2468
490.7924
479.3022
511.3529
494.2642
507.2587
499.3073
m/z
472 474 476 478 480 482 484 486 488 490 492 494 496 498 500 502 504 506 508 510 512

Figure S47 ^{1}H NMR spectrum of **6** in CD_3OD (500 MHz)

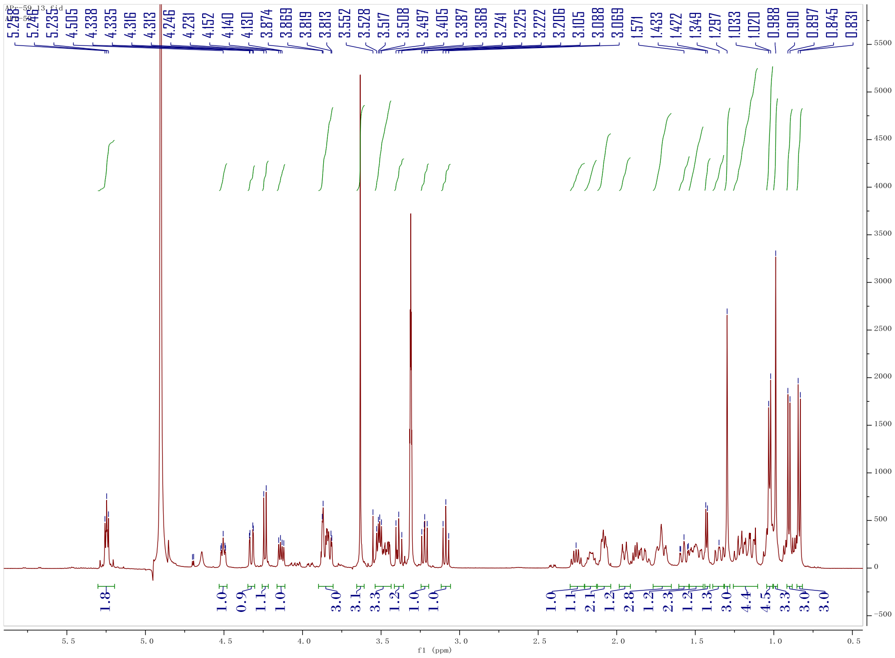

Figure S48 ^{13}C NMR spectra of **6** in CD_3OD (125 MHz)

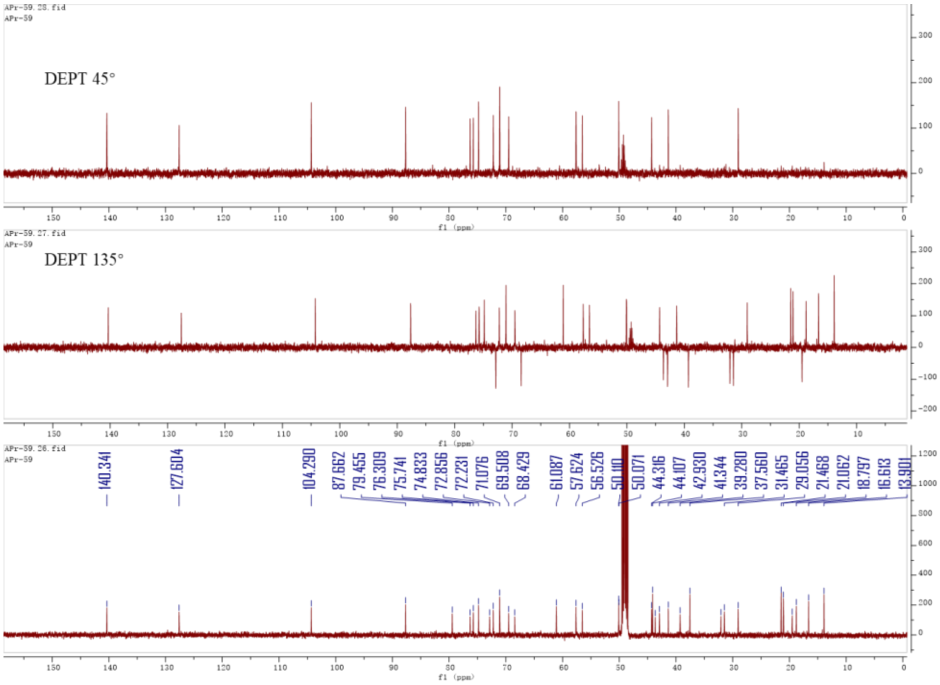

Figure S49 HSQC spectrum of **6** in CD_3OD (500 MHz)

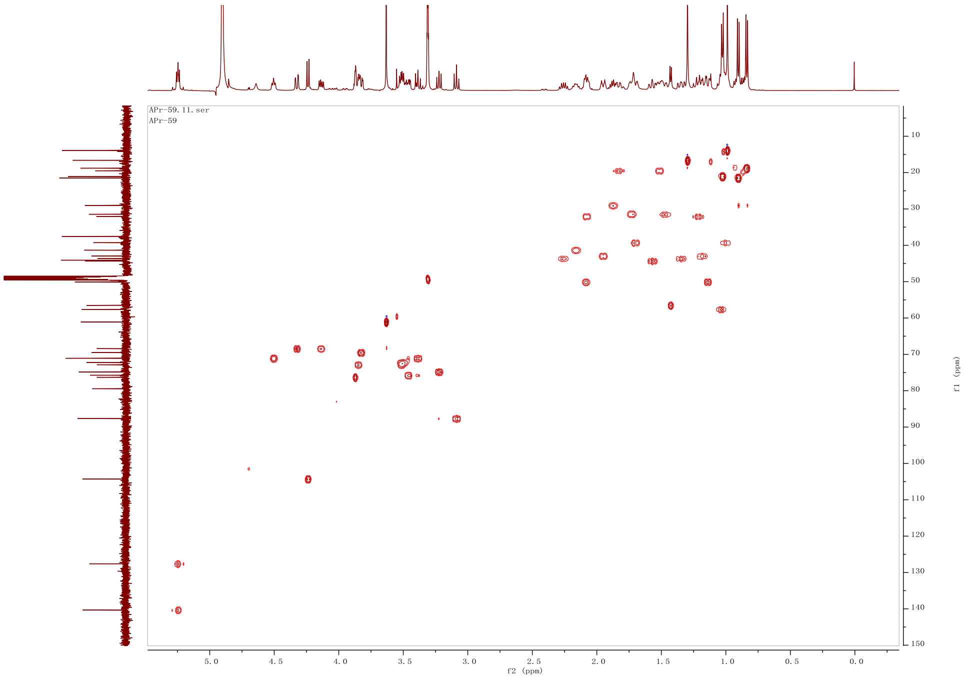

Figure S50 ^{1}H-^{1}H COSY spectrum of **6** in CD_3OD (500 MHz)

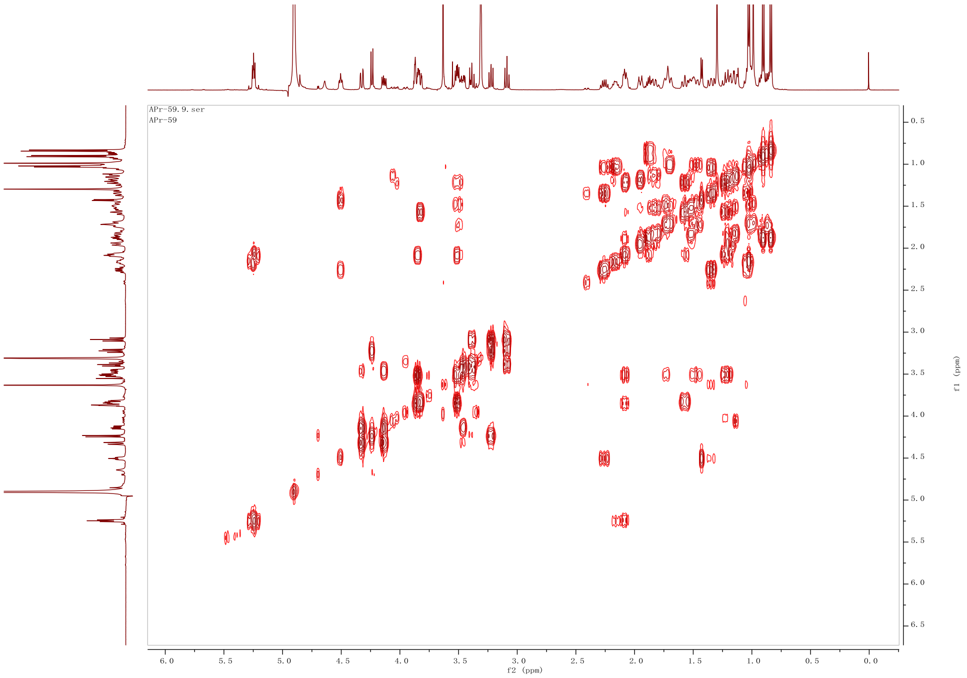

Figure S51 HMBC spectrum of **6** in CD_3OD (500 MHz)

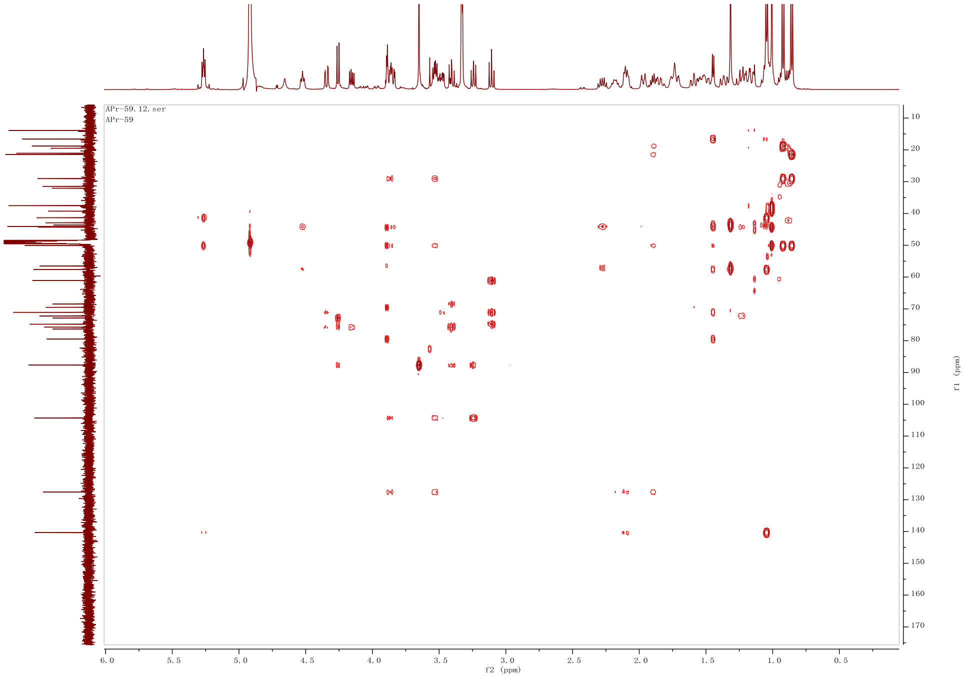

Figure S52 NOESY spectrum of **6** in CD_3OD (500 MHz)

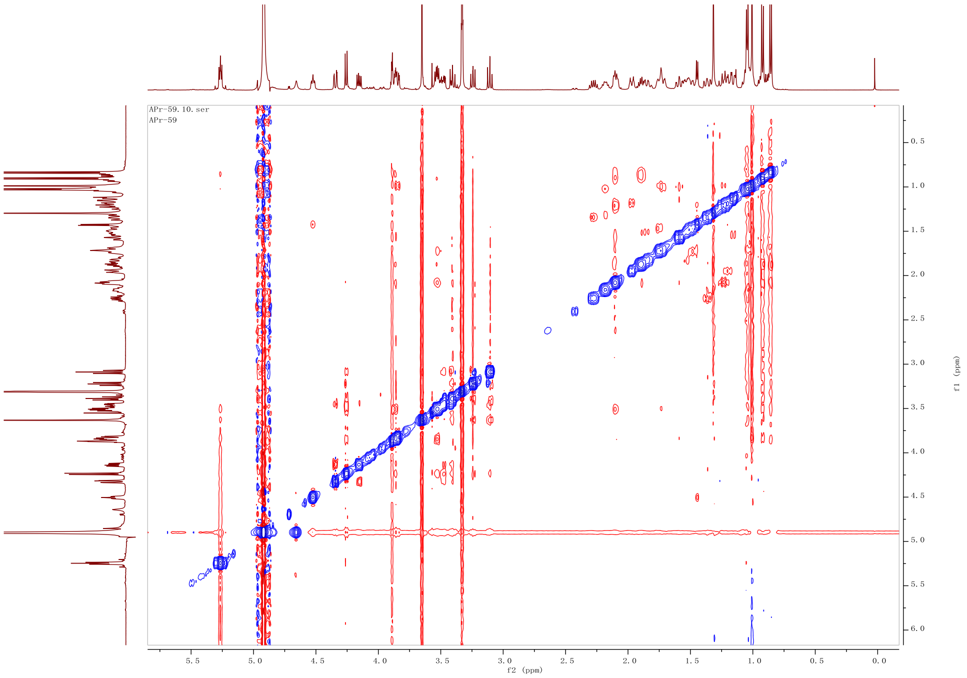

Figure S53 NOESY spectrum of **6** in $(CD_3)_2SO$ (500 MHz)

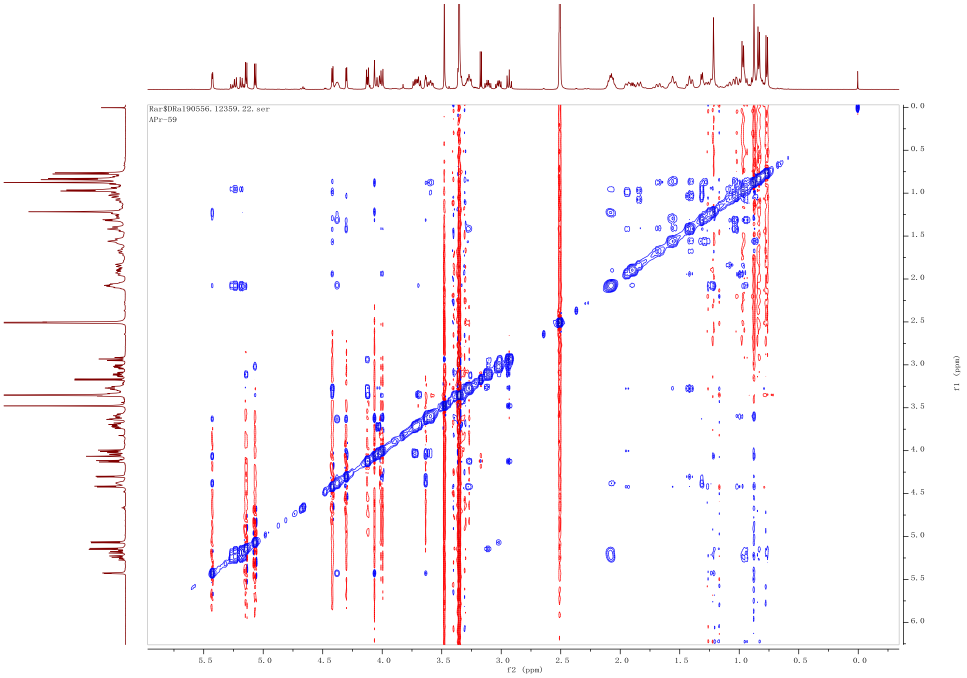

Figure S54 [α] D data of **6**

Rudolph Research Analytical

This sample was measured on an Autopol VI, Serial #91058
Manufactured by Rudolph Research Analytical, Hackettstown, NJ, USA.

Measurement Date : Tuesday, 18-JUL-2023

Set Temperature : 25.0

Time Delay : Disabled

Delay between Measurement : Disabled

n	Average	Std.Dev.	% RSD	Maximum	Minimum
5	-4.55	0.00	0.00	-4.55	-4.55

S.No	Sample ID	Time	Result	Scale	OR °Arc	WLG.nm	Lg.mm	Conc.g/100ml	Temp.
1	APR-59	09:25:06 PM	-4.55	SR	-0.005	589	100.00	0.110	25.0
2	APR-59	09:25:13 PM	-4.55	SR	-0.005	589	100.00	0.110	25.0
3	APR-59	09:25:19 PM	-4.55	SR	-0.005	589	100.00	0.110	25.0
4	APR-59	09:25:25 PM	-4.55	SR	-0.005	589	100.00	0.110	25.0
5	APR-59	09:25:32 PM	-4.55	SR	-0.005	589	100.00	0.110	25.0

Figure S55 GC-MS analysis of D-3-*O*-methyl-glucose and monosaccharide of **6**

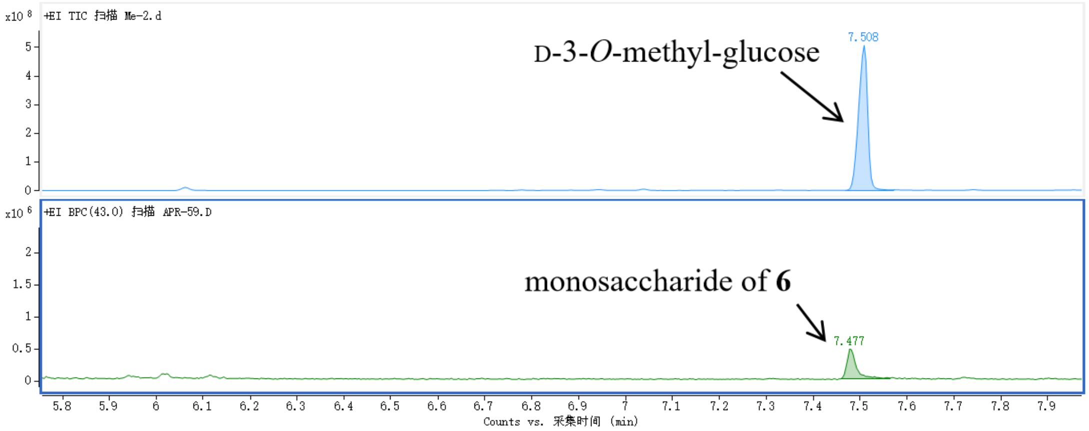

Figure S56 HR-ESI-MS and MS/MS spectra of **7**

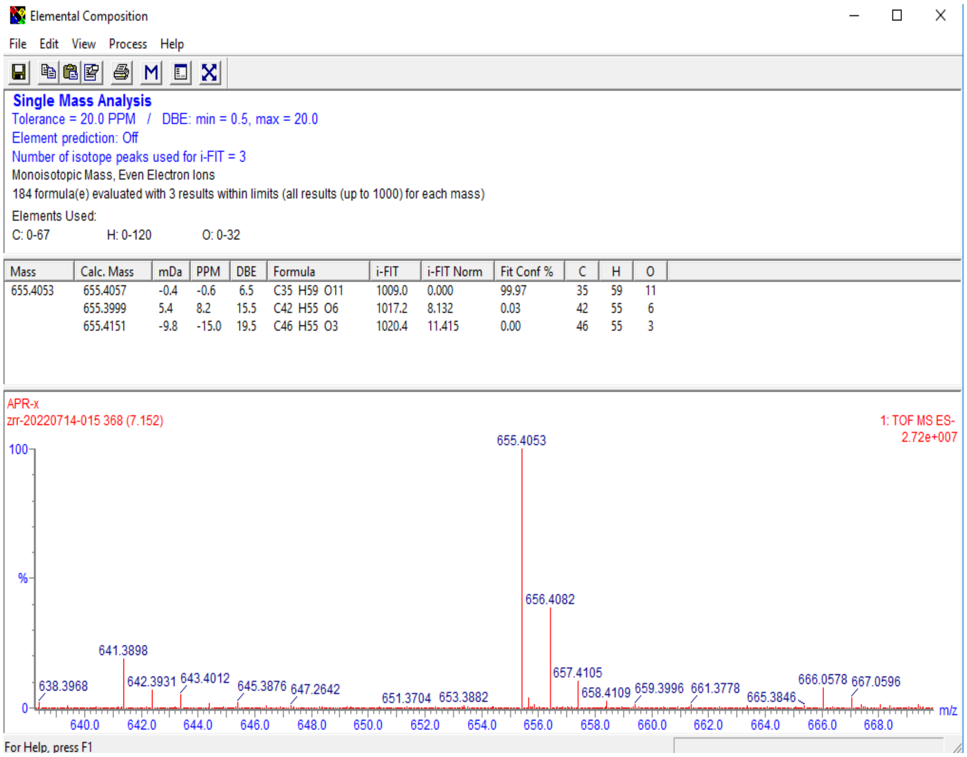

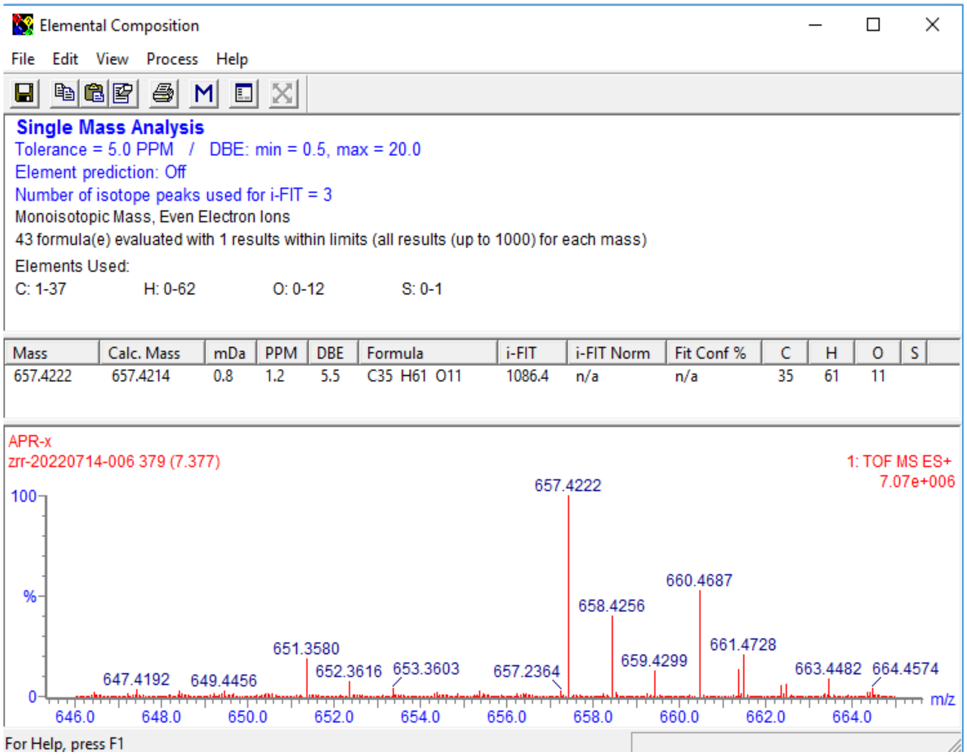

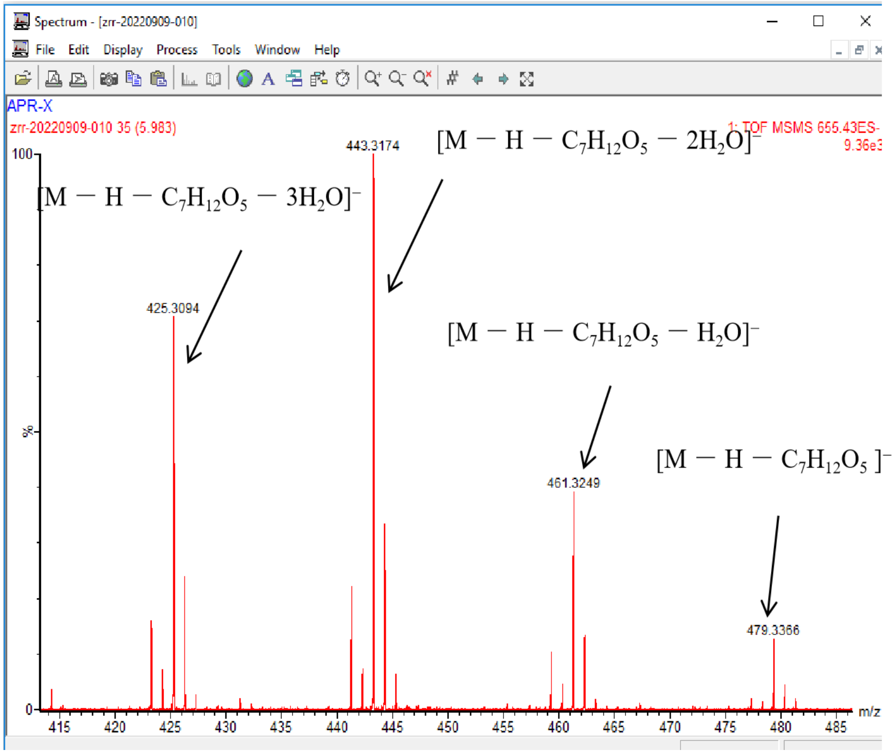
Spectrum - [zrr-20220909-010]
File Edit Display Process Tools Window Help
APR-X
zrr-20220909-010 35 (5.983)
1: TOF MSMS 655.43ES-
9.36e3
[M − H − C7H12O5 − 2H2O]−
[M − H − C7H12O5 − 3H2O]−
[M − H − C7H12O5 − H2O]−
[M − H − C7H12O5]−
443.3174
425.3094
461.3249
479.3366
100
%
0
m/z
415
420
425
430
435
440
445
450
455
460
465
470
475
480
485

Figure S57 ^{1}H NMR spectrum of **7** in CD_3OD (500 MHz)

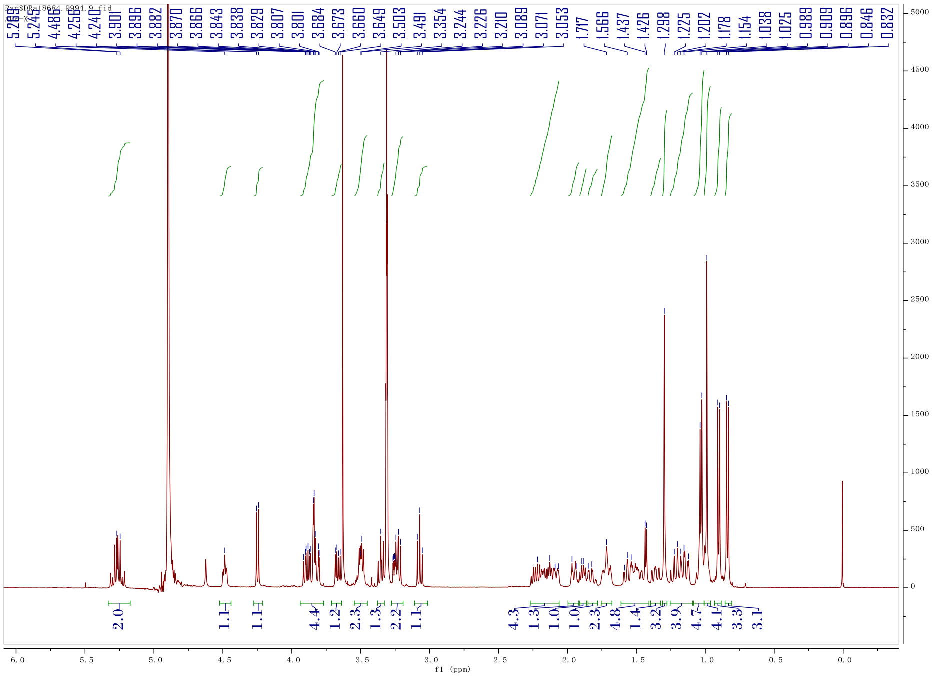

Figure S58 ^{13}C NMR spectra of **7** in CD_3OD (125 MHz)

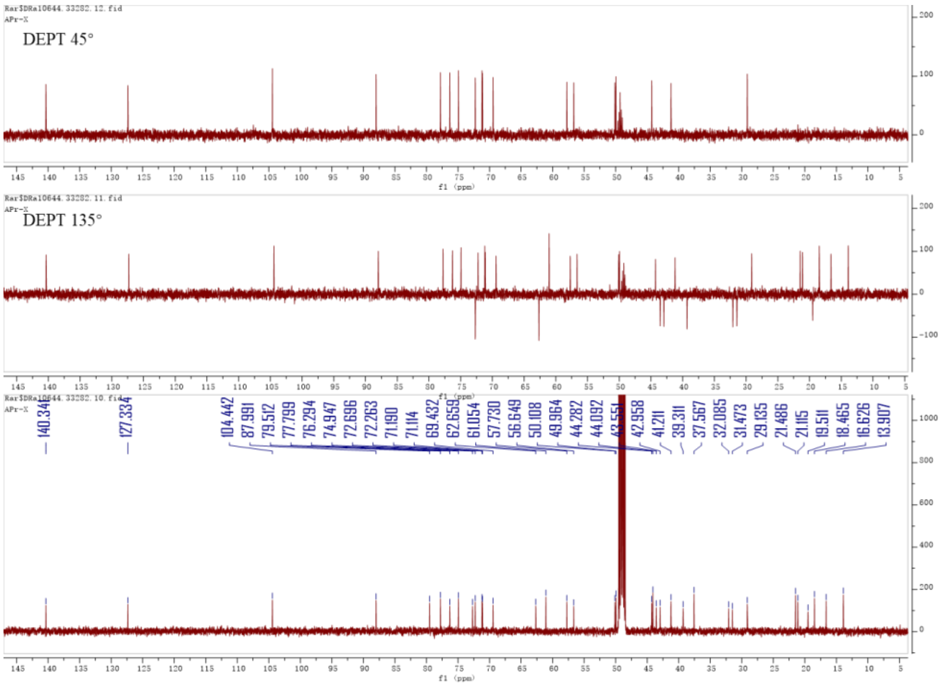

Figure S59 HSQC spectrum of **7** in CD_3OD (500 MHz)

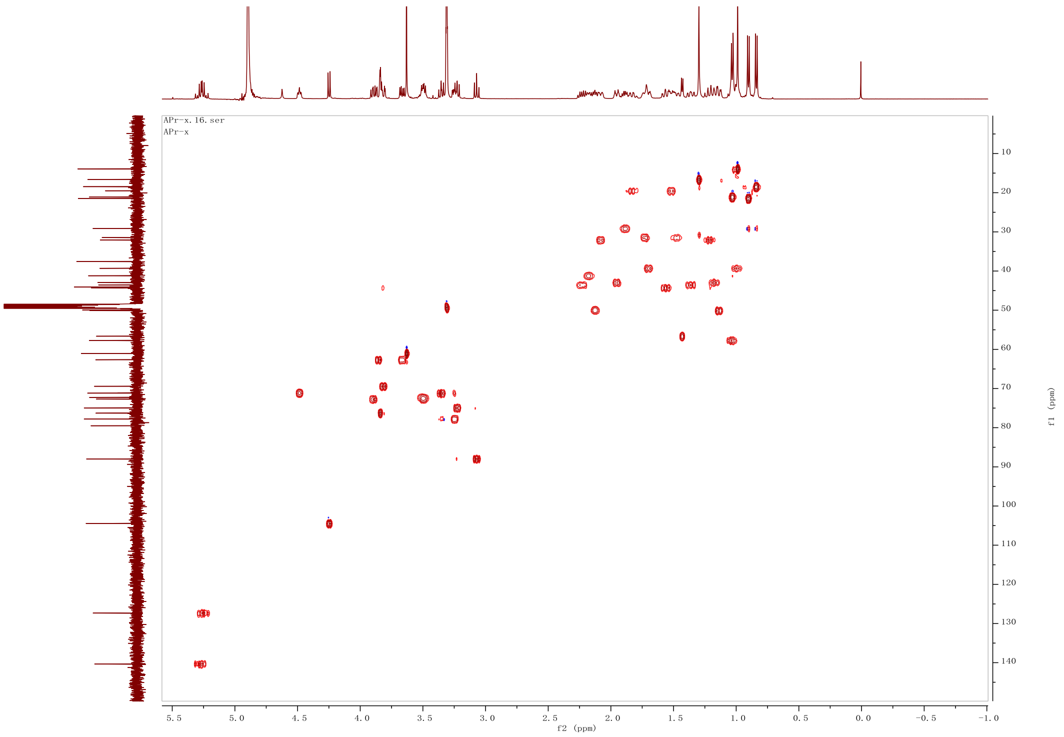

Figure S60 ^1H-^1H COSY spectrum of **7** in CD_3OD (500 MHz)

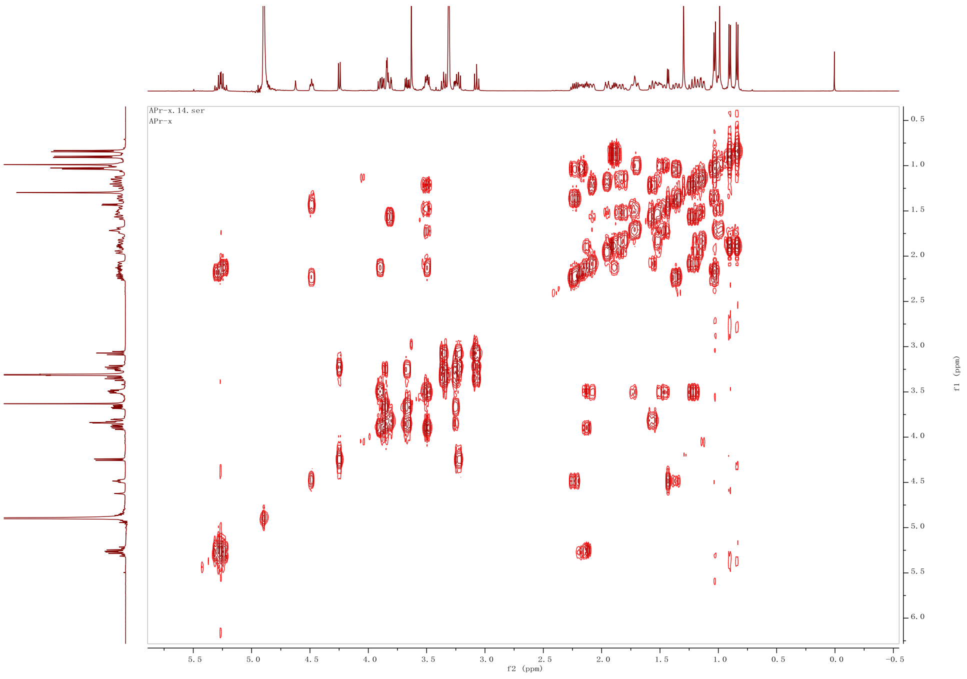

Figure S61 HMBC spectrum of **7** in CD_3OD (500 MHz)

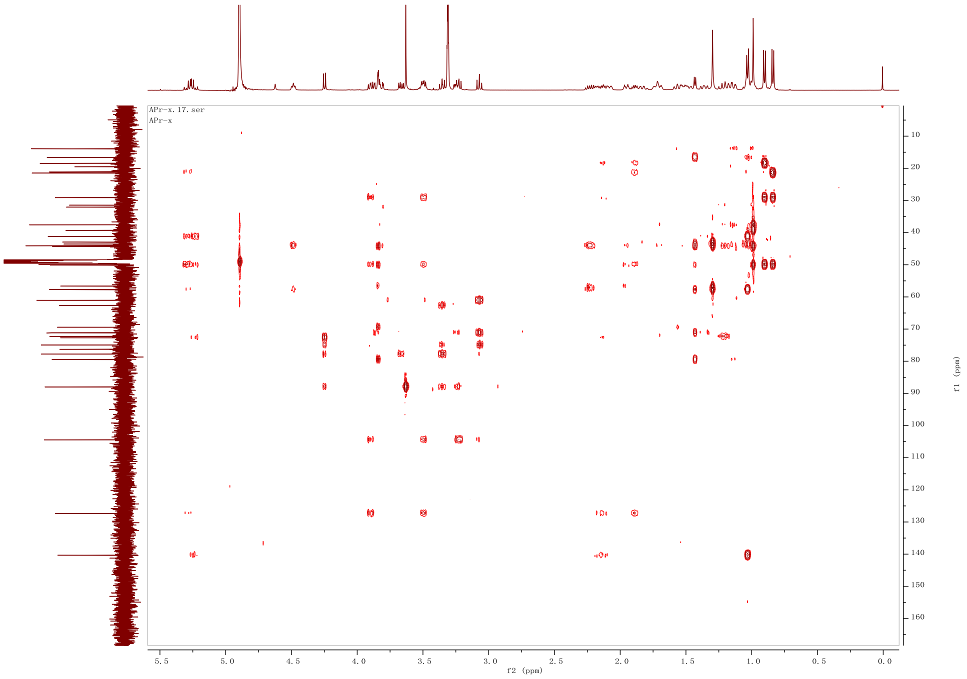

Figure S62 NOESY spectrum of **7** in CD_3OD (500 MHz)

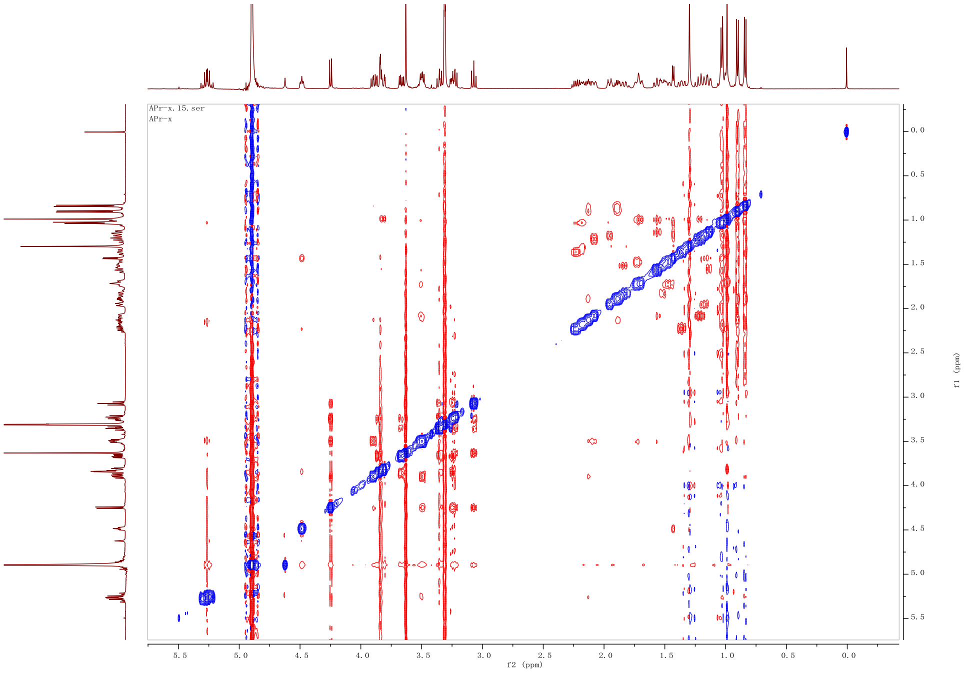

Figure S63 $[\alpha]_D$ data of **7**

Rudolph Research Analytical

This sample was measured on an Autopol VI, Serial #91058
Manufactured by Rudolph Research Analytical, Hackettstown, NJ, USA.

Measurement Date : Wednesday, 19-JUL-2023

Set Temperature : OFF

Time Delay : Disabled

Delay between Measurement : Disabled

n	Average	Std.Dev.	% RSD	Maximum	Minimum
5	-12.73	0.64	-5.02	-11.82	-13.64

S.No	Sample ID	Time	Result	Scale	OR °Arc	WLG.nm	Lg.mm	Conc.g/100ml	Temp.
1	APR-X	12:22:36 PM	-11.82	SR	-0.013	589	100.00	0.110	25.1
2	APR-X	12:22:42 PM	-12.73	SR	-0.014	589	100.00	0.110	25.1
3	APR-X	12:22:48 PM	-12.73	SR	-0.014	589	100.00	0.110	25.1
4	APR-X	12:22:55 PM	-12.73	SR	-0.014	589	100.00	0.110	25.1
5	APR-X	12:23:01 PM	-13.64	SR	-0.015	589	100.00	0.110	25.1

Figure S64 HR-ESI-MS and MS/MS spectra of **8**

Elemental Composition

File Edit View Process Help

Single Mass Analysis
Tolerance = 10.0 PPM / DBE: min = 0.5, max = 20.0
Element prediction: Off
Number of isotope peaks used for i-FIT = 3
Monoisotopic Mass, Even Electron Ions
351 formula(e) evaluated with 4 results within limits (all results (up to 1000) for each mass)
Elements Used:
C: 33-35 H: 0-100 O: 0-20 Na: 0-3 S: 0-2

Mass	Calc. Mass	mDa	PPM	DBE	Formula	i-FIT	i-FIT Norm	Fit Conf %	C	H	O	Na	S
707.3334	707.3313	2.1	3.0	6.5	C33 H55 O14 S	727.9	0.035	96.60	33	55	14		1
	707.3359	-2.5	-3.5	6.5	C34 H54 O11 Na3	736.3	8.432	0.02	34	54	11	3	

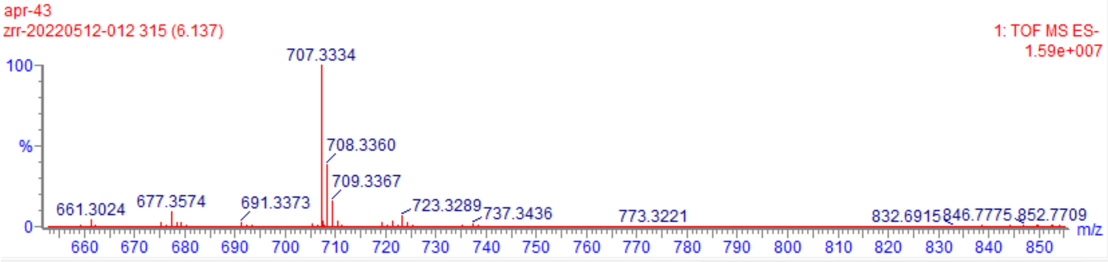

Elemental Composition

File Edit View Process Help

Single Mass Analysis
Tolerance = 10.0 PPM / DBE: min = 0.5, max = 20.0
Element prediction: Off
Number of isotope peaks used for i-FIT = 3
Monoisotopic Mass, Even Electron Ions
348 formula(e) evaluated with 5 results within limits (all results (up to 1000) for each mass)
Elements Used:
C: 33-35 H: 0-100 O: 0-20 Na: 0-3 S: 0-2

Mass	Calc. Mass	mDa	PPM	DBE	Formula	i-FIT	i-FIT Norm	Fit Conf %	C	H	O	Na	S
753.3111	753.3108	0.3	0.4	5.5	C33 H55 O14 Na2 S	728.6	1.380	25.15	33	55	14	2	1
	753.3132	-2.1	-2.8	8.5	C35 H54 O14 Na S	729.5	2.201	11.07	35	54	14	1	1

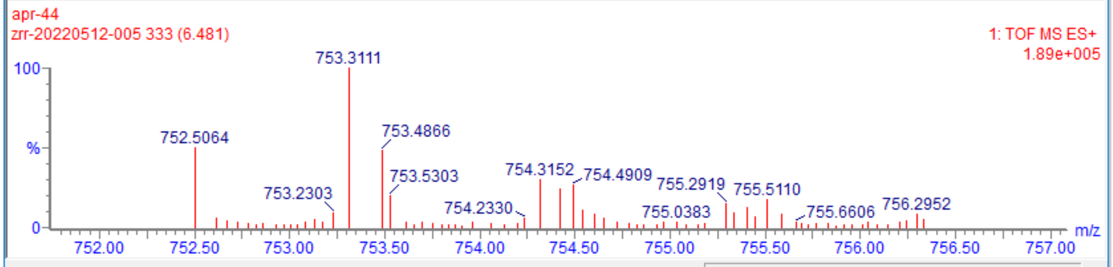

For Help, press F1

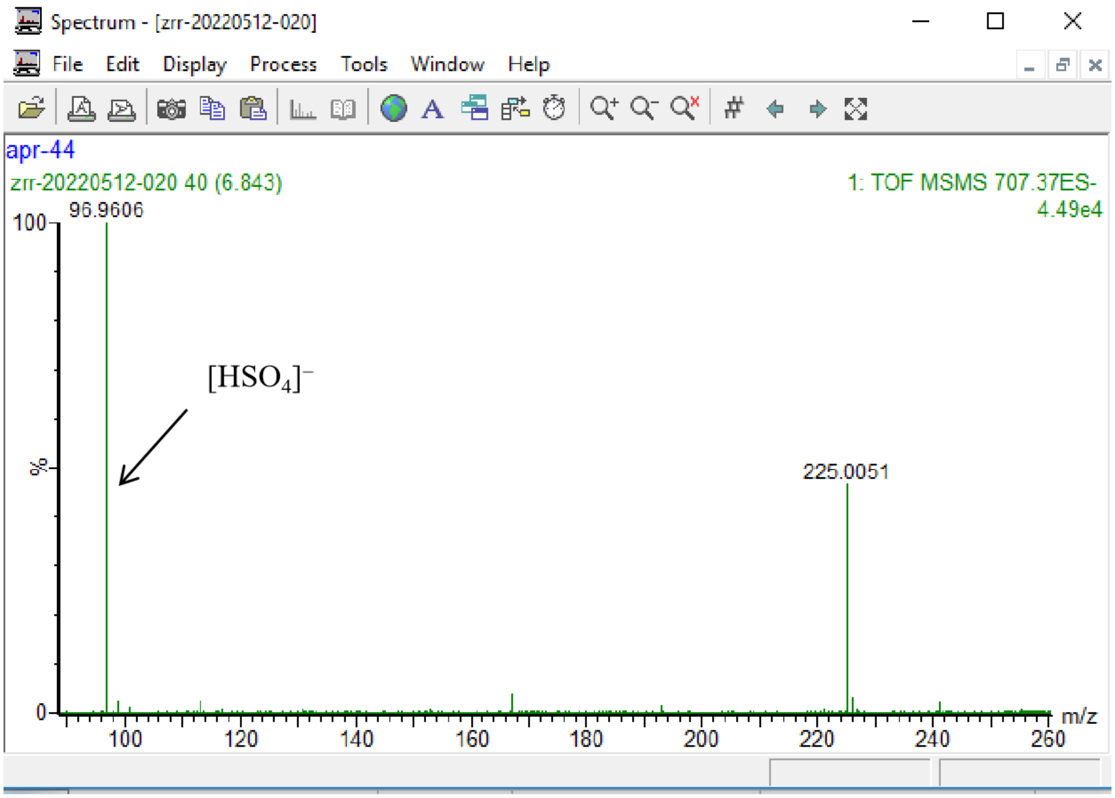
Spectrum - [zrr-20220512-020]
File Edit Display Process Tools Window Help
apr-44
zrr-20220512-020 40 (6.843)
1: TOF MSMS 707.37ES-
4.49e4
96.9606
$[HSO_4]^-$
225.0051
m/z

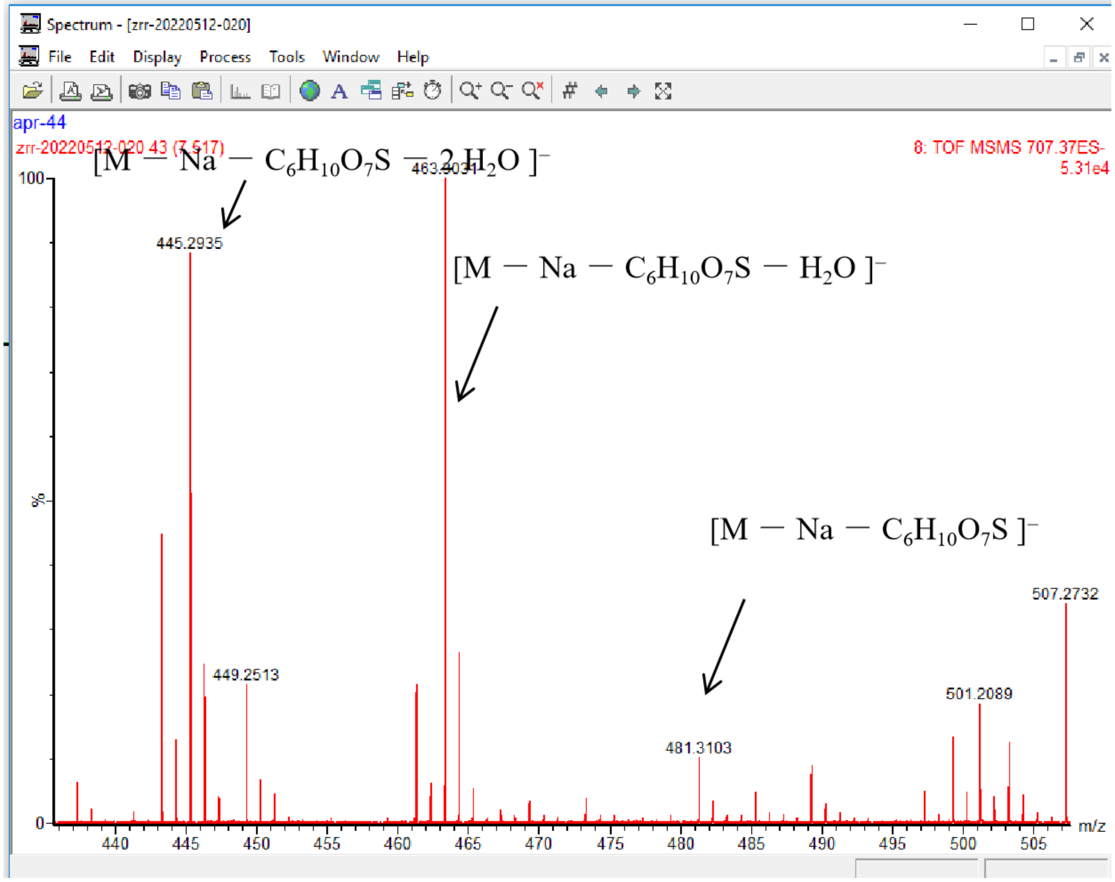
Spectrum - [zrr-20220512-020]
File Edit Display Process Tools Window Help
apr-44
zrr-20220512-020 43 (7.517)
8: TOF MSMS 707.37ES-
5.31e4
$[M - Na - C_6H_{10}O_7S - 2H_2O]^-$
463.3031
445.2935
$[M - Na - C_6H_{10}O_7S - H_2O]^-$
$[M - Na - C_6H_{10}O_7S]^-$
507.2732
449.2513
501.2089
481.3103
m/z

Figure S65 ^{1}H NMR spectrum of **8** in CD_3OD (500 MHz)

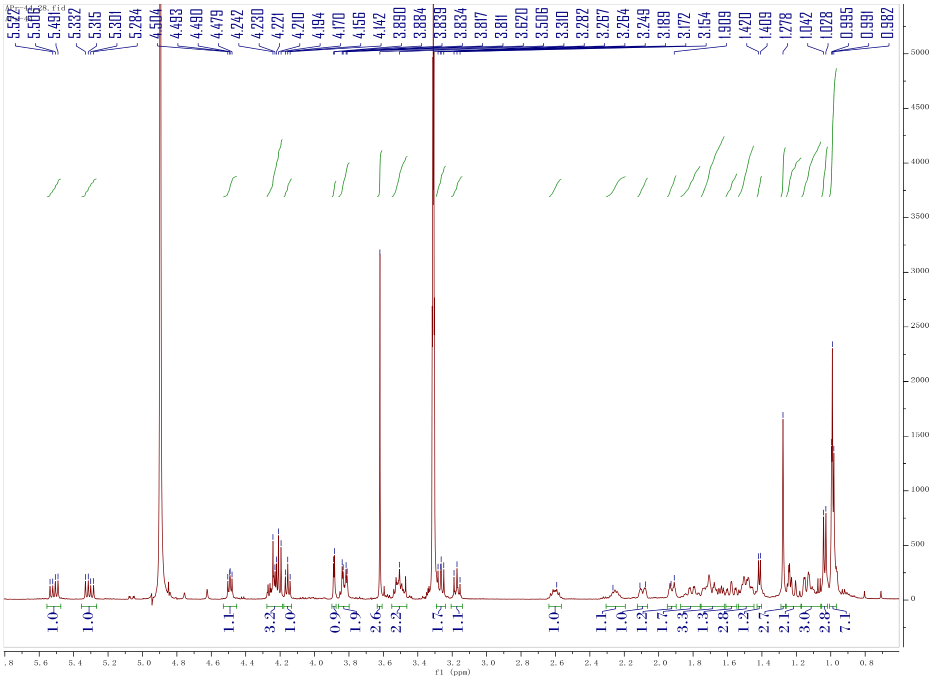

Figure S66 ^{13}C NMR spectra of **8** in CD_3OD (125 MHz)

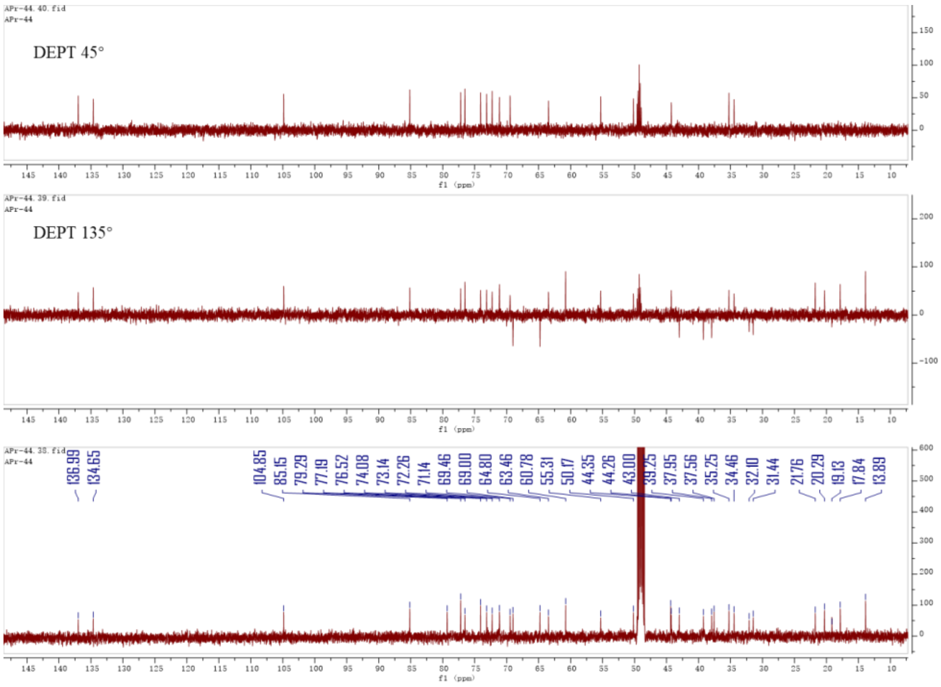

Figure S67 HSQC spectrum of **8** in CD_3OD (500 MHz)

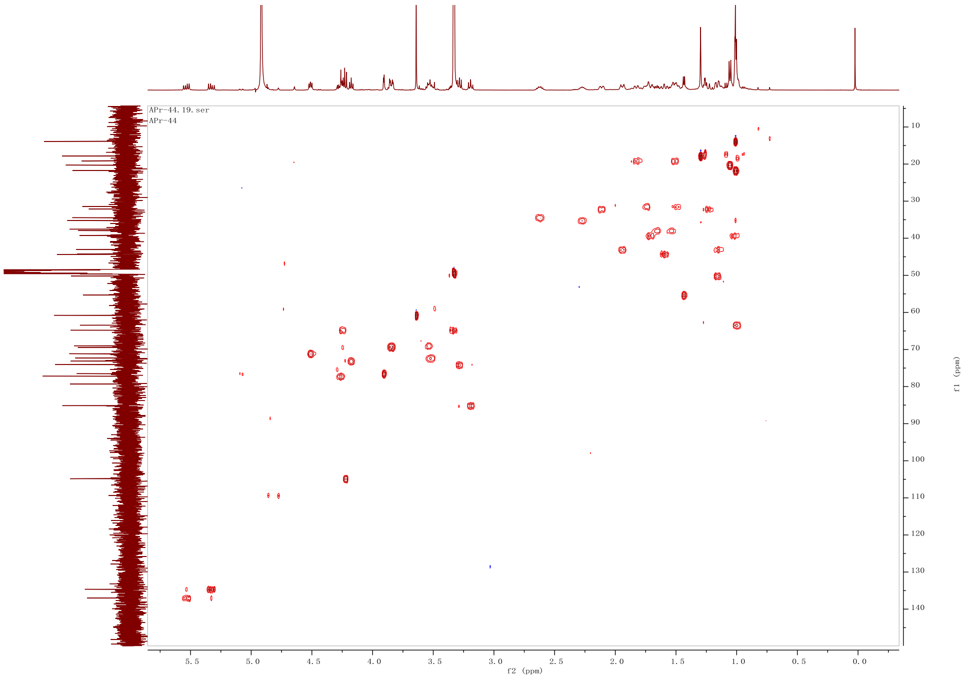

Figure S68 ^1H-^1H COSY spectrum of **8** in CD_3OD (500 MHz)

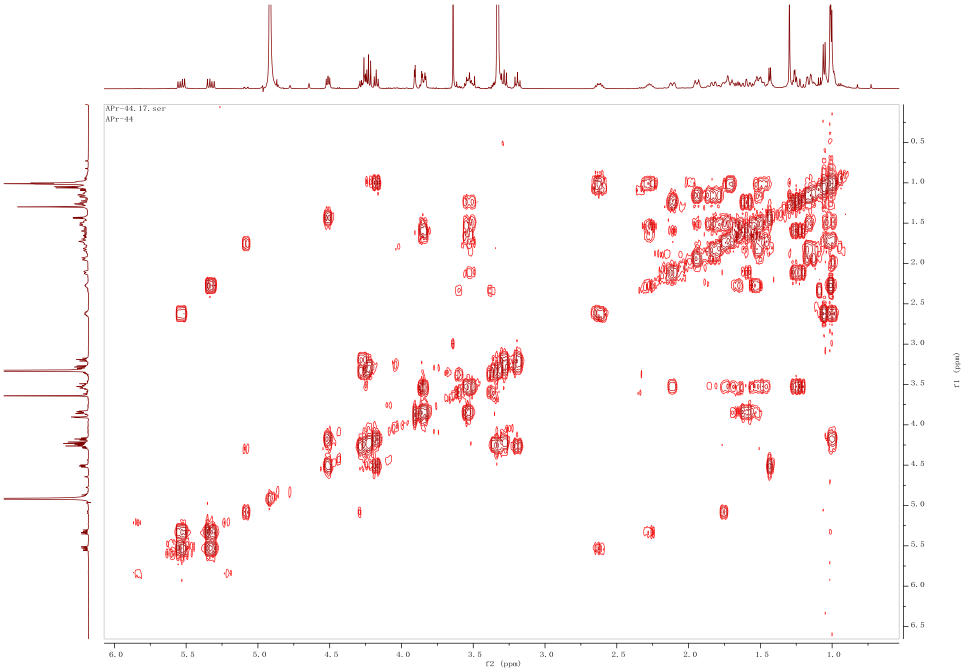

Figure S69 HMBC spectrum of **8** in CD_3OD (500 MHz)

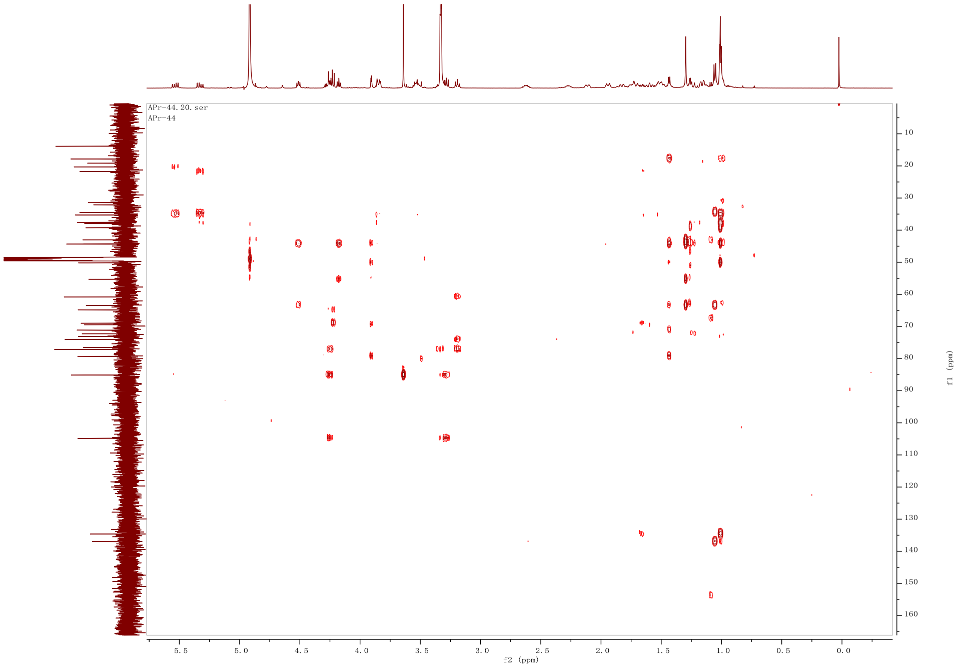

Figure S70 NOESY spectrum of **8** in CD_3OD (500 MHz)

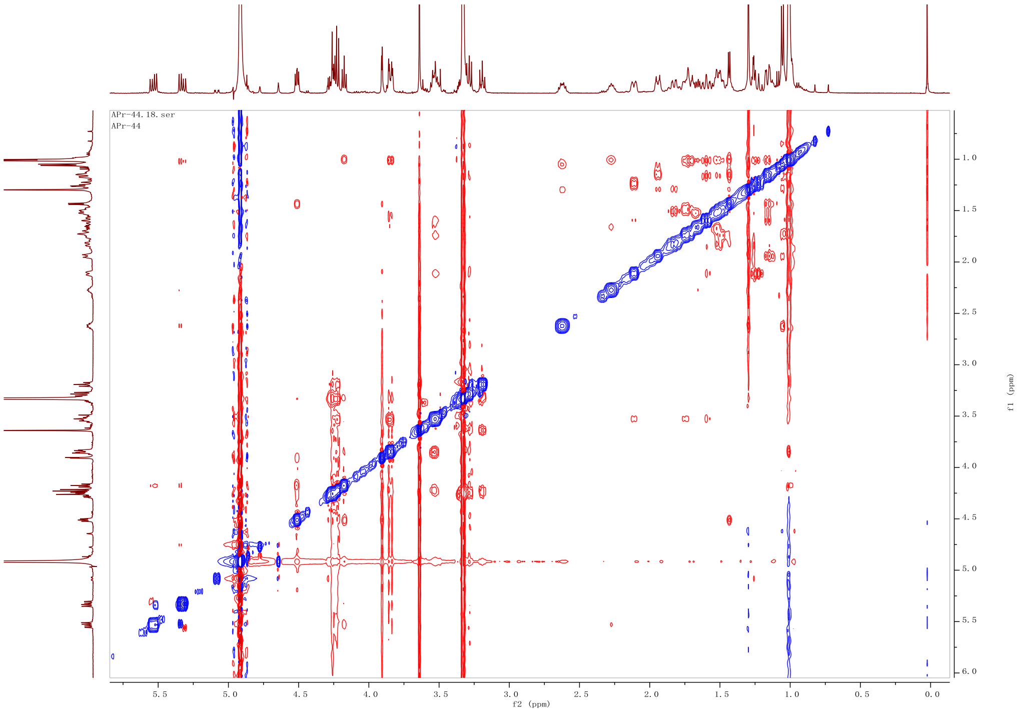

Figure S71 [α] $_D$ data of **8**

Rudolph Research Analytical

This sample was measured on an Autopol VI, Serial #91058
Manufactured by Rudolph Research Analytical, Hackettstown, NJ, USA.

Measurement Date : Tuesday, 18-JUL-2023

Set Temperature : 25.0

Time Delay : Disabled

Delay between Measurement : Disabled

n	Average	Std.Dev.	% RSD	Maximum	Minimum
5	10.44	0.61	5.84	11.11	10.00

S.No	Sample ID	Time	Result	Scale	OR °Arc	WLG.nm	Lg.mm	Conc.g/100ml	Temp.
1	APR-44	08:51:44 PM	10.00	SR	0.009	589	100.00	0.090	25.0
2	APR-44	08:51:50 PM	11.11	SR	0.010	589	100.00	0.090	25.0
3	APR-44	08:51:57 PM	11.11	SR	0.010	589	100.00	0.090	25.0
4	APR-44	08:52:03 PM	10.00	SR	0.009	589	100.00	0.090	25.0
5	APR-44	08:52:09 PM	10.00	SR	0.009	589	100.00	0.090	25.0

Figure S72 HR-ESI-MS and MS/MS spectra of **9**

Elemental Composition

File Edit View Process Help

Single Mass Analysis
Tolerance = 20.0 PPM / DBE: min = 0.5, max = 20.0
Element prediction: Off
Number of isotope peaks used for i-FIT = 3
Monoisotopic Mass, Even Electron Ions
184 formula(e) evaluated with 3 results within limits (all results (up to 1000) for each mass)
Elements Used:
C: 34-34 H: 0-70 O: 0-15 Na: 0-3 S: 0-2

Mass	Calc. Mass	mDa	PPM	DBE	Formula	i-FIT	i-FIT Norm	Fit Conf %	C	H	O	Na	S
721.3488	721.3469	1.9	2.6	6.5	C34 H57 O14 S	724.5	1.723	17.85	34	57	14		1
	721.3573	-8.5	-11.8	4.5	C34 H59 O11 Na2 S	724.6	1.864	15.51	34	59	11	2	1
	721.3396	9.2	12.8	4.5	C34 H59 O9 Na2 S2	723.2	0.406	66.65	34	59	9	2	2

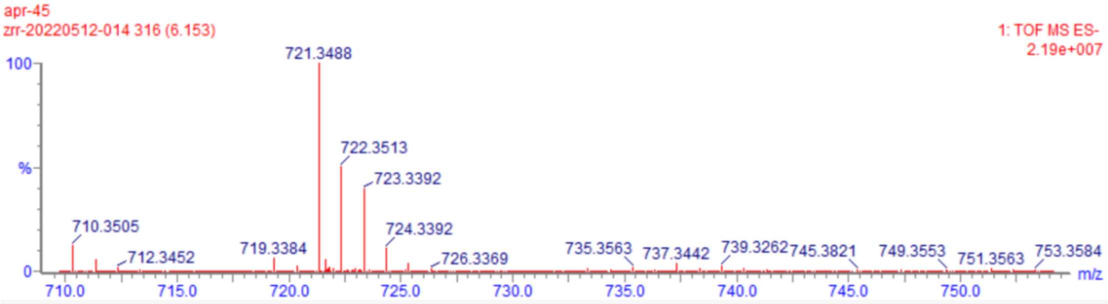

For Help, press F1

Elemental Composition

File Edit View Process Help

Single Mass Analysis
Tolerance = 20.0 PPM / DBE: min = 0.5, max = 20.0
Element prediction: Off
Number of isotope peaks used for i-FIT = 3
Monoisotopic Mass, Even Electron Ions
188 formula(e) evaluated with 1 results within limits (all results (up to 1000) for each mass)
Elements Used:
C: 34-34 H: 0-70 O: 0-15 Na: 0-3 S: 0-2

Mass	Calc. Mass	mDa	PPM	DBE	Formula	i-FIT	i-FIT Norm	Fit Conf %	C	H	O	Na	S
767.3275	767.3264	1.1	1.4	5.5	C34 H57 O14 Na2 S	751.2	n/a	n/a	34	57	14	2	1

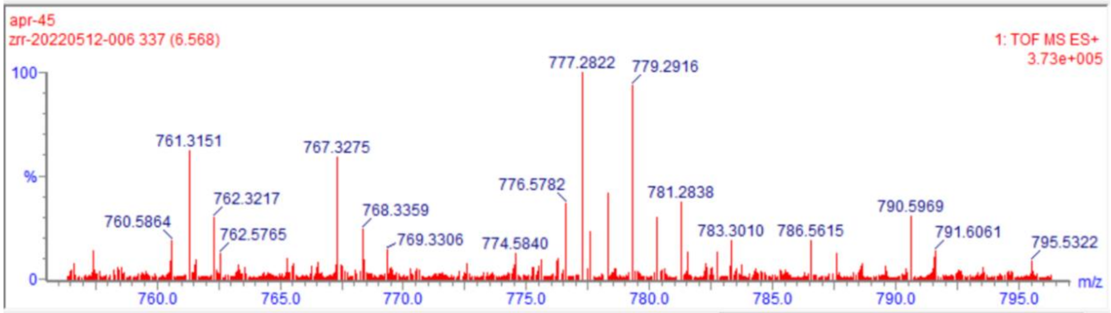

For Help, press F1

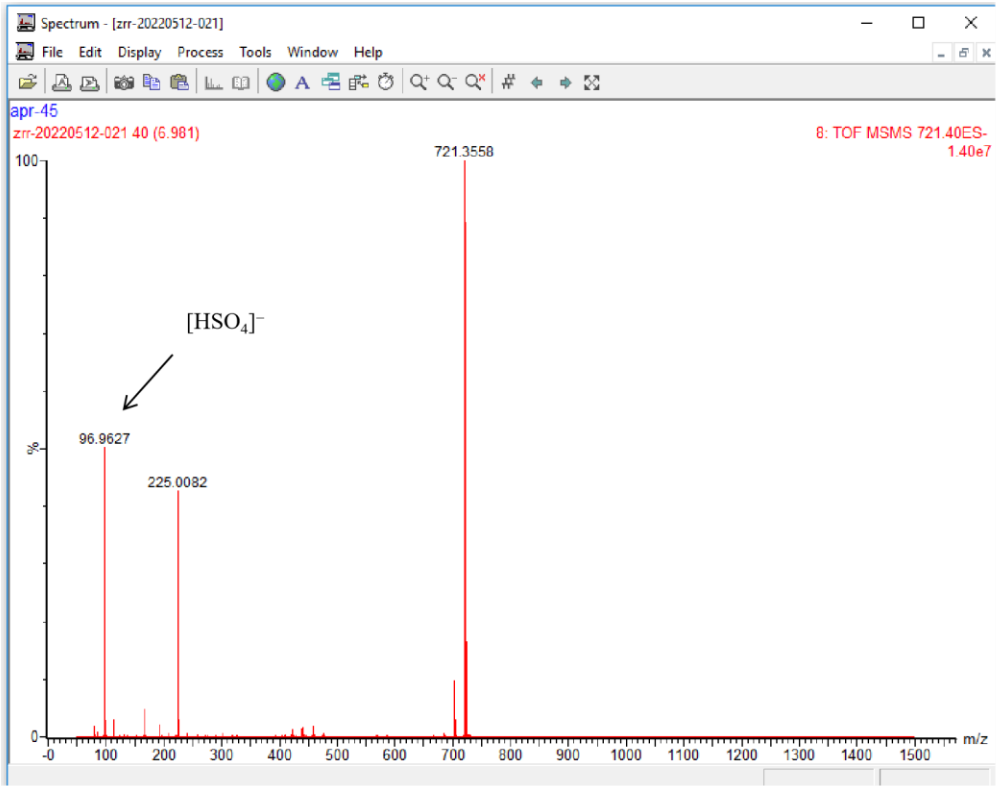
Spectrum - [zrr-20220512-021]
File Edit Display Process Tools Window Help
apr-45
zrr-20220512-021 40 (6.981)
8: TOF MSMS 721.40ES-
1.40e7
721.3558
$[HSO_4]^-$
96.9627
225.0082
%
m/z

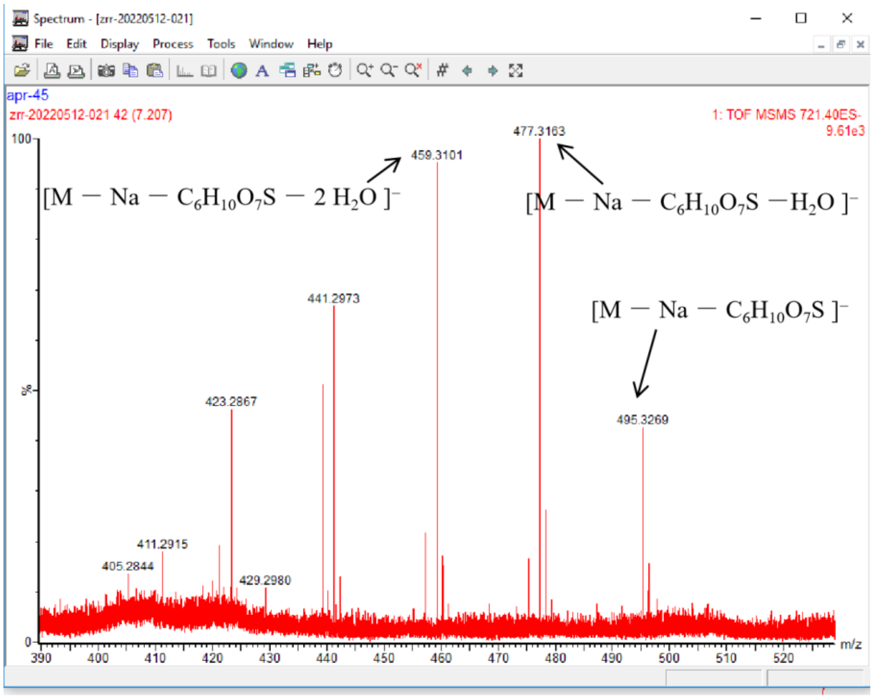
Spectrum - [zrr-20220512-021]
File Edit Display Process Tools Window Help
apr-45
zrr-20220512-021 42 (7.207)
1: TOF MSMS 721.40ES-
9.61e3
459.3101
477.3163
$[M - Na - C_6H_{10}O_7S - 2\ H_2O]^-$
$[M - Na - C_6H_{10}O_7S - H_2O]^-$
441.2973
$[M - Na - C_6H_{10}O_7S]^-$
423.2867
495.3269
411.2915
405.2844
429.2980
%
m/z

Figure S73 ^{1}H NMR spectrum of **9** in CD_3OD (500 MHz)

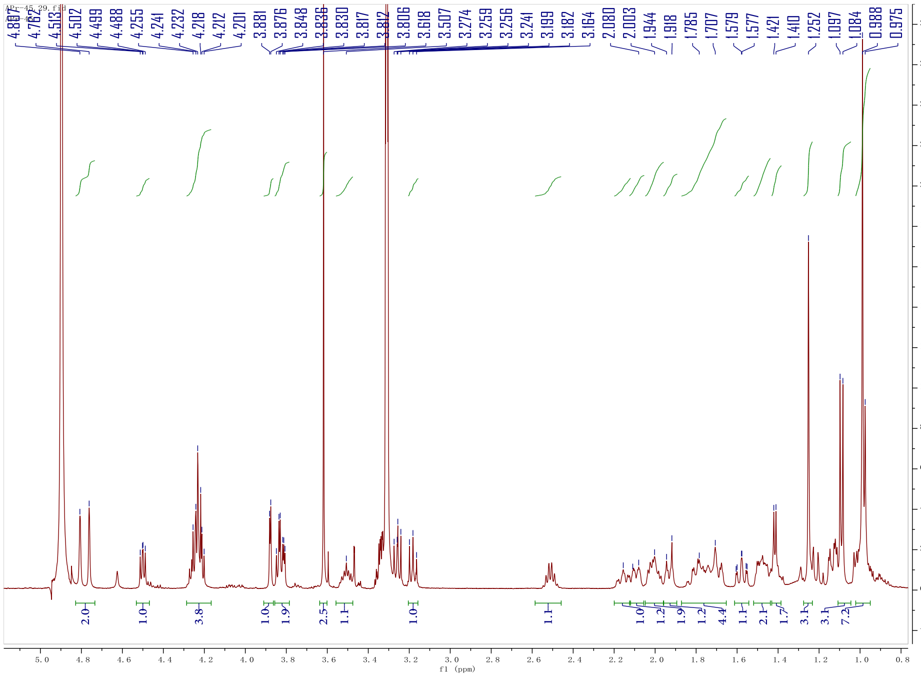

Figure S74 ^{13}C NMR spectra of **9** in CD_3OD (125 MHz)

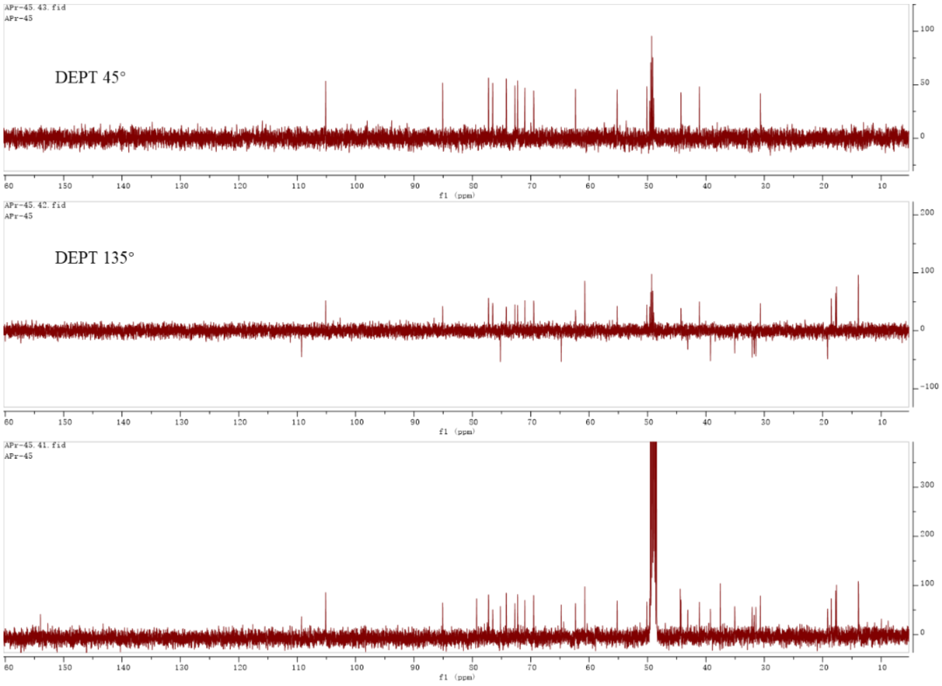

Figure S75 HSQC spectrum of **9** in CD_3OD (500 MHz)

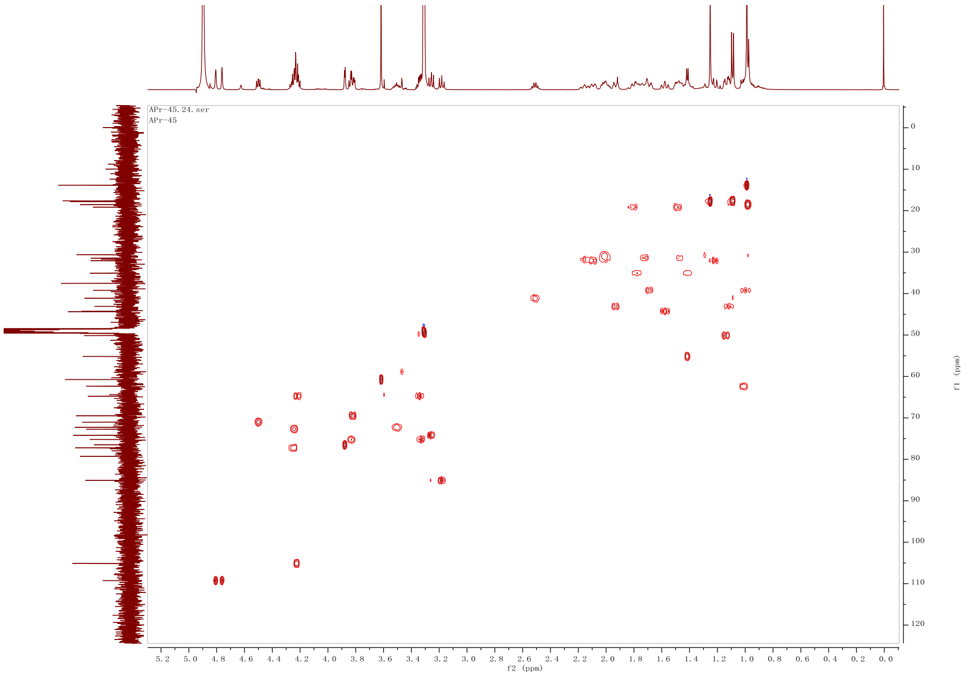

Figure S76 ^1H-^1H COSY spectrum of **9** in CD_3OD (500 MHz)

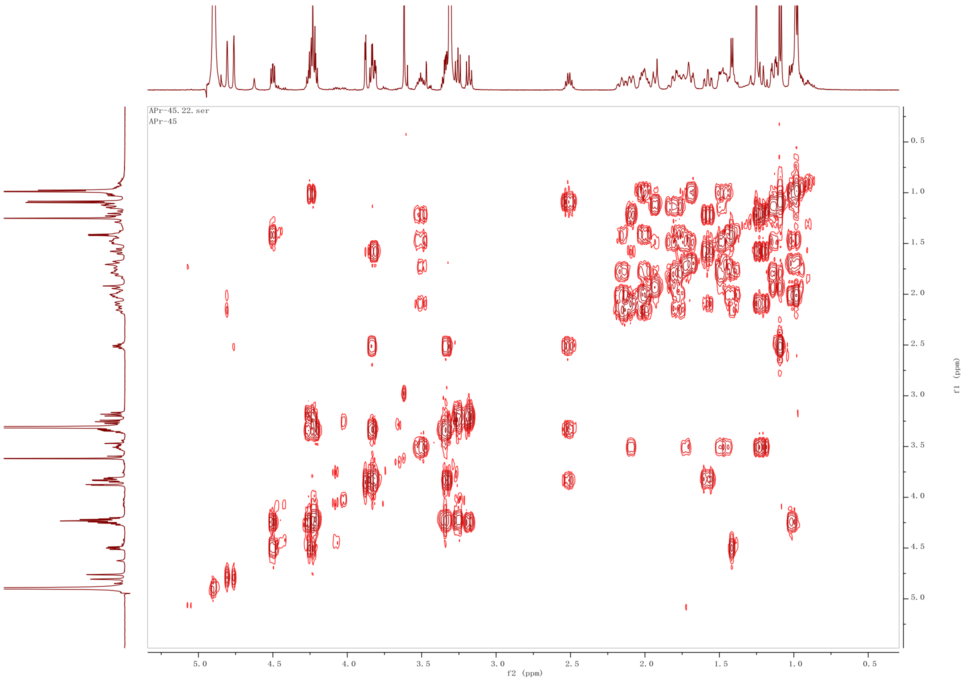

Figure S77 HMBC spectrum of **9** in CD_3OD (500 MHz)

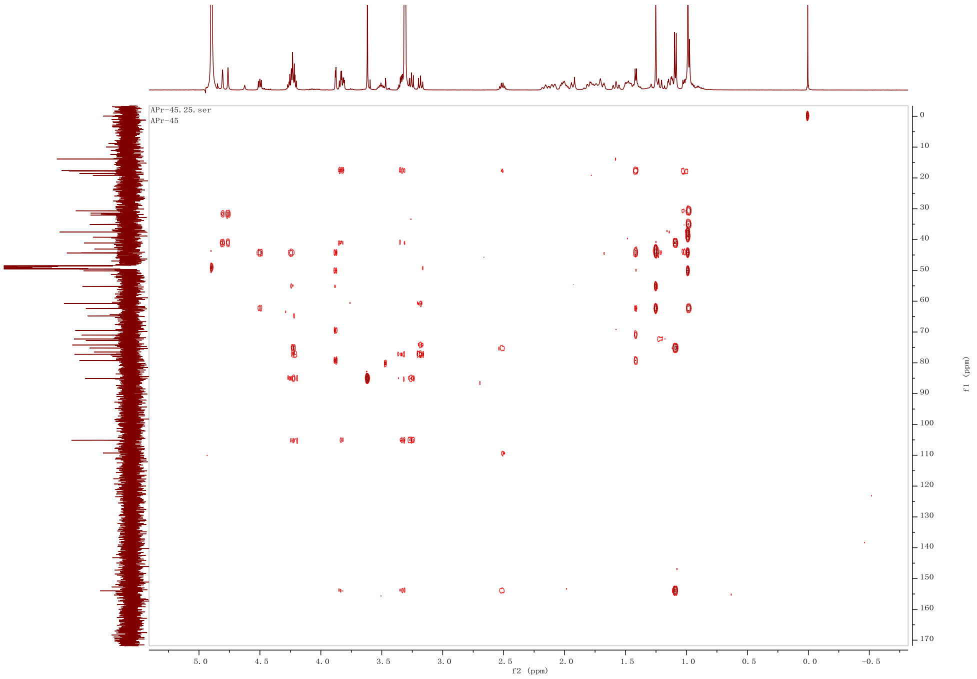

Figure S78 NOESY spectrum of **9** in CD_3OD (500 MHz)

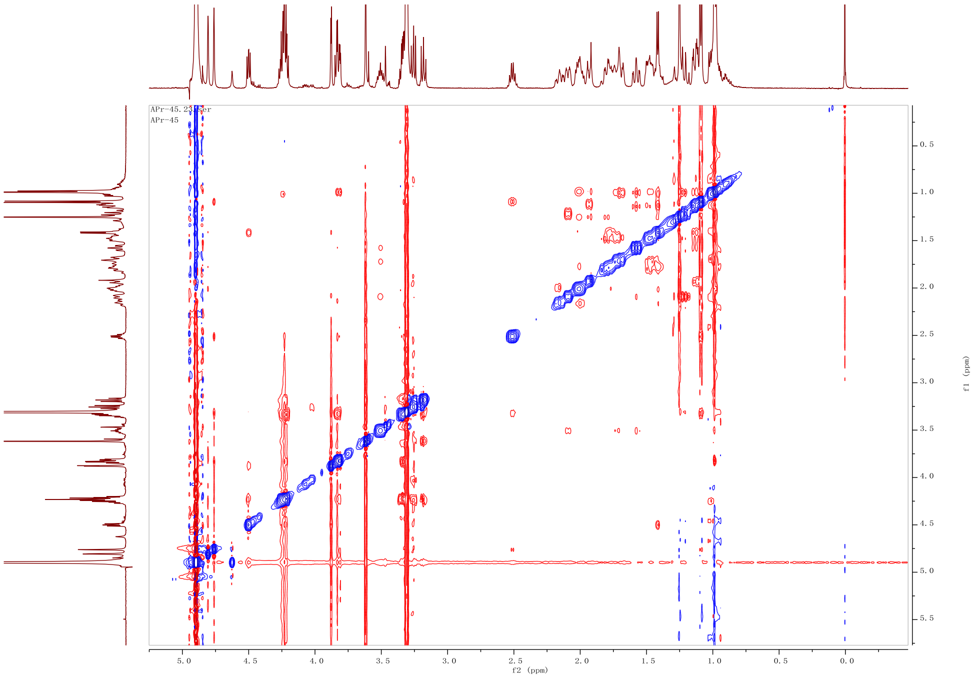

Figure S79 [α] $_D$ data of **9**

Rudolph Research Analytical

This sample was measured on an Autopol VI, Serial #91058
Manufactured by Rudolph Research Analytical, Hackettstown, NJ, USA.

Measurement Date : Tuesday, 18-JUL-2023

Set Temperature : 25.0

Time Delay : Disabled

Delay between Measurement : Disabled

n	Average	Std.Dev.	% RSD	Maximum	Minimum
5	7.50	0.00	0.00	7.50	7.50

S.No	Sample ID	Time	Result	Scale	OR °Arc	WLG.nm	Lg.mm	Conc.g/100ml	Temp.
1	APR-45	08:57:23 PM	7.50	SR	0.009	589	100.00	0.120	25.0
2	APR-45	08:57:30 PM	7.50	SR	0.009	589	100.00	0.120	25.0
3	APR-45	08:57:36 PM	7.50	SR	0.009	589	100.00	0.120	25.0
4	APR-45	08:57:42 PM	7.50	SR	0.009	589	100.00	0.120	25.0
5	APR-45	08:57:49 PM	7.50	SR	0.009	589	100.00	0.120	25.0

Figure S80 HR-ESI-MS and MS/MS spectra of **10**

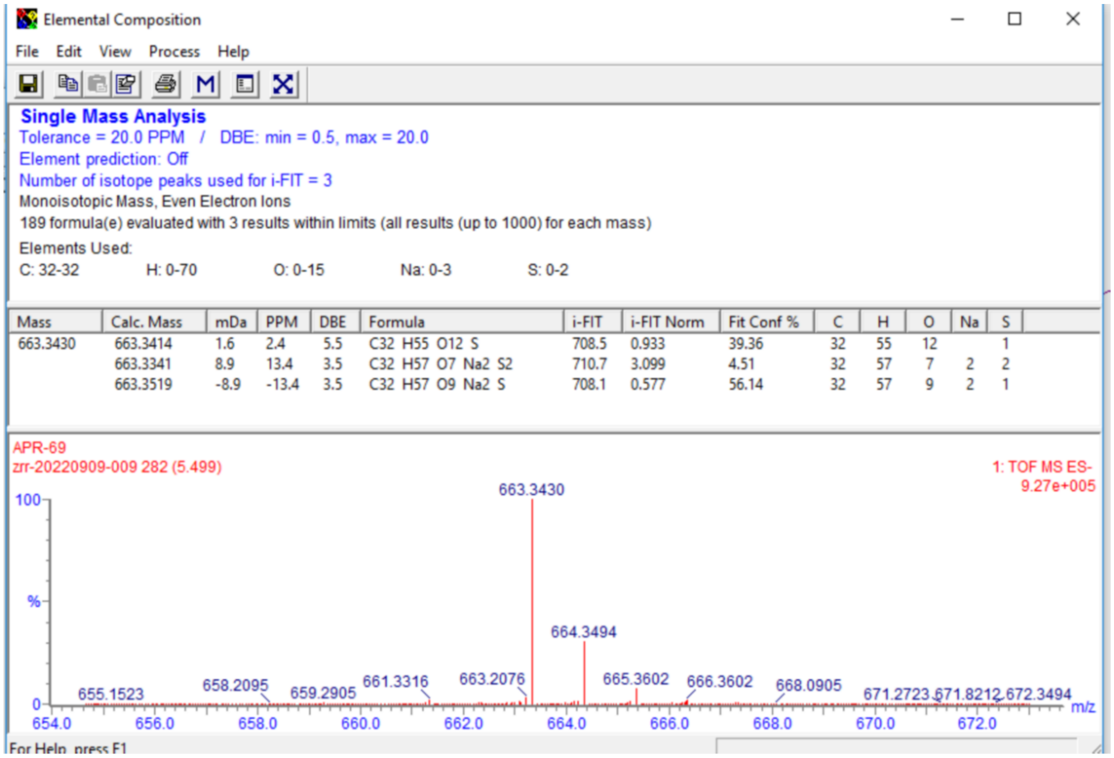

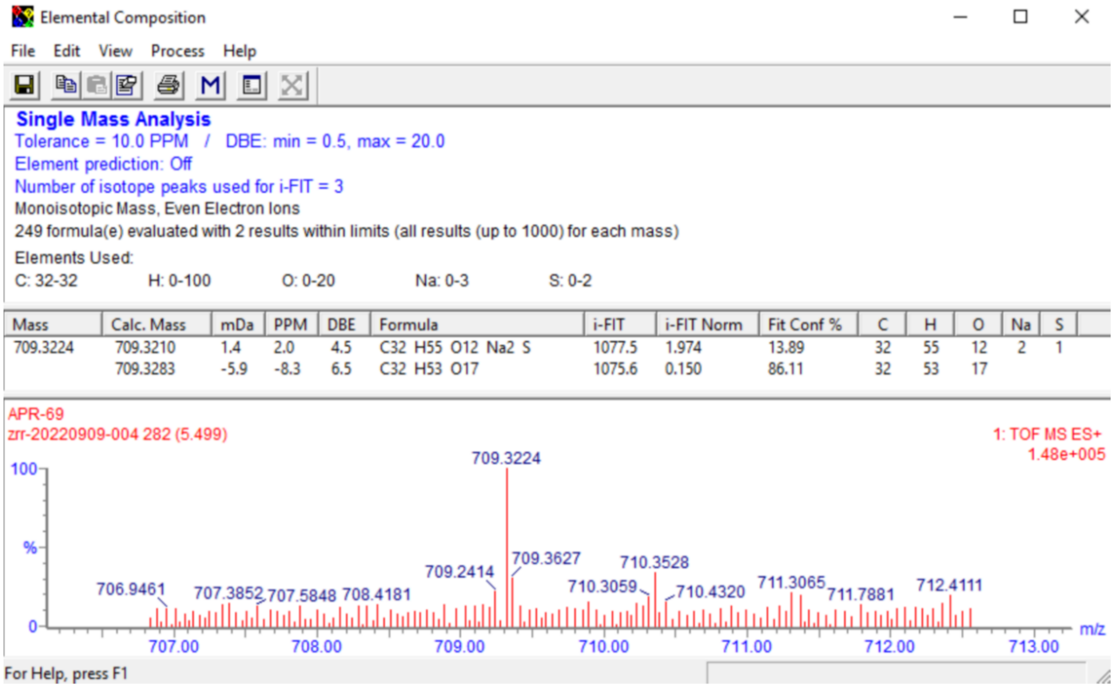

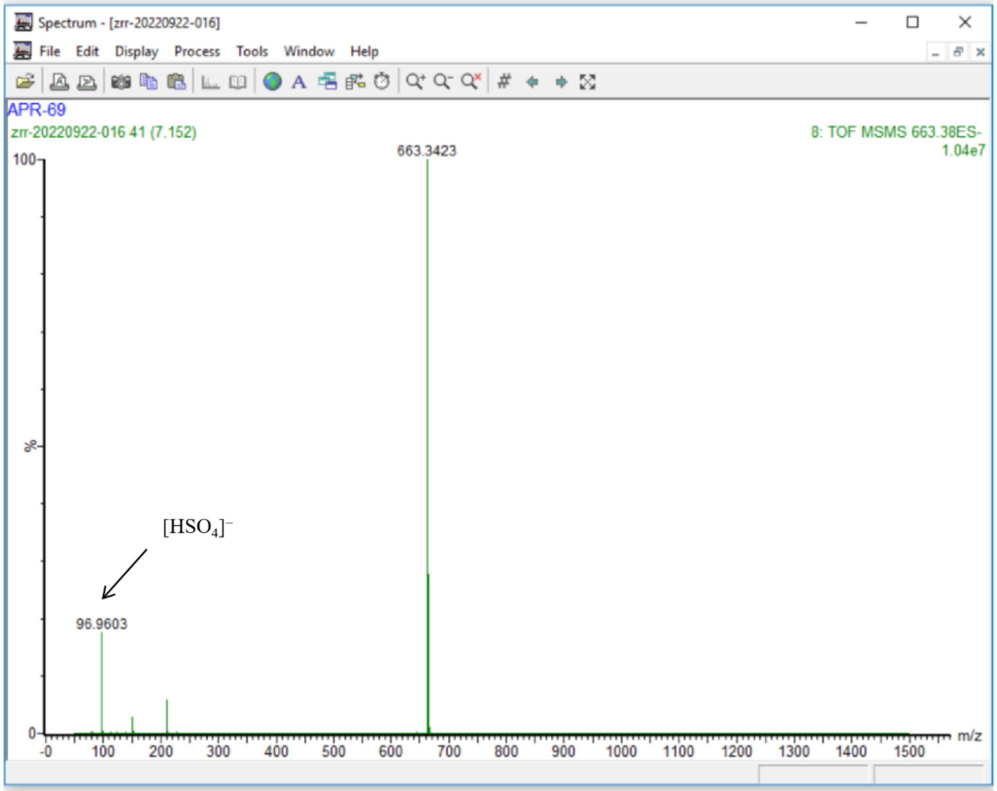
Spectrum - [zrr-20220922-016]
File Edit Display Process Tools Window Help
APR-69
zrr-20220922-016 41 (7.152)
8: TOF MSMS 663.38ES-
1.04e7
663.3423
[HSO4]−
96.9603
%
m/z

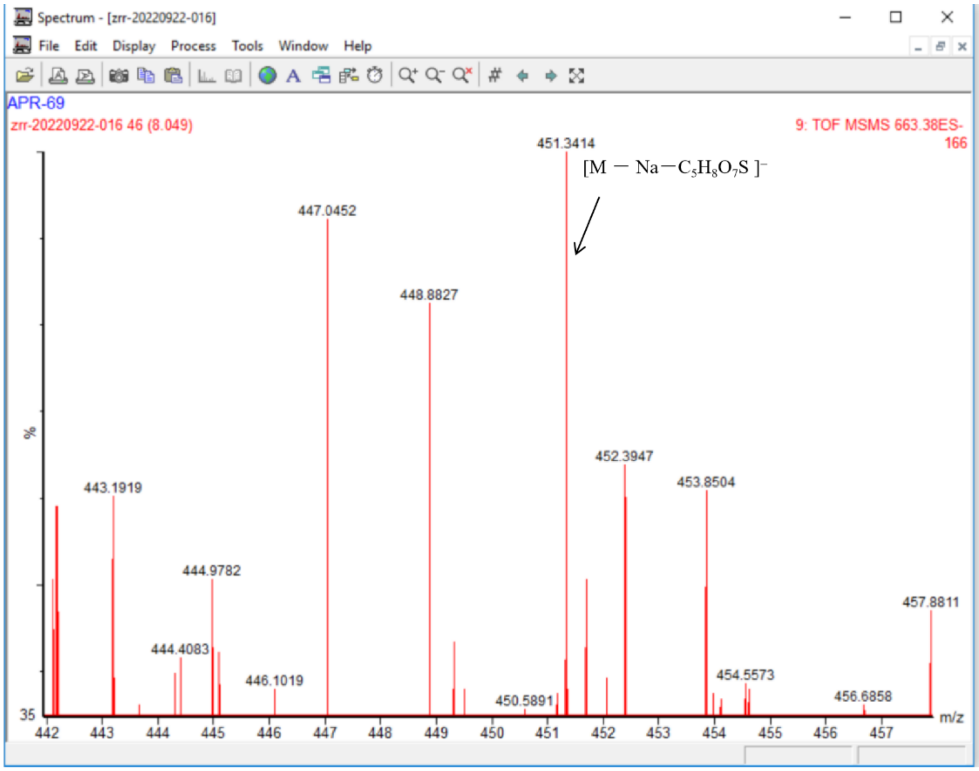
Spectrum - [zrr-20220922-016]
File Edit Display Process Tools Window Help
APR-69
zrr-20220922-016 46 (8.049)
9: TOF MSMS 663.38ES-
166
451.3414
[M − Na−C5H8O7S]−
447.0452
448.8827
452.3947
453.8504
443.1919
444.9782
444.4083
446.1019
450.5891
454.5573
456.6858
457.8811
%
m/z

Figure S81 ^{1}H NMR spectrum of **10** in CD_3OD (500 MHz)

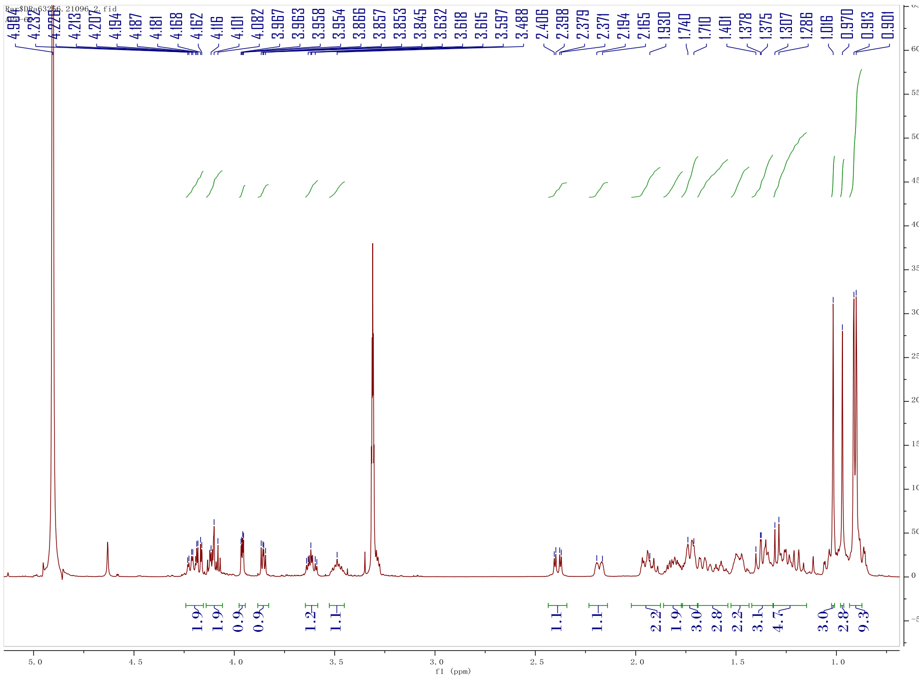

Figure S82 ^{13}C NMR spectra of **10** in CD_3OD (125 MHz)

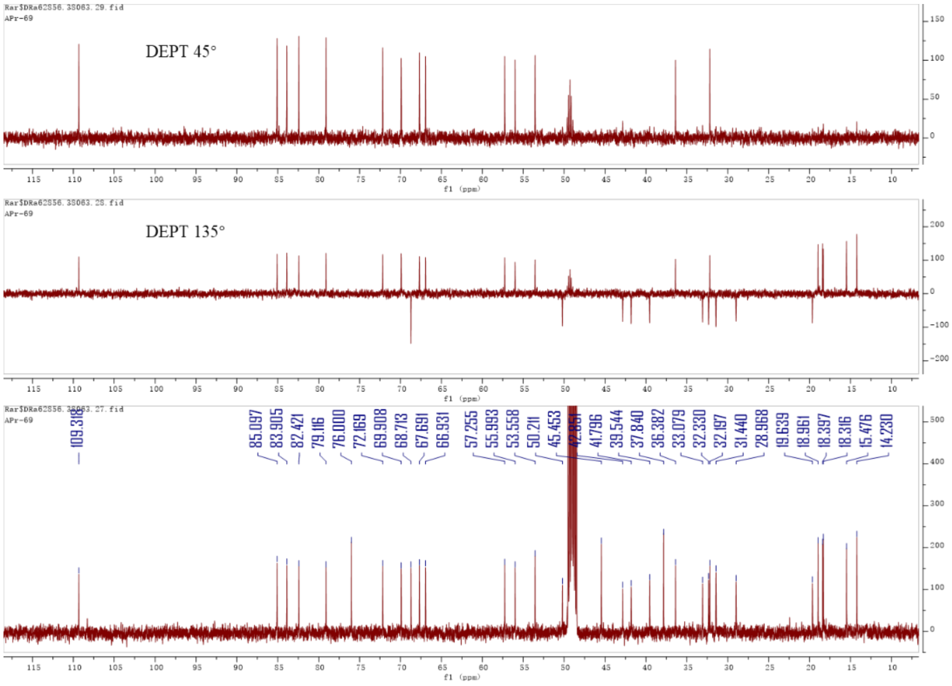

Figure S83 HSQC spectrum of **10** in CD_3OD (500 MHz)

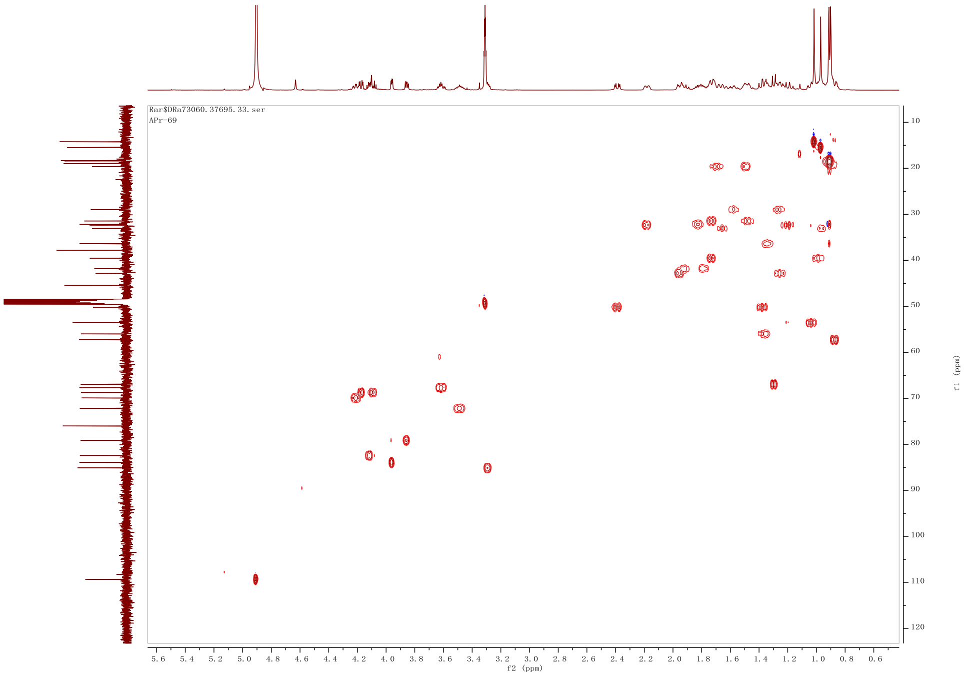

Figure S84 ^1H-^1H COSY spectrum of **10** in CD_3OD (500 MHz)

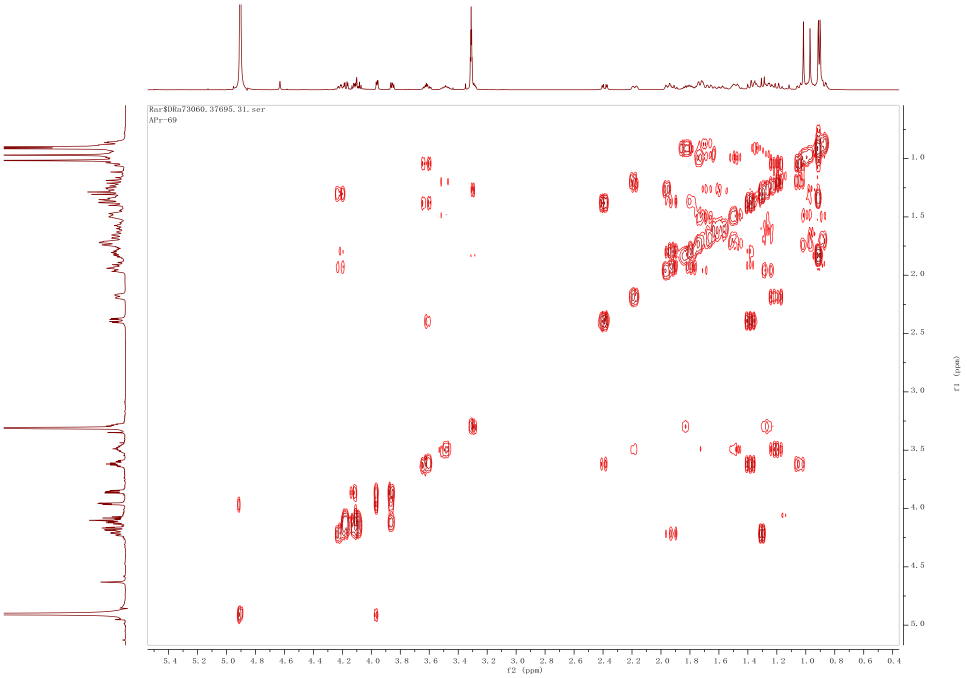

Figure S85 HMBC spectrum of **10** in CD_3OD (500 MHz)

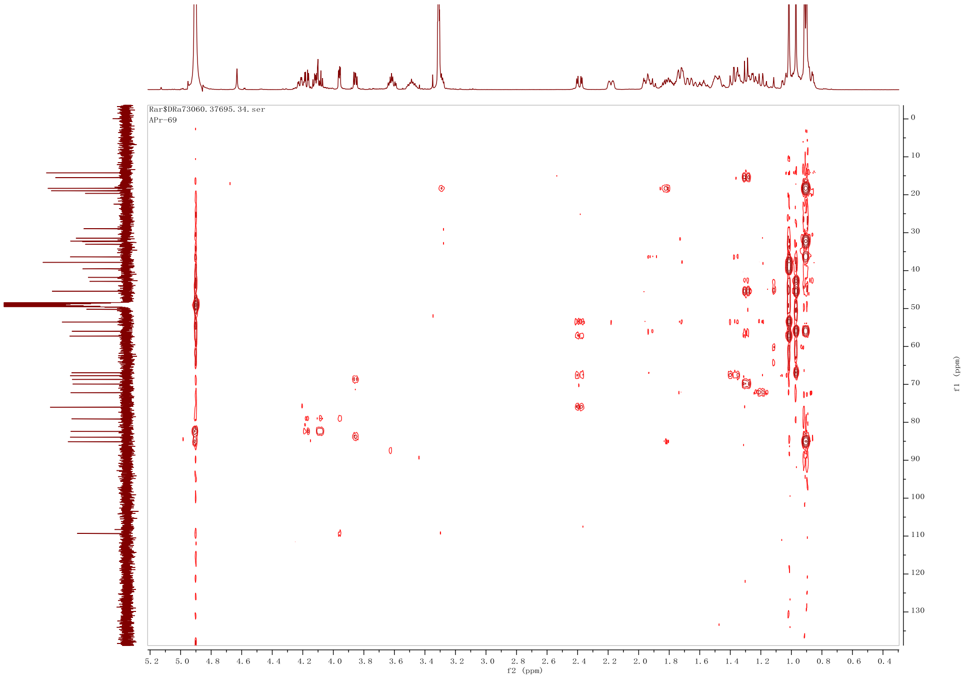

Figure S86 NOESY spectrum of **10** in CD_3OD (500 MHz)

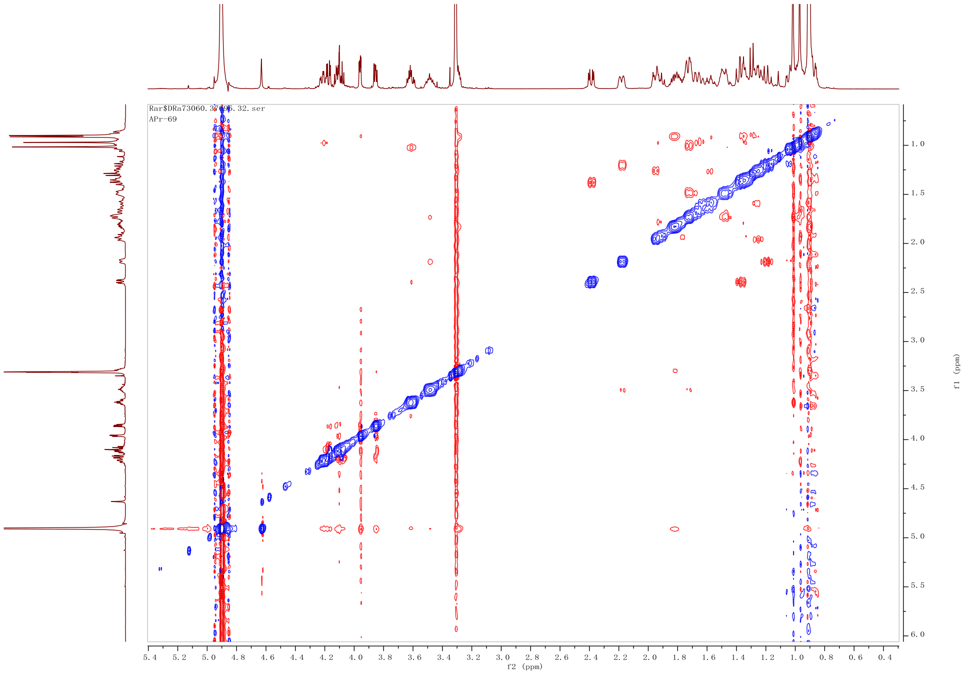

Figure S87 NOESY spectrum of **10** in $(CD_3)_2SO$ (500 MHz)

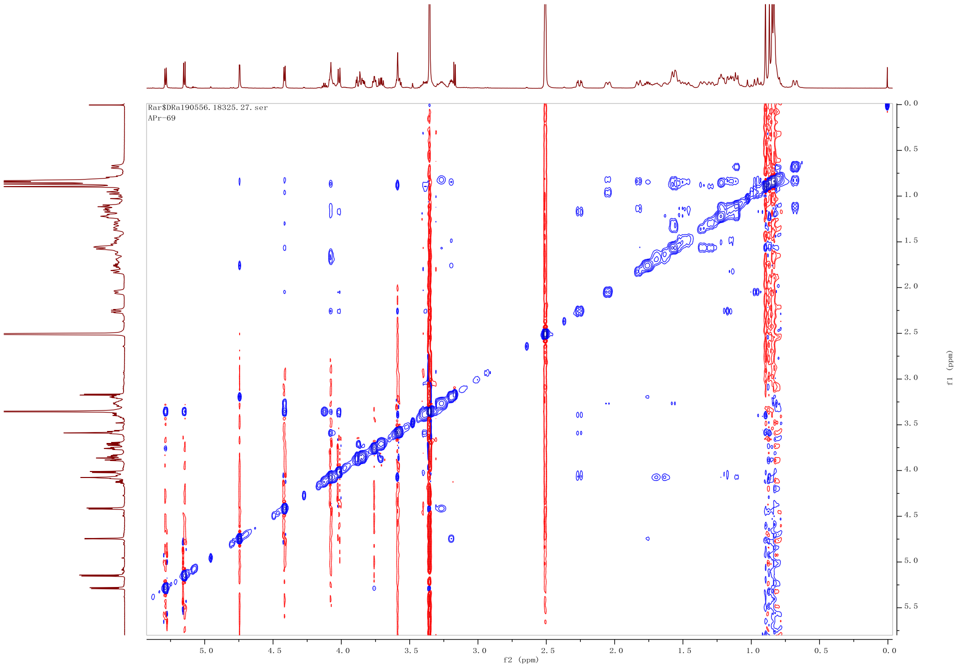

Figure S88 $[\alpha]_D$ data of **10**

Rudolph Research Analytical

This sample was measured on an Autopol VI, Serial #91058
Manufactured by Rudolph Research Analytical, Hackettstown, NJ, USA.

Measurement Date : Wednesday, 18-OCT-2023

Set Temperature : OFF

Time Delay : Disabled

Delay between Measurement : Disabled

n	Average	Std.Dev.	% RSD	Maximum	Minimum
5	8.16	0.37	4.53	8.33	7.50

S.No	Sample ID	Time	Result	Scale	OR °Arc	WLG.nm	Lg.mm	Conc.g/100ml	Temp.
1	APR-69	11:30:18 AM	8.33	SR	0.010	589	100.00	0.120	22.2
2	APR-69	11:30:25 AM	8.33	SR	0.010	589	100.00	0.120	22.2
3	APR-69	11:30:31 AM	7.50	SR	0.009	589	100.00	0.120	22.2
4	APR-69	11:30:38 AM	8.33	SR	0.010	589	100.00	0.120	22.2
5	APR-69	11:30:45 AM	8.33	SR	0.010	589	100.00	0.120	22.2

Table S1 The inhibitory effects of compounds **1-6** and **10** on nitric oxide (NO) production in RAW264.7 cell activated by LPS.

Compounds	Concentration （μM）	Inhibition rate（%）
L-NMMA	50 μM	60.32±3.70
**1**	50 μM	11.09±0.70
**2**	50 μM	6.26±3.33
**3**	50 μM	0.39±0.16
**4**	50 μM	3.51±2.42
**5**	50 μM	2.36±1.86
**6**	50 μM	1.57±3.99
**10**	50 μM	-0.86±2.64
